# *MA’AT* analysis of the *O*-glycosidic linkages of oligosaccharides using nonconventional NMR *J*-couplings: *MA’AT* and MD models of *phi*†

**DOI:** 10.1039/d4ra06062h

**Published:** 2024-09-23

**Authors:** Reagan J. Meredith, Wenhui Zhang, Mi-Kyung Yoon, Xiaosong Hu, Ian Carmichael, Anthony S. Serianni

**Affiliations:** a Department of Chemistry and Biochemistry, University of Notre Dame Notre Dame IN 46556 USA aseriann@nd.edu; b Texas Biomedical Research Institute San Antonio TX 78227 USA; c Omicron Biochemicals, Inc. South Bend IN 46617 USA; d Department of Chemistry, Wuhan University of Technology Wuhan 430070 China; e Radiation Laboratory, University of Notre Dame Notre Dame IN 46556 USA

## Abstract

*MA’AT* analysis (Meredith *et al.*, *J. Chem. Inf. Model.* 2022, **62**, 3135–3141) is a new NMR-based method to treat ensembles of redundant NMR spin-coupling constants (*J*-couplings) to obtain experiment-based probability distributions of molecular torsion angles in solution. Work reported to date on modeling the conformations of *O*-glycosidic linkages of oligosaccharides using three conventional *J*-coupling constraints (^2^*J*_COC_, ^3^*J*_COCH_, ^3^*J*_COCC_) has shown that the method gives mean torsion angles and circular standard deviations (CSDs) for *psi* in very good agreement with those obtained by MD simulation. On the other hand, CSDs for *phi* determined by *MA’AT* analysis have consistently been much larger than those determined by MD, calling into question either the reliability of *MA’AT* analysis or MD to accurately predict this behavior. Prior work has shown that this discrepancy does not stem from the limitations of DFT-based *J*-coupling equation parameterization where secondary conformational dependencies can introduce uncertainties. The present work re-visits this problem by incorporating a new nonconventional *J*-coupling constraint into *MA’AT* analyses of *phi*, namely, a geminal (two-bond) ^2^*J*_CCH_*J*-value that exhibits a strong primary dependence on *phi*. The latter property pertains explicitly to linkages contributed by GlcNAc pyranosyl rings and pyranosyl rings devoid of substituents at C2 (*i.e.*, deoxy residues) where known secondary contributions to ^2^*J*_CCH_ magnitude caused by C–O bond rotation involving the coupled carbon are negligible or absent. The results show that when ^2^*J*_CCH_ values are added to the analysis, *phi* CSDs reduce considerably, bringing them into better alignment with those obtained by MD simulation. The cause of the discrepancy when only three conventional *J*-couplings are used to treat *phi* appears to be associated with the two-bond ^2^*J*_COC_, which has properties that make it less effective than the non-conventional ^2^*J*_CCH_ as a discriminator of different conformational models of *phi*.

## Abstract 2

The use of two-bond ^13^C–^1^H spin-coupling constants as nonconventional constraints in *MA'AT* analyses of the *phi* torsion angles of *O*-glycosidic linkages improves the agreement between *MA'AT*- and MD-determined probability distributions of the angles.

## Graphical abstract

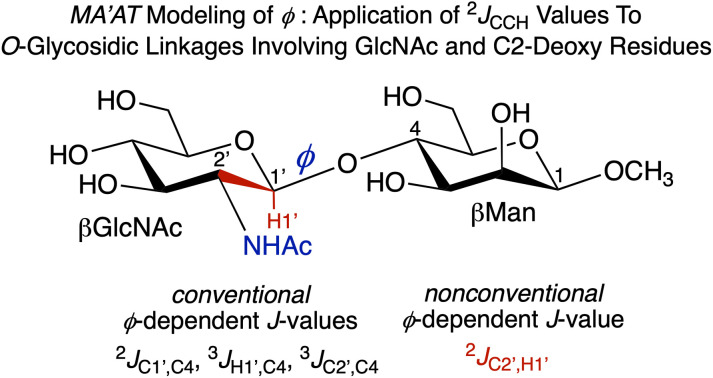

## 1 Introduction

**Scheme 1:**
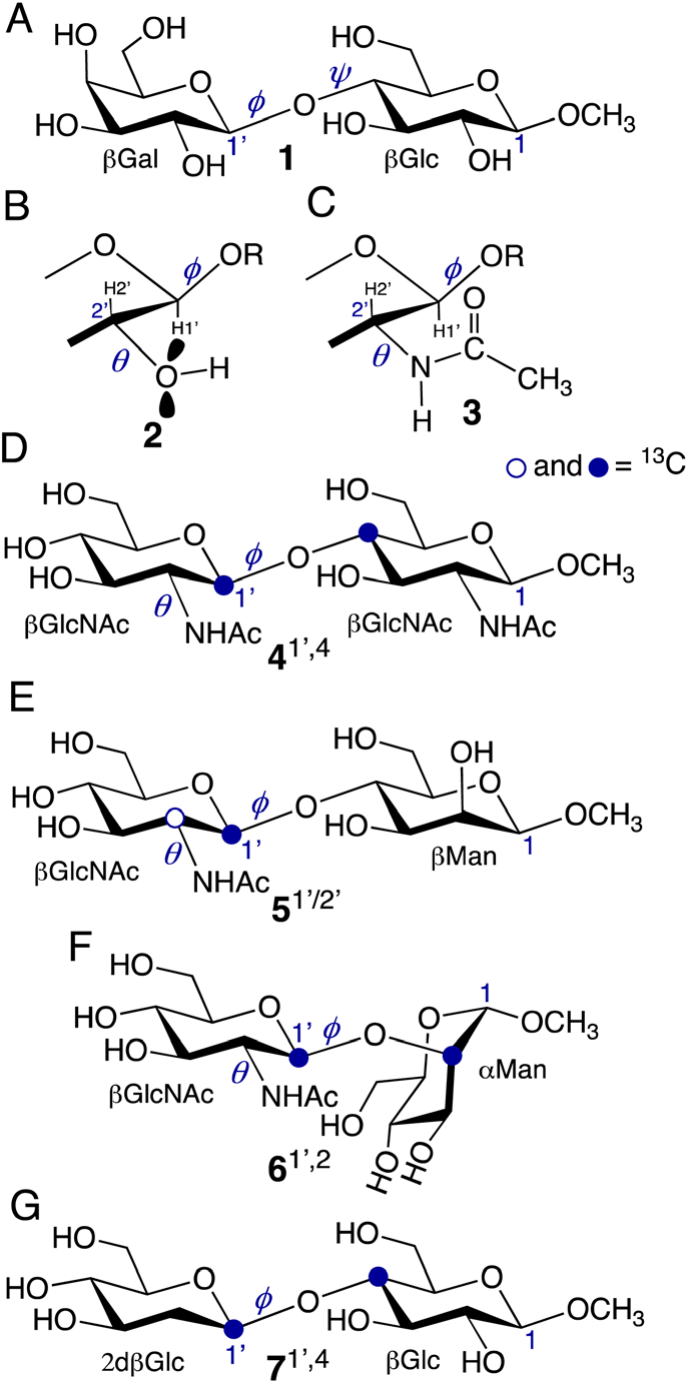
(A) Structure of methyl β-d-galactopyranosyl-(1→4)-β-d-glucopyranoside (methyl β-lactoside (1)), showing the *O*-glycosidic linkage torsion angles, *ϕ* and *ψ*. (B) Expansion of the βGal ring of 1 showing *ϕ* and *θ*, and the lone-pair orbitals on O2 whose orientation relative to the C2′–C1′–H1′ coupling pathway affects ^2^*J*_C2′,H1′_. (C) The same structure as in (B) but replacing the hydroxyl group at C2 with an *N*-acetyl side-chain. Torsion angle *θ* is more constrained in 3 than in 2, with the H2′–C2′–N2′–H torsion angle approximating 180° based on *MA’AT* analysis.^3,30^ (D)–(G) Structures of methyl 2-acetamido-2-deoxy-β-d-[1-^13^C]glucopyranosyl-(1→4)-2-acetamido-2-deoxy-β-d-[4-^13^C]glucopyranoside (4^1′,4^), methyl 2-acetamido-2-deoxy-β-d-[1-^13^C] and [2-^13^C]glucopyranosyl-(1→4)-β-d-mannopyranoside (5^1′/2′^), methyl 2-acetamido-2-deoxy-β-d-[1-^13^C]glucopyranosyl-(1→2)-α-d-[2-^13^C]mannopyranoside (6^1′,2^), and methyl 2-deoxy-β-d-[1-^13^C]arabino-texopyranosyl-(1→4)-β-d-[4-^13^C]glucopyranoside (7^1′,4^), showing the *θ* and *ϕ* torsion angles. In 1 and 4–7, anomeric carbons are labeled as either 1 or 1′. Superscripts on the compound numbers identify the carbons labeled with ^13^C.

Oligosaccharide linkage conformation has been investigated recently using a new NMR method, *MA’AT* analysis, that provides mean values of linkage torsion angles and information on their librational properties in solution.^1–3^ For linkages involving two C–O bonds, two torsion angles define linkage conformation, denoted *phi* (*ϕ*) and *psi* (*ψ*) (see methyl β-lactoside (1), Scheme 1A). The behaviors of these angles in solution are typically evaluated by NMR spectroscopy using inter-residue ^3^*J*_COCH_ and ^3^*J*_CCOC_ scalar coupling constants,^4,5^ inter-residue ^1^H–^1^H NOEs/ROEs (steady-state and transient),^6–8^ residual dipolar couplings^9,10^ and/or nuclear spin-relaxation.^11^ However, until the development of *MA’AT* analysis, experimental NMR parameters alone could not provide probability distributions of *ϕ* and *ψ* comparable to those determined by molecular dynamics (MD) simulation. Consequently, the resulting conformational assignments have been biased strongly by the MD models. *MA’AT* analysis provides experiment-based conformational models that rival those obtained by MD simulation, thus reducing or eliminating the dominant contribution that MD has made to solution models of linkage conformation.

Over the past few years, we have reported very good agreement between MD and *MA’AT* analysis with regard to mean values of *psi* (*ψ*) and their circular standard deviations (CSDs) for a range of *O*-glycosidic linkages, with the CSDs revealing the librational behavior of the angle in solution.^1–3^ In contrast, while mean values for *phi* (*ϕ*) determined by *MA’AT* analysis were in good agreement with those obtained by MD simulation, *MA’AT* analysis has consistently produced significantly larger CSDs than MD, implying greater librational motion in solution. In an effort to reconcile these differences, *phi*-dependent *J*-coupling equations were parameterized that take into account secondary effects from *psi* by constraining the parameterization to the preferred *psi* values determined by *MA’AT* analysis (*psi*-constrained *phi*-dependent equations).^12^ Use of the latter equations had little effect on *MA’AT*-determined mean values and CSDs of *phi*, leading to the conclusion that secondary effects from *psi* on equation parameterization are not responsible for the CSD discrepancy. We also performed MD simulations using both the GLYCAM06 and CHARMM force fields and compared mean values of *phi* and their CSDs obtained from both methods. Both force fields gave similar models of *ϕ*, suggesting that they are not at fault.

Recent work has brought to light the opportunity of using non-conventional *J*-couplings in *MA’AT* analyses of *phi* (*ϕ*) and *psi* (*ψ*).^13^ A potential nonconventional constraint for *ϕ* is ^2^*J*_C2′,H1′_ (ref. 14) (Scheme 1B and C). As described previously, ^2^*J*_CCH_ values involving vicinal diol (HO–C–C–OH) fragments in saccharides are particularly sensitive to rotation about the C–O bond involving the carbon bearing the coupled hydrogen (*e.g.*, ^2^*J*_C2′,H1′_ is very sensitive to rotation about *ϕ* in an *O*-glycosidic linkage such as that in 1; the primary effect).^14^ However, its use as a *phi* constraint is complicated by the fact that rotation about the C–O bond involving the coupled carbon also contributes to the ^2^*J*_CCH_ value (a secondary effect), although more modestly^14^ (*e.g.*, rotation about the C2′–O2′ bond affects ^2^*J*_C2′,H1′_ in 1; Scheme 1A and 1B). The major impediment to using ^2^*J*_C2′,H1′_ values for *ϕ* modeling by *MA’AT* analysis is uncertainty about the conformational properties of the C2′–O2′ bond bearing the coupled C2′ carbon, which leads to unacceptable uncertainties in equation parameterization.

The above-noted problem with C2′–O2′ secondary effects complicating the use of ^2^*J*_C2′,H1′_ values as *phi* constraints in *MA’AT* analysis is eliminated when the hydroxyl group at C2′ is replaced by a *N*-acetyl side-chain or by a hydrogen atom (deoxygenation). For most, although probably not all, *N*-acetyl side-chains, conformation about the C2′–N2′ bond is highly conserved to the geometry shown in Scheme 1C, as shown by recent *MA’AT* analyses of these side-chains in mono- and disaccharides.^15^ This being the case, the secondary effects of C2′–N2′ bond rotation on the parameterization of equations relating ^2^*J*_C2′,H1′_ to *ϕ* can be accurately accounted for, thereby enabling its use in *MA’AT* analysis. We describe in this report the first application of ^2^*J*_C2′,H1′_ to *MA’AT* analyses of *ϕ* in three ^13^C-labeled βGlcNAc-containing disaccharides, β-[1-^13^C]GlcNAc-(1→4)-β-[4-^13^C]GlcNAcOCH_3_ (4^1′,4^), β-[1,2-^13^C_2_]GlcNAc-(1→4)-βManOCH_3_ (5^1′,2′^), and β-[1-^13^C]GlcNAc-(1→2)-α-[2-^13^C]ManOCH_3_ (6^1′,2^), and in a deoxy disaccharide, 2dβ-[1-^13^C]Glc-(1→4)-β-[4-^13^C]GlcOCH_3_ (7^1′,4^) (superscripts on compound numbers denote the ^13^C-labeled carbons) in which secondary contributions to ^2^*J*_C2′,H1′_ are eliminated (Scheme 1D–G). We show that the inclusion of ^2^*J*_C2′,H1′_ values significantly improves the agreement between *MA’AT*-determined CSDs of *ϕ* and corresponding values obtained by MD simulation. We argue that the poor agreement between *MA’AT*- and MD-derived CSDs of *ϕ* that has been documented previously^1–3^ is caused by the particular properties of geminal ^2^*J*_COC_ values used in conventional *MA’AT* analyses of *ϕ*.

## 2 Methods

### 2.1 Experimental

#### 2.1.1 Synthesis of singly and doubly ^13^C-labeled disaccharides

The synthetic protocols used to prepare the ^13^C-labeled disaccharides 4^1′,4^, 5^1′/2′^, 6^1′,2^ and 7^1′,4^ are provided in the ESI.†

#### 2.1.2 Experimental measurements of NMR spin-coupling constants

High-resolution 1D ^1^H and ^13^C{^1^H} NMR spectra were obtained using 5-mm NMR tubes on a 600-MHz FT-NMR spectrometer equipped with a 5-mm ^1^H–^19^F/^15^N–^31^P AutoX dual broadband probe or on an 800-MHz FT-NMR spectrometer equipped with a 5-mm inverse triple resonance (TCI) ^1^H/^13^C/^15^N probe. 1D NMR spectra of unlabeled and ^13^C-labeled disaccharides were obtained in ^2^H_2_O at 22 °C and FIDs were processed to optimize both resolution and spectral S/N. ^1^H NMR spectra had digital resolutions of ∼0.02 Hz per pt and ^13^C{^1^H} NMR spectra had digital resolutions of ∼0.05 Hz per pt. ^1^H and ^13^C chemical shifts (in ppm) were referenced externally to sodium 4,4-dimethyl-4-silapentane-1-sulfonate (DSS). 2D ^1^H–^1^H gCOSY^16^ and ^13^C–^1^H gHSQC^17^ spectra were used to confirm the ^1^H and ^13^C chemical shift assignments.

Long-range ^*n*^*J*_CH_ couplings across *O*-glycosidic linkages in unlabeled compounds were obtained from 2D ^13^C–^1^H *J*-HMBC spectra^18^ using scaling factors of 10–50 and a two-fold low-pass *J*-filter to suppress ^1^*J*_CH_ values. Trans-*O*-glycosidic ^3^*J*_COCH_ values were obtained from 2D NMR spectra of unlabeled compounds when signal overlap prevented ^3^*J*_COCH_ measurement directly from signal splittings in 1D ^1^H spectra of ^13^C-labeled compounds. For measurements of trans-*O*-glycosidic ^3^*J*_COCH_ values from 1D ^1^H NMR spectra, attention was paid to potential non-first-order effects that might affect their determination directly from signal splittings. If necessary, ^1^H NMR spectra were collected at different spectrometer frequencies (500–800 MHz) and/or spectral simulation (Daisy in Bruker TopSpin 3.6.4) was used to obtain accurate ^3^*J*_COCH_ values.

Non-first-order effects were minimal in 1D ^13^C{^1^H} NMR spectra of ^13^C-labeled compounds, allowing trans-*O*-glycosidic ^2^*J*_COC_ and ^3^*J*_COCC_ values to be measured directly from the observed signal splittings.

### 2.2 Calculations

#### 2.2.1 Geometric optimization of model compounds

Fully substituted *in silico* structures 4^c^–7^c^ (the superscript “c” denotes an *in silico* structure) were used in theoretical calculations of NMR *J*-couplings. Density functional theory (DFT) calculations were conducted within *Gaussian*16 (ref. 19) using the B3LYP functional^20,21^ and 6-31G* basis set^22^ for geometric optimization. Initial exocyclic torsion angle constraints in 4^c^–7^c^ were set as shown in Schemes S1–S4 (ESI).† The internal *O*-glycosidic linkages in 4^c^–7^c^ are characterized by two torsion angles, *ϕ* and *ψ*, and each was rotated systematically in 15° increments through 360° to give 24 × 24 matrices of optimized structures (576 total structures). All remaining geometric parameters were either fixed or optimized as indicated in the Schemes S1–S4 (ESI).† The calculations included the effects of solvent water, which were treated using the Self-Consistent Reaction Field (SCRF)^23^ and the Integral Equation Formalism (polarizable continuum) model (IEFPCM)^24^ as implemented in *Gaussian*16.

#### 2.2.2 Theoretical calculations of ^1^H–^1^H, ^13^C–^1^H and ^13^C–^13^C spin-coupling constants

*J*_HH_, *J*_CH_ and *J*_CC_ values were calculated in 4^c^–7^c^ using *Gaussian*16 (ref. 19) and DFT (B3LYP functional).^20,21^ The Fermi contact,^25–27^ diamagnetic and paramagnetic spin–orbit, and spin-dipole terms^25^ were recovered using a [5s2p1d|3s1p] basis set,^28,29^ and raw (unscaled) calculated *J*-couplings are reported. The DFT calculations of *J*-couplings included the effects of solvent water, which were treated using the Self-Consistent Reaction Field (SCRF)^23^ and the Integral Equation Formalism (polarizable continuum) model (IEFPCM)^24^ as implemented in *Gaussian*16.

#### 2.2.3 Parameterization of spin-coupling equations

All geometrically-optimized conformers of 4^c^–7^c^ were inspected to ensure that structurally distorted structures were not used in equation parameterization, including the use of a 10 kcal mol^−1^ energy cutoff as described previously.^1,2^ The resulting equations (trimmed equations) relating DFT-calculated *J*-couplings to *ϕ* were parameterized using R. Equations for *ϕ* were re-parameterized using a subset of the data wherein only *ψ* conformations indicated by both *MA’AT* analysis and MD simulation were used in the parameterization (constrained equations).^12^ Disaccharides 4^c^, 5^c^ and 7^c^ generated similar models of *ψ* with an average mean value of ∼−13° and an average CSD of ∼18°. For disaccharides 4^c^ and 7^c^, *ψ* was restricted to values of −30° to +30°. For disaccharide 5^c^, *ψ* was restricted to values of −45° to +45°. The larger range of *ψ* values for 5^c^ was applied to increase the number of data points for equation parameterization since numerous structures had to be removed because of aberrant ring puckering and/or ring inversion that occurred during geometry optimization. *MA’AT* analysis of *ψ* for disaccharide 6^c^ gave a population distribution of *ψ* with a mean of 27° and CSD of 24°. For 6^c^, *ψ* was restricted to values of 0° to +60°. The goodness-of-fit of each parameterized equation is reported as a root mean squared deviation (RMSD).

#### 2.2.4 *MA’AT* analysis

*O*-Glycosidic linkage torsion angles *ϕ* in 4–7 were modeled as von Mises single-state distributions using an in-house statistical software package, *MA’AT*.^3,30^ The current version of *MA’AT* can be accessed online (https://rmeredit.shinyapps.io/maat24/) (last accessed 08/19/24) and a User's Manual is available on the software's webpage. Two sets of parameterized equations were used to model *ϕ* in 4–7 (trimmed and constrained equations as described above). Monte Carlo methods were used to generate model parameters, and least squares methods were used to minimize the RMSD between the experimental and calculated *J*-couplings. Each *MA’AT* analysis gave two fitting parameters, the mean position and the circular standard deviation (CSD) of the angle.

#### 2.2.5 Aqueous molecular dynamics simulations

Initial structures of 4^c^–7^c^ were built using the Carbohydrate Builder module available at the GLYCAM website (http://www.glycam.org).^31^ The GLYCAM06 (ref. 32) (version j) force field was employed in all simulations. The disaccharides were solvated with TIP3P^33^ water using a 12 Å buffer in a cubic box, using the LEaP module in the AMBER14 software package.^34^ Energy minimizations for the solvated disaccharides were performed separately under constant volume (500 steps steepest descent, followed by 24 500 steps of conjugate-gradient minimization). Each system was subsequently heated to 300 K over a period of 50 ps, followed by equilibration at 300 K for a further 0.5 ns using the nPT condition, with the Berendsen thermostat^35^ for temperature control. All covalent bonds involving hydrogen atoms were constrained using the SHAKE algorithm,^36^ allowing a simulation time step of 2 fs throughout the simulation. After equilibration, production simulations were carried out with the GPU implementation^37^ of the PMEMD.MPI module, and trajectory frames were collected every 1 ps for a total of 1 μs. One to four non-bonded interactions were not scaled,^38^ and a non-bonded cut-off of 8 Å was applied to van der Waals interactions, with long-range electrostatics treated with the particle mesh Ewald approximation. The output of each MD simulation was imported into *Prism*^39^ for visualization.

## 3 Results and discussion

### 3.1 Structural dependencies of *ϕ*-dependent *J*-couplings in 4–7

**Scheme 2:**
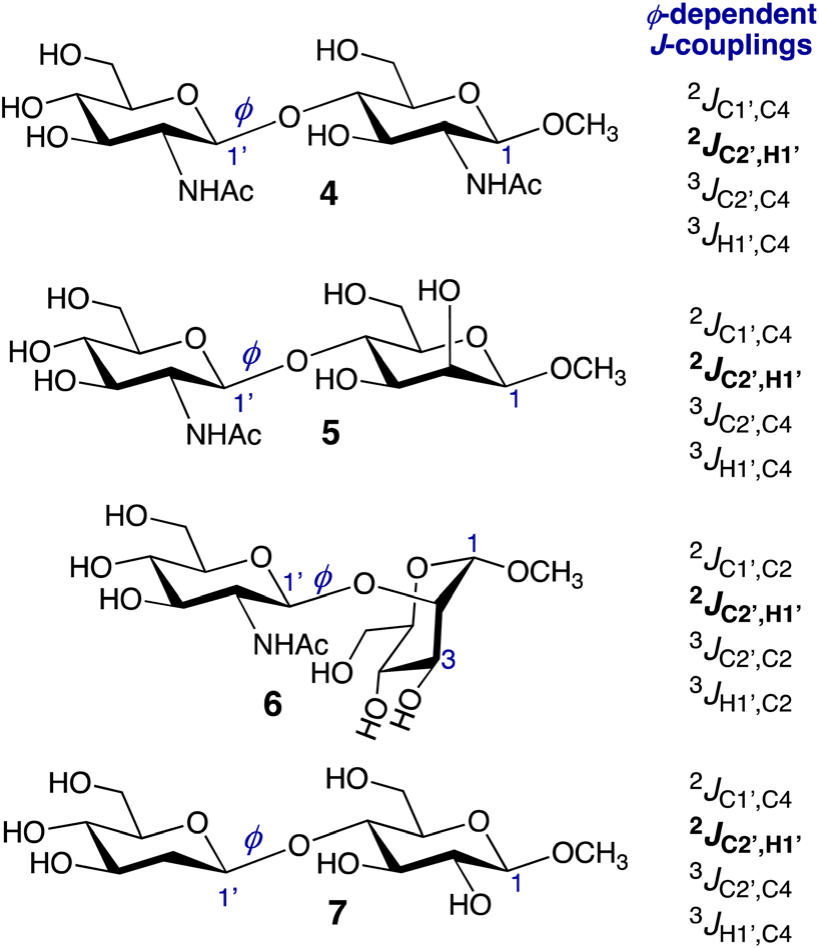
*ϕ*-Dependent conventional and nonconventional (in bold) *J*-couplings in disaccharides 4–7. In this work, the four *J*-couplings in each compound were used in three different combinations in *MA'AT* analysis of *ϕ* (Groups I–III). See Tables S1–S4 (ESI†) and the text for more discussion.

Several *J*-couplings can be used to evaluate the *O*-glycosidic linkage torsion angles, *ϕ* and *ψ*, in oligosaccharides (Scheme 1).^1–3^ For *ϕ*, prior work has focused on three trans-*O*-glycosidic *J*-couplings: a ^2^*J*_COC_, a ^3^*J*_COCH_, and a ^3^*J*_COCC_. For example, in 4, these *J*-couplings are ^2^*J*_C1′,C4_, ^3^*J*_H1′,C4_ and ^3^*J*_C2′,C2_ (Scheme 2). These *J*-values are defined as conventional *J*-couplings given their application in this manner over the past 40 years.^40^ Prior work on the development of *MA’AT* analysis to evaluate *ϕ* in *O*-glycosidic linkages have used these three conventional *J*-values. These treatments gave mean values of *ϕ* in close agreement with MD simulation but the calculated CSDs from *MA’AT* analysis have been consistently larger than those determined by MD (Fig. S5, ESI†). This discrepancy is observed regardless of the force-field used in the simulation (Fig. S5, ESI†).

**Fig. 1:**
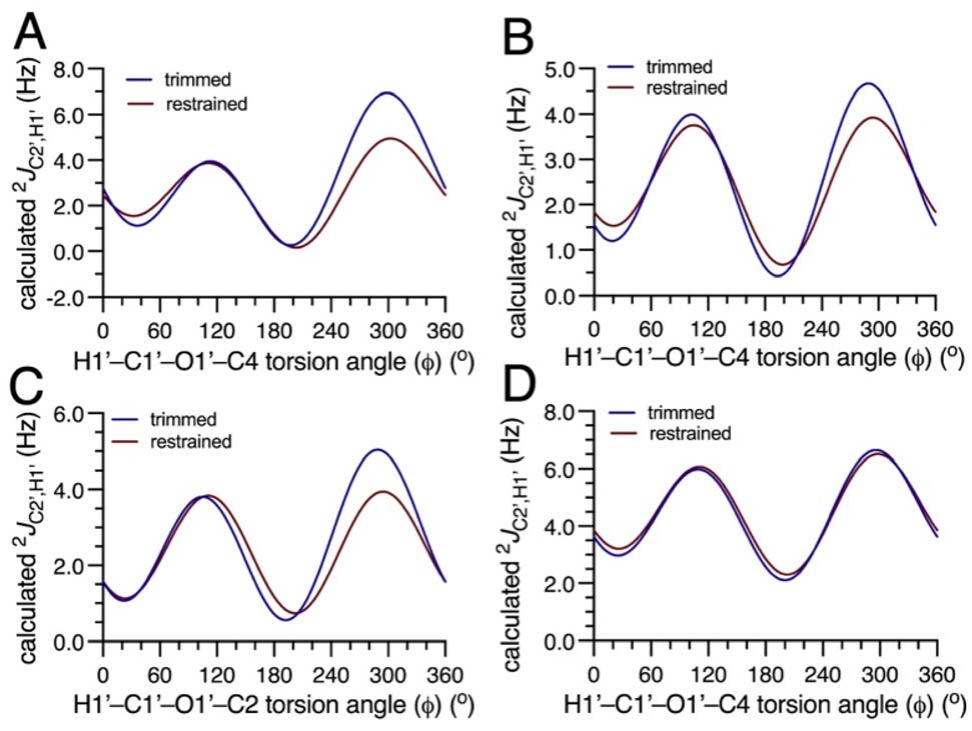
Plots showing the dependence of the calculated ^2^*J*_C2′,H1′_ value in disaccharides 4–7 on *ϕ*. (A) 4. (B) 5. (C) 6. (D) 7. For all four two-bond C2′–C1′–H1′ coupling pathways, the calculated geminal ^2^*J*_CCH_ is positive. In each plot, curves corresponding to the trimmed (blue) and constrained (red) eqn [S1]–[S32] (ESI†) are shown.

The general dependencies of the three conventional *J*-values on *ϕ* in *O*-glycosidic linkages have been reported previously,^4,5,41,42^ and are shown in Fig. S1–S4 (ESI†) for 4–7. The *ϕ*-dependencies of ^2^*J*_C2′,H1′_ are less appreciated and are shown in Fig. 1. The calculated two-bond (geminal) *J*-values have positive signs and range from ∼0 Hz to ∼+7 Hz. The overall shapes of the curves are similar (bimodal), with coupling maxima found at *ϕ* = ∼120° and ∼300°, and minima at ∼0° and ∼180°. In these calculations, the C2′–N1′ bonds in 4–6 were constrained in geometries expected in aqueous solution based on prior *MA’AT* analyses (C1′–C2′–N1′–CO torsion angle near +120°),^15^ leaving rotation about *ϕ* as the only significant determinant of ^2^*J*_C2′,H1′_. Disaccharide 7 lacks substitution at C2′, and ^2^*J*_C2′,H1′_ depends mainly on *ϕ*. In the three perfectly-stagged geometries about *ϕ* (60°, 180° and 300°), ^2^*J*_C2′,H1′_ is most positive at 300° in which one lone-pair orbital on O1′ is anti to the C1′–C2′ bond and the other is anti to the C1′–H1′ bond (assuming ideal sp^3^ character of O1′). In the three eclipsed geometries (0°, 120° and 240°), that in which two O1′ lone-pair orbitals eclipse the C1′–C2′ and C1′–H1′ bonds (120°) produces a second maximum. ^2^*J*_C2′,H1′_ values at *ϕ* values of 0°, 60°, 180°, and 240° have similar small magnitudes relative to values at 120° and 300°. The dynamic ranges of the four ^2^*J*_C2′,H1′_ values are ∼4 Hz, making them suitable for use in *MA’AT* analysis.

### 3.2 *MA’AT* analysis of *ϕ* in 4–7 using different combinations of *ϕ*-dependent *J*-couplings

**Fig. 2:**
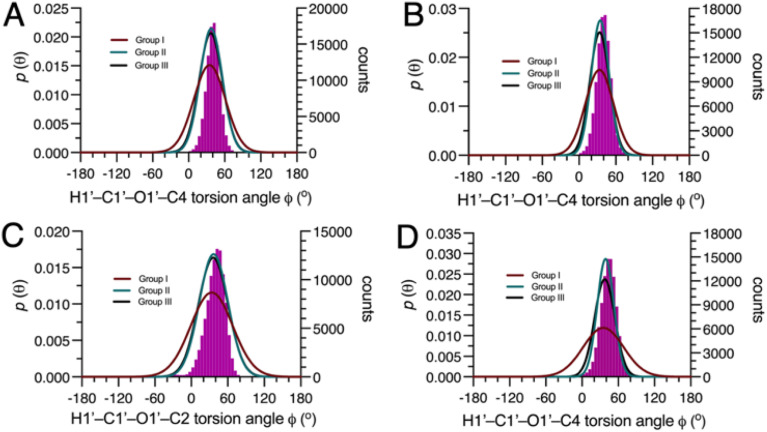
Population distributions of *ϕ* in 4–7 determined by *MA’AT* analysis using Groups I (red), II (green) and III (black) *ϕ*-dependent *J*-couplings, superimposed on the distributions determined by MD simulation (purple hatched). (A) 4. (B) 5. (C) 6. (D) 7. *MA’AT* analyses were conducted using constrained eqn [S5]–[S8], [S13]–[S16], [S21]–[S24] and [S29]–[S32] (ESI).†

Three different combinations of the *ϕ*-dependent *J*-couplings (Scheme 2) were used in *MA’AT* analyses of *ϕ* in 4–7. The *J*-couplings in each group were as follows: Group I, ^2^*J*_COC_, ^3^*J*_COCC_, ^3^*J*_COCH_; Group II, ^2^*J*_CCH_, ^3^*J*_COCC_, ^3^*J*_COCH_; Group III, ^2^*J*_COC_, ^2^*J*_CCH_, ^3^*J*_COCC_, ^3^*J*_COCH_. *MA’AT* analyses were conducted using the trimmed and constrained equations (see Calculations, Section 2.2.3) and the experimental *J*-couplings (Table 1), and the complete *MA’AT* results are provided in Tables S1–S4 (ESI).† Since the results do not depend significantly on the equations used in the analyses, only results using the constrained equations are shown in Table 2. Overlays of the *MA’AT* and MD models of *ϕ* are shown in Fig. 2 for the constrained equations; those for the trimmed equations are shown in Fig. S6 (ESI).†


*Phi*-Dependent NMR spin-coupling constants used in *MA’AT* analyses of *ϕ* in disaccharides 4–7

**Table:** 

Compound	NMR spin-coupling constant^a^			
	^2^ *J*_C1′,C2_	^2^ *J*_C2′,H1_	^3^ *J*_C2′,C2_	^3^ *J*_H1′,C2_
βGlcNAc-(1→2)-αManOCH_3_ (6)	−1.8	+0.8^b^	2.6	3.9

a In Hz + 0.1 Hz; in ^2^H_2_O at ∼22 °C.

b The sign was assumed to be positive.

c Signs were determined experimentally (see Fig. S11–S13, ESI).

**Table:** 

Compound	NMR spin-coupling constant^a^			
	^2^ *J*_C1′,C4_	^2^ *J*_C2′,H1_	^3^ *J*_C2′,C4_	^3^ *J*_H1′,C4_
βGlcNAc-(1→4)-βGlcNAcOCH_3_ (4)	−2.0	+1.4^c^	3.0	4.1
βGlcNAc-(1→4)-βManOCH_3_ (5)	−2.0	+1.0^c^	3.2	4.2
2dβGlc-(1→4)-βGlcOCH_3_ (7)	−1.9	+2.4^c^	2.9	4.0

**Table:** *MA’AT* statistics for *ϕ* in disaccharides 4–7 using different combinations of conventional and nonconventional *J*-couplings (Groups I–III)^a^ and comparison to MD

*J*-coupling group/MD	*ϕ* mean (°)	*ϕ* CSD^b^ (°)	RMSD^c^ (Hz)
**Methyl βGlcNAc-(1→4)-βGlcNAc (4)**			
Group I	34.7	26.4	0.18
Group II	37.3	18.6	0.31
Group III	36.6	19.3	0.32
MD^d^	39.5	12.8	

**Methyl βGlcNAc-(1→4)-βMan (5)**			
Group I	33.1	22.9	0.23
Group II	34.4	14.5	0.51
Group III	33.4	15.9	0.48
MD	40.2	12.1	

**Methyl βGlcNAc-(1→2)-αMan (6)**			
Group I	33.5	34.5	0.06
Group II	36.6	23.7	0.55
Group III	35.9	24.4	0.49
MD	39.7	15.0	

**Methyl 2dβGlc-(1→4)-βGlc (7)**			
Group I	35.1	33.5	0.18
Group II	38.8	13.9	0.77
Group III	37.4	16.8	0.71
MD	40.9	20.8	

a The *ϕ* sensitive *J*-couplings in Groups I–III for 4–7 are identified in Tables S1–S4, ESI. The constrained equations were used in these analyses.

b CSD = circular standard deviation.

c RMSD = room mean squared deviation.

d GLYCAM06; see text for description of MD simulations.

**Fig. 3:**
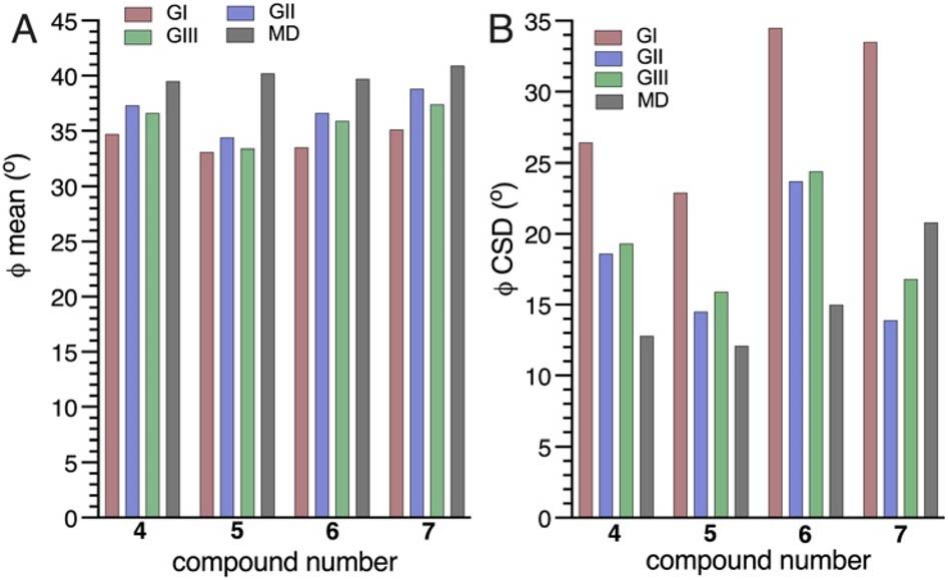
(A) *MA’AT*-determined mean values (A) and CSDs (B) for *ϕ* in 4–7 determined using Groups I (red), II (blue) and III (green) *ϕ*-dependent *J*-couplings, compared to the mean value and CSDs obtained from MD simulation (black). Constrained eqn [S5]–[S8], [S13]–[S16], [S21]–[S24] and [S29]–[S32] (ESI†) were used in the *MA’AT* analyses.

The use of different combinations of *ϕ*-dependent *J*-couplings in 4–7 has a minor effect on *MA’AT*-determined mean values, varying by at most ∼3.5° (Fig. 3A). These values are uniformly smaller than those determined by MD simulation (differences <7°). The difference is smaller when geminal ^2^*J*_C2′,H1′_ values are included in the analyses (Groups II and III).

Significant effects are observed in the CSDs of *ϕ* when ^2^*J*_C2′,H1′_ values are included in *MA’AT* analyses, with reductions of ∼9° found for 4–6 and ∼17° for 7 (Fig. 3B). The reduced CSDs are in better agreement with those obtained by MD, although in 4 and 6 the CSDs determined by MD are still ∼6° and ∼9° smaller, respectively (Table 2 and Fig. 3B). For 7, the *MA’AT*-determined CSD is smaller than that determined MD by ∼5°.

RMSDs increased by ∼0.4 Hz when ^2^*J*_C2′,H1′_ was included in *MA’AT* analyses, although their values remain relatively small, indicating good fits of the data.

The *MA’AT* and MD results, summarized in Fig. 3, show that inclusion of ^2^*J*_C2′,H1′_ in *MA’AT* analyses of *ϕ* does not affect mean values appreciably but significantly reduces CSDs, bringing them into closer, albeit not quantitative, agreement with MD. The latter finding provides new experimental evidence that geminal ^2^*J*_COC_ values are the likely cause of aberrant CSDs of *ϕ* determined by *MA’AT* analysis when conventional (Group I) *ϕ*-dependent *J*-couplings are used to model this torsion angle in *O*-glycosidic linkages.

## 4 Conclusions

In an earlier report on the use of *MA’AT* analysis to model the *phi* (*ϕ*) and *psi* (*ψ*) torsion angles of *O*-glycosidic linkages,^1^ circular standard deviations (CSDs) for *ϕ* (but not *ψ*) obtained from studies of twelve disaccharides containing β-(1→4) linkages were found to be consistently and significantly larger than those determined by MD simulation using the GYCAM06 force field. Subsequent *MA’AT* modeling of mannose-containing di- and oligosaccharides,^2^ and more recently Gal-(1→3)-Gal disaccharides,^43^ revealed similar discrepancies. Thus, taken at face value, *MA’AT* analysis was indicating greater librational averaging about the mean value of *ϕ* in aqueous solution than predicted by MD simulation. Prior work has also shown^3,12,30^ that *MA’AT* analysis gives reliable information on librational motion (dynamics) about mean molecular torsion angles, embodied in CSD values, although of the two parameters provided by the method (mean molecular torsion angles and their CSDs), CSDs are more prone to error when insufficient numbers of *J*-couplings with satisfactory dynamic ranges are available and/or when equation parameterization cannot be done reliably. In pursuit of a resolution of the CSD discrepancy, a study was undertaken^12^ wherein parameterized equations for the three conventional *J*-values used in *MA’AT* analyses of *ϕ* (^2^*J*_COC_, ^3^*J*_COCC_ and ^3^*J*_COCH_; Group I in this study) were obtained by reducing contributions made by their secondary dependencies on *ψ*, the expectation being that these secondary dependencies, which are greater for geminal ^2^*J*_COC_ values than for vicinal ^3^*J*_COCC_ and ^3^*J*_COCH_ values, were the source of the discrepancy. However, these studies revealed that CSDs for *ϕ* determined with and without *ψ* constraints on equation parameterization were nearly identical, eliminating secondary effects on the three conventional *ϕ*-dependent *J*-couplings as the cause of the discrepancy but leaving the source of the discrepancy unidentified.

Recent work^13^ aimed at improving *MA’AT* analysis of *O*-glycosidic linkages and other conformational elements in saccharides by incorporating nonconventional *J*-couplings in the analysis provided an impetus to re-examine this discrepancy from a different vantage point. Earlier work had shown^14^ that ^2^*J*_CCH_ values in saccharides are not only useful to determine pyranosyl and furanosyl ring configuration,^44–46^ but also exhibit conformational dependencies that might be exploited in *MA’AT* analysis. These nonconventional *J*-couplings include not only ^2^*J*_CCH_ values but also ^1^*J*_CH_, ^1^*J*_CC_, and ^2^*J*_CCC_ values.^13^ Impediments to their use in *MA’AT* analyses of *ϕ* and *ψ* stem from weak understandings of the conformational properties of exocyclic hydroxyl groups in aqueous solutions of saccharides which partly determine their magnitudes and sometimes their signs. Two-bond ^13^C–^1^H spin-coupling between C2 and H1 in an aldopyranosyl ring that contributes its anomeric carbon to an *O*-glycosidic linkage has particular relevance to *ϕ* modeling. The magnitude of this ^2^*J*_C2′,H1′_ value depends on rotation about the C1′–O1′ bond (*ϕ*) (primary dependence) and on rotation about the C2′–O2′ bond (secondary dependence).^14^ Even though the latter dependence is smaller than the former, it is still sufficiently strong that it cannot be ignored when parameterizing equations for use in *MA’AT* analysis. In the absence of quantitative knowledge of the conformational properties of the C2′–O2′ bond in aqueous solution, and absent the ability to capture this dependence accurately in parameterized equations, ^2^*J*_C2′,H1′_ values cannot be applied with confidence in *MA’AT* analyses of *ϕ*. We have been working on applying *MA’AT* analysis to better understand exocyclic hydroxyl group conformations in aqueous solutions of saccharides, but results are presently incomplete.

The above-noted limitation associated with the use of nonconventional ^2^*J*_C2′,H1′_ values in *MA’AT* analyses of *ϕ* is circumvented when the residue participating in the *O*-glycosidic linkage bears an *N*-acetyl functional group at C2′, or no substituent at C2′, instead of a hydroxyl group. Unlike hydroxyl groups, exocyclic *N*-acetyl groups are highly conformationally constrained in most cases, and *MA’AT* analysis has recently modeled this behavior as uni-modal using six redundant *J*-couplings sensitive to rotation about the C2′–N′ bond.^15^ In general, rotation about this bond is conserved in most C2 *N*-acetylated aldopyranosyl rings, as demonstrated by MD in the βGlcNAc rings of 4–6 (Fig. S7, ESI†), allowing equation parameterization for ^2^*J*_C2′,H1′_ in which the only significant conformational dependency is on *ϕ*. In deoxy structures like 7, secondary effects from C2′–O2′ bond rotation are absent, making parameterization of ^2^*J*_C2′,H1′_ straightforward.

The results of this study confirm the value of adding a nonconventional *J*-coupling to *MA’AT* analyses of *ϕ*. While the addition of this constraint does not alter calculated mean values of *ϕ* appreciably, its inclusion results in a significant reduction in CSDs, bringing their values into closer alignment with MD simulation. As shown in the Group III calculations, even when all four redundant *J*-couplings are used, a significant reduction in the CSD is observed. For 7, complications arising from C2′–O2′ bond rotation are absent, making ^2^*J*_C2′,H1′_ a valuable constraint for *ϕ*. On the other hand, the approach described here cannot be applied at present to *O*-glycosidic linkages in which the donor residue bears an hydroxyl group at C2′. While it is anticipated that the values of *MA’AT*-determined CSDs of *ϕ* may also decline upon inclusion of ^2^*J*_C2′,H1′_ values in *MA’AT* analysis, additional work is needed to better understand the conformational behavior of C2′–O2′ bonds to enable their use to test this prediction.

**Fig. 4:**
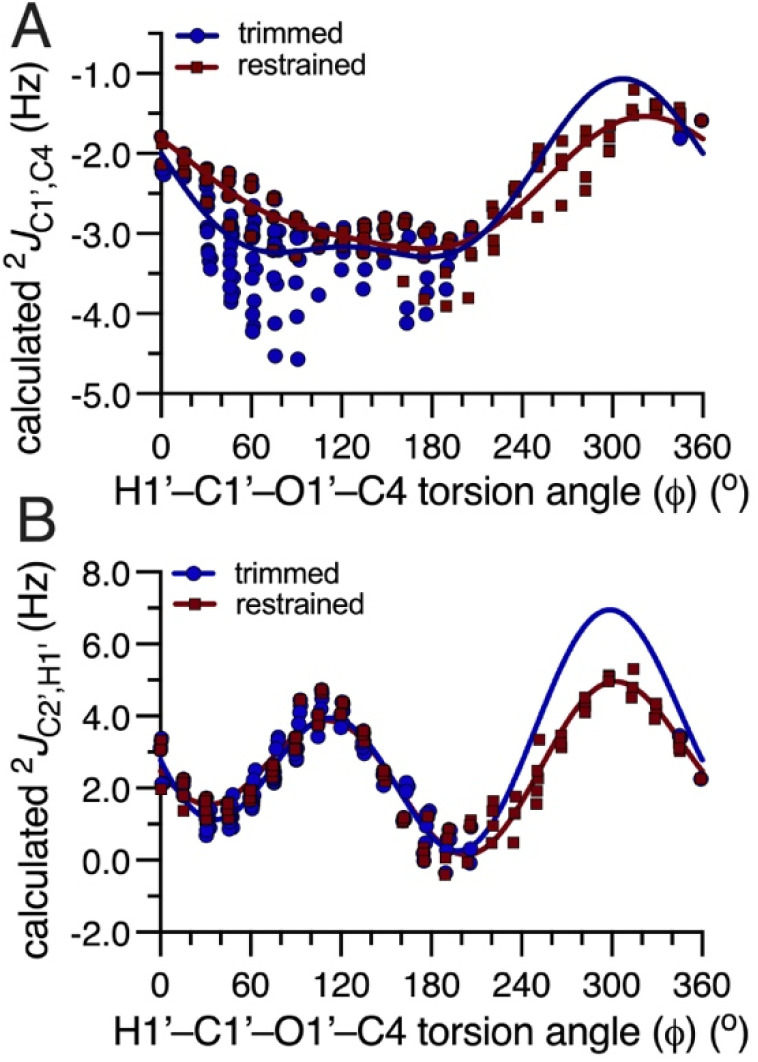
Plots of the dependencies of ^2^*J*_C1′,C4_ (A) and ^2^*J*_C2′,H1′_ (B) in 4 on the H1′–C1′–O1′–C4 torsion angle *ϕ*. Blue circles, trimmed data; red squares, constrained data. The solid lines are plots of the trimmed (blue) and constrained (red) equations for each *J*-coupling. Point scatter at discrete values of *ϕ* is caused by the secondary dependence of the *J*-coupling on *ψ*.

Of interest is why the inclusion of ^2^*J*_C2′,H1′_ values in *MA’AT* analyses of *ϕ* improves the modeling of this torsion angle, as inferred from the better agreement with MD results. An inspection of the plots in Fig. 4 for 4 provides insight into this question and reveals aspects of *MA’AT* analysis worth illuminating. Secondary effects of *ψ* on ^2^*J*_C1′,C4_ are substantial even when *ψ* constraints are applied to the dataset (constrained data), leading to inherent uncertainty in equation parameterization (Fig. 4A). In contrast, secondary affects from *ψ* are smaller for ^2^*J*_C2′,H1′_ (Fig. 4B). More importantly, the plot of the dependence of ^2^*J*_C1′,C4_ on *ϕ* contains a region between *ϕ* = 0–180° where the *J*-value is relatively constant (−2–−3°), that is, in the region that includes the *MA’AT*-determined mean value of *ϕ* for 4 (35–37°; Table 2). In contrast, the same region for ^2^*J*_C2′,H1′_ shows values ranging from ∼1–5 Hz. While the behavior of ^2^*J*_C1′,C4_ is sufficient when combined with those of the two vicinal *ϕ*-dependent *J*-couplings (^3^*J*_C2′,C4_ and ^3^*J*_H1′,C4_) to compute a reproducible mean value of *ϕ*, the flat region of the ^2^*J*_C1′,C4_ curve renders it less discriminating than that for ^2^*J*_C2′,H1′_, leading to an artificially broadened population distribution for *ϕ*. This fact emphasizes the importance of not only the dynamic range of the *J*-coupling across the full 360° torsion angle itinerary, but also the shape of the dependency in the region where the mean value of the molecular torsion angle resides. The sinusoidal behavior of ^2^*J*_C2′,H1′_ and its more reliable parameterization due in part to smaller secondary effects from *ψ* increase its discriminatory power in *MA’AT* analysis relative to that of ^2^*J*_C1′,C4_.

Prior reports have noted that the width of the population distribution of a molecular torsion angle determined by *MA’AT* analysis will be affected by the dynamic ranges of the redundant *J*-couplings, the nature (shape) of their dependencies on the torsion angle, the quality of the parameterized equations used to fit the *J*-couplings, and the accuracy of the experimental *J*-couplings.^3,30^ Errors in one or more of these contributing factors will increase the width of the distribution (*i.e.*, increase the calculated CSD). In this regard the population distribution can be compared to peak width in NMR, wherein linewidth in the absence of chemical exchange is determined by the intrinsic *T*_2_ of the nucleus (the true linewidth) and by field inhomogeneity 
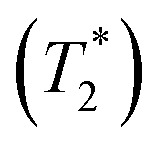
. A CSD value determined by *MA’AT* analysis is expected to always be equal to or greater than the true CSD (*i.e.*, the *MA’AT*-determined CSDs are upper limits). This being the case, the smaller *MA’AT*-determined CSD found for *ϕ* in 7 (Groups II and III, Table 2) compared to that determined by MD suggests that the latter may be incorrect, that is, librational averaging about *ϕ* in 7 is probably smaller than that indicated by MD simulation.

## Data availability

The data supporting this article have been included as part of the ESI.† The current version of the *MA’AT* application can be accessed online (https://rmeredit.shinyapps.io/maat24/) (last accessed: 08/21/2024) and a User's Manual is available on the software's webpage.

## Conflicts of interest

There are no conflicts of interest to declare.

## Supplementary Material

RA-014-D4RA06062H-s001
