# Supplementary material for: MA’AT analysis of the O-glycosidic linkages of oligosaccharides using nonconventional NMR J-couplings: MA’AT and MD models of phi
Source: RSC Adv. 2024 Sep 23;14(41):30286–94. doi: 10.1039/d4ra06062h (PMC11418834; doi:10.1039/d4ra06062h)
Supplement: RA-014-D4RA06062H-s001 [file RA-014-D4RA06062H-s001.pdf]

Supplementary Information (SI) for RSC Advances.
This journal is © The Royal Society of Chemistry 2024

Supporting Information

MA'AT Analysis of the *O*-Glycosidic Linkages of Oligosaccharides Using Nonconventional NMR *J*-Couplings: *MA'AT* and MD Models of *Phi*

Reagan J. Meredith,[1,2] Wenhui Zhang,[3] Mi-Kyung Yoon,[1,3] Xiaosong Hu,[4] Ian Carmichael[5] and Anthony S. Serianni[1]*

[1]Department of Chemistry and Biochemistry, University of Notre Dame, Notre Dame, IN 46556 USA; [2]Texas Biomedical Research Institute, San Antonio, TX 78227 USA; [3]Omicron Biochemicals, Inc., South Bend, IN 46617 USA; [4]Department of Chemistry, Wuhan University of Technology, Wuhan 430070, China; [5]Radiation Laboratory, University of Notre Dame, Notre Dame, IN 46556 USA

Author for correspondence: aseriann@nd.edu

Table of Contents

1. Table S1. *MA'AT* statistics for ϕ in **4** using different combinations of *J*-couplings S3
2. Table S2. *MA'AT* statistics for ϕ in **5** using different combinations of *J*-couplings S4
3. Table S3. *MA'AT* statistics for ϕ in **6** using different combinations of *J*-couplings S5
4. Table S4. *MA'AT* statistics for ϕ in **7** using different combinations of *J*-couplings S6
5. Figure S1. Plots of DFT-calculated ϕ-dependent *J*-couplings in **4** as a function of ϕ S7
6. Figure S2. Plots of DFT-calculated ϕ-dependent *J*-couplings in **5** as a function of ϕ S8
7. Figure S3. Plots of DFT-calculated ϕ-dependent *J*-couplings in **6** as a function of ϕ S9
8. Figure S4. Plots of DFT-calculated ϕ-dependent *J*-couplings in **7** as a function of ϕ S10
9. Figure S5. Comparison of MD histograms of ϕ and ψ in methyl β-lactoside obtained from aqueous 1-μs simulations using GLYCAM06 and CHARMM S11
10. Figure S6. Population distributions of ϕ in **4–7** determined by *MA'AT* analysis (Groups I–III) superimposed on distributions obtained from MD S12
11. Figure S7. MD histograms of torsion angles in the *N*-acetyl side-chains of **4–6** S13
12. Scheme S1. Torsion angle constraints in DFT calculations of **4**[c] S14
13. Scheme S2. Torsion angle constraints in DFT calculations of **5**[c] S15
14. Scheme S3. Torsion angle constraints in DFT calculations of **6**[c] S16
15. Scheme S4. Torsion angle constraints in DFT calculations of **7**[c] S17
16. DFT-parameterized spin–coupling equations (trimmed and constrained) for **4–7** S18
17. Figure S8. Partial 1D $^{13}C\{^1H\}$ NMR spectrum of **5**[1'] S21
18. Figure S9. Partial 1D $^{13}C\{^1H\}$ NMR spectrum of **5**[2'] S22
19. Figure S10. Partial 2D J-HMBC spectrum of **5** in ($^3J_{C4,H1'}$ determination) S23
20. Figure S11. Partial 2D HSQC-HECADE spectrum of **5** ($^2J_{C2',H1'}$ determination) S24
21. Figure S12. Partial 2D HSQC-HECADE spectrum of **7** ($^2J_{C2',H1'}$ determination) S25
22. Figure S13. Partial 2D HSQC-HECADE spectrum of **4** ($^2J_{C2',H1'}$ determination) S26

23. Preparation of disaccharide **4**$^{1',4}$ S27
24. Preparation of disaccharides **5**$^{1'}$ and **5**$^{2'}$ S35
25. Preparation of disaccharide **6**$^{1',2}$ S50
26. Preparation of disaccharide **7**$^{1',4}$ S56
27. Representative Cartesian coordinates for DFT structures **4**^{c}–**7**^{c} S73
28. Brief discussion of the functional and basis sets used in DFT calculations S78
28. Complete references 19 and 34 S79

Table S1. *MA'AT* Statistics for ϕ in Methyl βGlcNAc-(1→4)-βGlcNAc (**4**) Using Different Combinations of Conventional and Nonconventional *J*-Coupling

***J*-coupling groups**	***J*-couplings used in *MA'AT* fits**			
Group I	$^{2}J_{C1',C4}$		$^{3}J_{C2',C4}$	$^{3}J_{H1',C4}$
Group II		$^{2}J_{C2',H1'}$	$^{3}J_{C2',C4}$	$^{3}J_{H1',C4}$
Group III	$^{2}J_{C1',C4}$	$^{2}J_{C2',H1'}$	$^{3}J_{C2',C4}$	$^{3}J_{H1',C4}$

***J*-coupling groups**	**ϕ mean (º)**	**ϕ CSD (º)**	**RMSD (Hz)**
	trimmed equations		
Group I	24.3	21.6	0.36
Group II	30.3	15.8	0.07
Group III	27.3	17.6	0.35
	constrained equations		
Group I	34.7	26.4	0.18
Group II	37.3	18.6	0.31
Group III	36.6	19.3	0.32

Table S2. *MA'AT* Statistics for ϕ in Methyl βGlcNAc-(1→4)-βMan (**5**) Using Different Combinations of Conventional and Nonconventional *J*-Couplings

***J*-coupling groups**	***J*-couplings used in *MA'AT* fits**			
Group I	$^{2}J_{C1',C4}$		$^{3}J_{C2',C4}$	$^{3}J_{H1',C4}$
Group II		$^{2}J_{C2',H1'}$	$^{3}J_{C2',C4}$	$^{3}J_{H1',C4}$
Group III	$^{2}J_{C1',C4}$	$^{2}J_{C2',H1'}$	$^{3}J_{C2',C4}$	$^{3}J_{H1',C4}$

***J*-coupling groups**	**ϕ mean (°)**	**ϕ CSD (°)**	**RMSD (Hz)**
	trimmed equations		
Group I	28.2	16.2	0.40
Group II	30.2	7.8	0.25
Group III	28.7	10.5	0.41
	constrained equations		
Group I	33.1	22.9	0.23
Group II	34.4	14.5	0.51
Group III	33.4	15.9	0.48

Table S3. *MA'AT* Statistics for ϕ in Methyl βGlcNAc-(1→2)-αMan (**6**) Using Different Combinations of Conventional and Nonconventional *J*-Couplings

***J*-coupling groups**	***J*-couplings used in *MA'AT* analysis**			
Group I	$^2J_{C1',C2}$		$^3J_{C2',C2}$	$^3J_{H1',C2}$
Group II		$^2J_{C2',H1'}$	$^3J_{C2',C2}$	$^3J_{H1',C2}$
Group III	$^2J_{C1',C2}$	$^2J_{C2',H1'}$	$^3J_{C2',C2}$	$^3J_{H1',C2}$

***J*-coupling groups**	**ϕ mean (º)**	**ϕ CSD (º)**	**RMSD (Hz)**
	trimmed equations		
Group I	19.6	35.0	0.33
Group II	28.9	22.6	0.51
Group III	25.2	25.2	0.56
	constrained equations		
Group I	33.5	34.5	0.06
Group II	36.6	23.7	0.55
Group III	35.9	24.4	0.49

Table S4. *MA'AT* Statistics for ϕ in Methyl 2dβGlc-(1→4)-βGlc (**7**) Using Different Combinations of Conventional and Nonconventional *J*-Couplings

***J*-coupling groups**	***J*-couplings used in *MA'AT* fits**			
Group I	$^{2}J_{C1',C4}$		$^{3}J_{C2',C4}$	$^{3}J_{H1',C4}$
Group II		$^{2}J_{C2',H1'}$	$^{3}J_{C2',C4}$	$^{3}J_{H1',C4}$
Group III	$^{2}J_{C1',C4}$	$^{2}J_{C2',H1'}$	$^{3}J_{C2',C4}$	$^{3}J_{H1',C4}$

***J*-coupling groups**	**ϕ mean (º)**	**ϕ CSD (º)**	**RMSD (Hz)**
	trimmed equations		
Group I	25.7	27.6	0.33
Group II	32.1	9.8	0.57
Group III	29.7	14.1	0.51
	constrained equations		
Group I	35.1	33.5	0.18
Group II	38.8	13.9	0.77
Group III	37.4	16.8	0.71

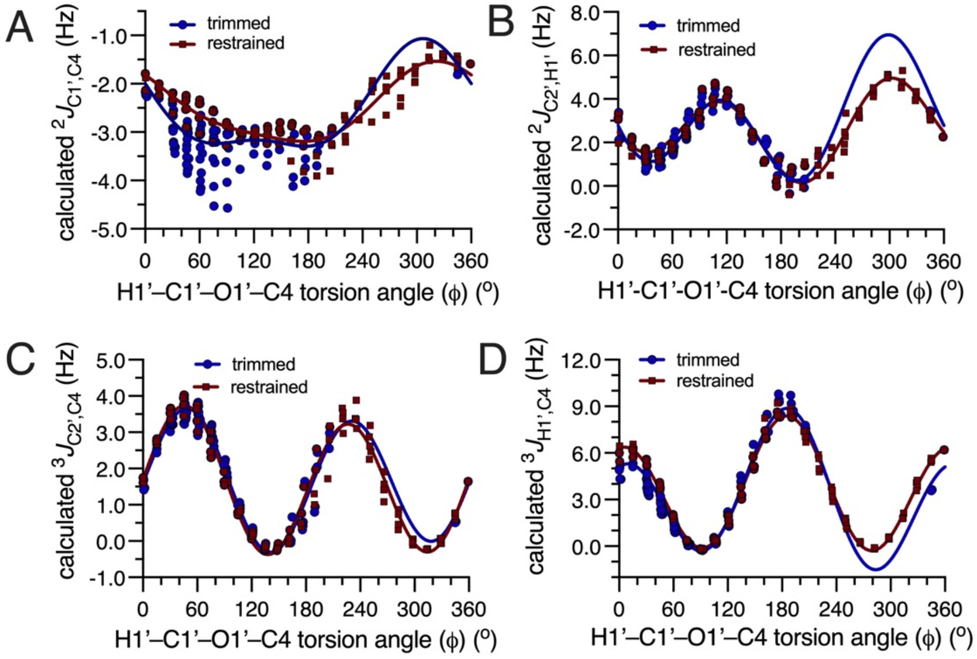

Figure S1. Plots of DFT-calculated ϕ-dependent *J*-couplings in **4** as a function of the H1'–C1'–O1'–C4 torsion angle (ϕ). (A) $^2J_{C1',C4}$. (B) $^2J_{C2',H1'}$. (C) $^3J_{C2',C4}$. (D) $^3J_{H1',C4}$. In each plot, the blue curve (trimmed equation) is the fit of the blue data points (circles) and the red curve (constrained equation) is the fit of the red data points (squares). Point scatter along the *y*-axis at discrete values of ϕ in each plot is caused by the secondary dependence of the *J*-coupling on ψ.

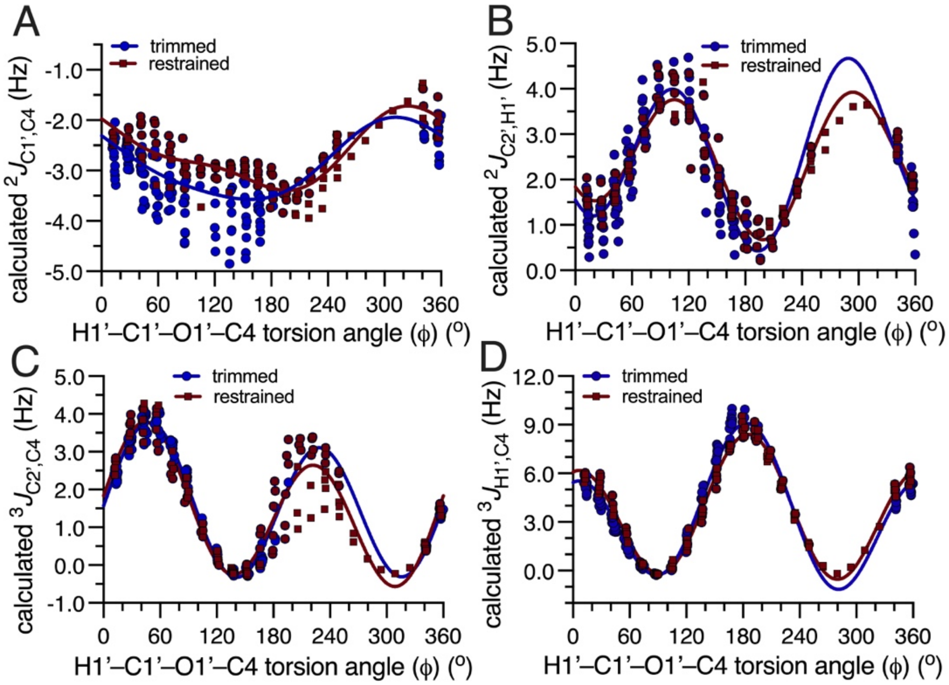

Figure S2. Plots of DFT-calculated ϕ-dependent *J*-couplings in **5** as a function of the H1'–C1'–O1'–C4 torsion angle (ϕ). (A) $^2J_{C1',C4}$. (B) $^2J_{C2',H1'}$. (C) $^3J_{C2',C4}$. (D) $^3J_{H1',C4}$. In each plot, the blue curve (trimmed equation) is the fit of the blue data points (circles) and the red curve (constrained equation) is the fit of the red data points (squares). Point scatter along the *y*-axis at discrete values of ϕ in each plot is caused by the secondary dependence of the *J*-coupling on ψ.

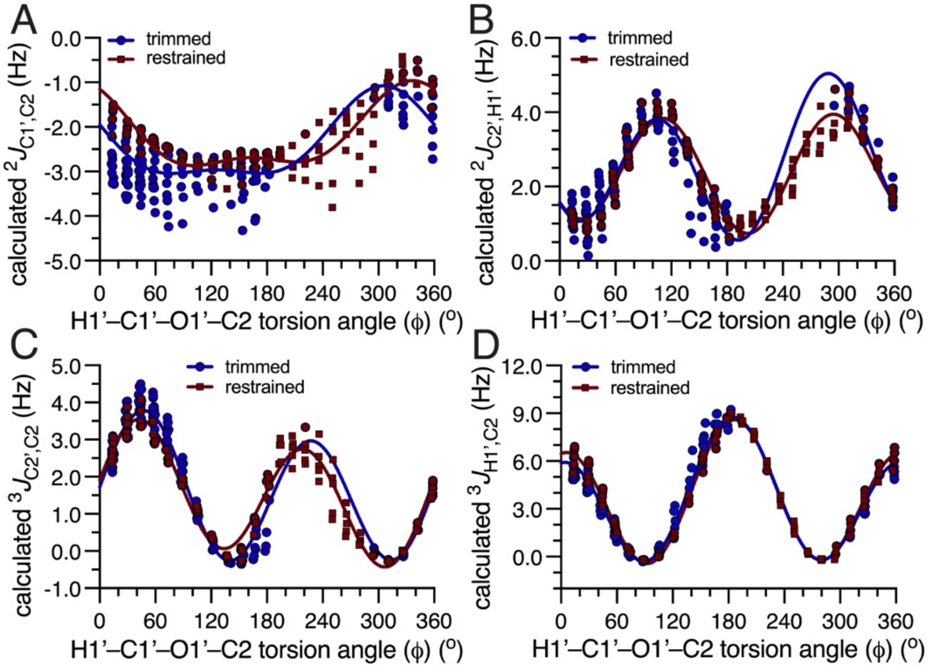

Figure S3. Plots of DFT-calculated ϕ-dependent *J*-couplings in **6** as a function of the H1'–C1'–O1'–C2 torsion angle (ϕ). (A) $^2J_{C1',C2}$. (B) $^2J_{C2',H1'}$. (C) $^3J_{C2',C2}$. (D) $^3J_{H1',C2}$. In each plot, the blue curve (trimmed equation) is the fit of the blue data points (circles) and the red curve (constrained equation) is the fit of the red data points (squares). Point scatter along the *y*-axis at discrete values of ϕ in each plot is caused by the secondary dependence of the *J*-coupling on ψ.

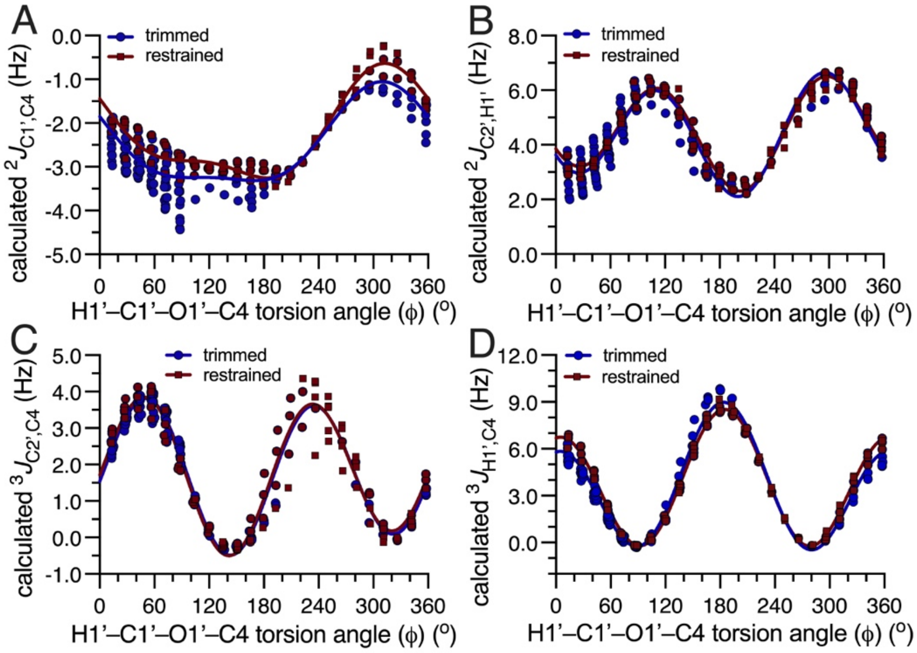

Figure S4. Plots of DFT-calculated ϕ-dependent *J*-couplings in **7** as a function of the H1'–C1'–O1'–C4 torsion angle (ϕ). (A) $^2J_{C1',C4}$. (B) $^2J_{C2',H1'}$. (C) $^3J_{C2',C4}$. (D) $^3J_{H1',C4}$. In each plot, the blue curve (trimmed equation) is the fit of the blue data points (circles) and the red curve (constrained equation) is the fit of the red data points (squares). Point scatter along the *y*-axis at discrete values of ϕ in each plot is caused by the secondary dependence of the *J*-coupling on ψ.

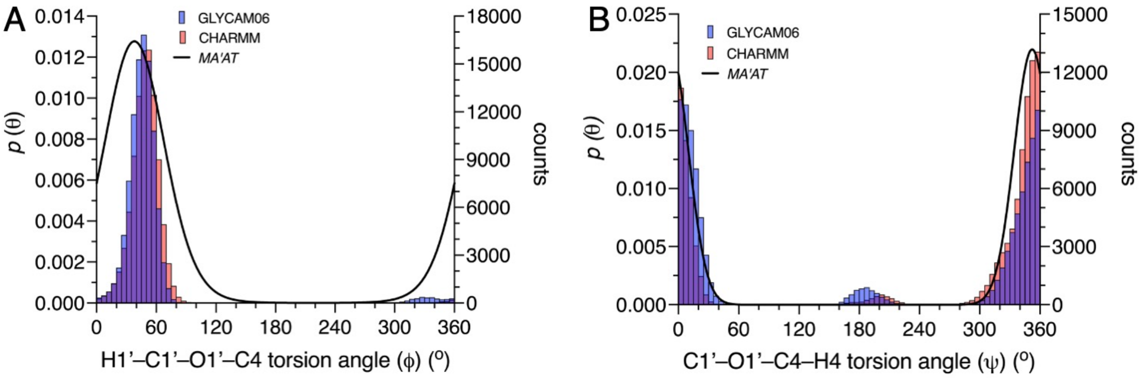

Figure S5. A comparison of MD histograms of the *O*-glycosidic torsion angles *phi* (ϕ) (A) and *psi* (ψ) (B) in methyl β-D-galactopyranosyl-(1→4)-β-D-glucopyranoside (methyl β-lactoside) obtained from aqueous 1-μs simulations using the GLYCAM06 (blue hatched) and CHARMM (orange hatched) force fields. Both MD methods give similar populations distributions. *MA'AT* analysis (solid black line) of ψ is in good agreement with MD with regard to both mean value and CSD. *MA'AT* analysis of ϕ is in good agreement with MD with regard to mean value, but the *MA'AT*-determined CSD is much larger (wider population distribution) than those determined by MD.

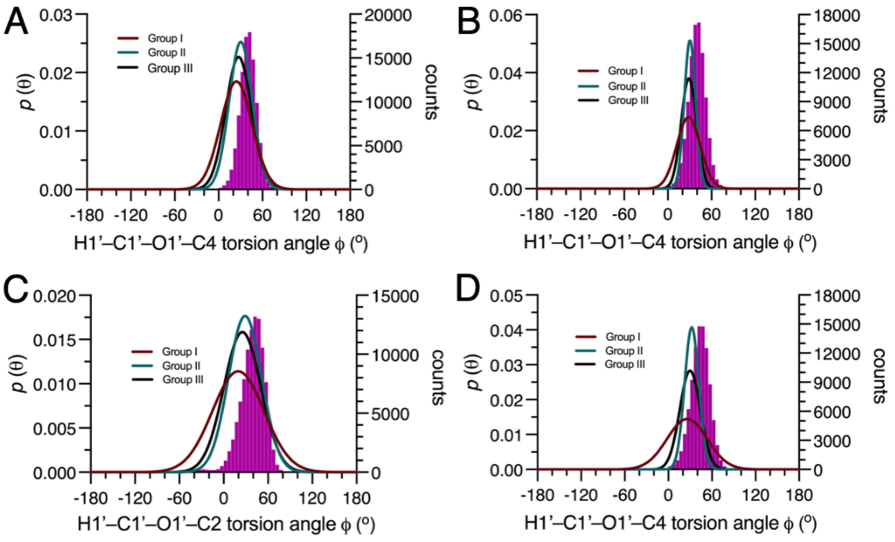

Figure S6. Population distributions of ϕ in **4**–**7** determined by *MA'AT* analysis using Group **I** (red), **II** (green) and **III** (black) ϕ-dependent *J*-couplings, superimposed on the distributions determined by MD simulation (purple hatched). (A) **4**. (B) **5**. (C) **6**. (D) **7**. *MA'AT* analyses were conducted using trimmed equations [S1]–[S4] for **4**, [S9]–[S12] for **5**, [S17]–[S20] for **6** and [S25]–[S28] for **7**.

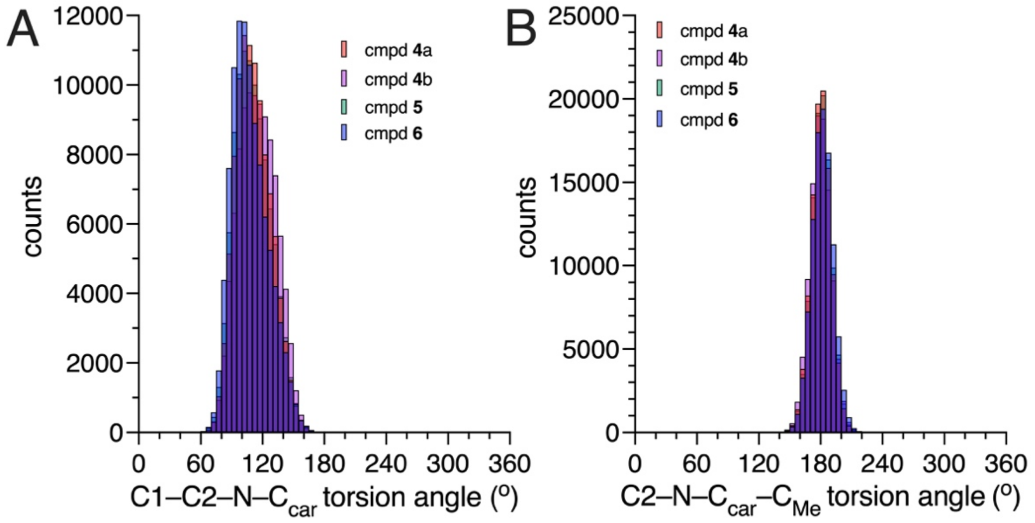

Figure S7. Aqueous MD histograms of torsion angles in the *N*-acetyl side-chains of **4**–**6**. (A) C1–C2–N–C_{car} torsion angle. (B) C2–N–C_{car}-C_{Me} torsion angle. Data show the side-chains adopt similar conformations in the four compounds. Data in (B) indicate a highly preferred *trans* configuration of the amide bonds. See text for MD details. Disaccharide **4** contains two *N*-acetyl side-chains, denoted a and b.

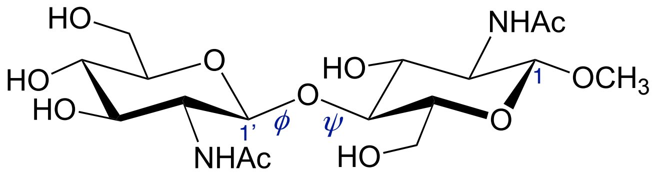

βGlcNAc-(1→4)-βGlcNAcOCH$_3$ (**4**^{c})

C2–C1–O1–CH$_3$: fixed 180°
H2–C2–N–H: fixed 180°
C2–N–C$_{car}$–C$_{Me}$: fixed 180°
C2–C3–O3–H: fixed 180°
C4–C5–C6–O6: fixed 180°
C5–C6–O6–H: fixed 180°

H2'–C2'–N'–H: fixed 180°
C2'–N'–C$_{car}$–C$_{Me}$: fixed 180°
C2'–C3'–O3'–H: fixed 180°
C3'–C4'–O4'–H: fixed 180°
C4'–C5'–C6'–O6': fixed 180°
C5'–C6'–O6'–H: fixed 180°

phi (ϕ) : O5'–C1'–O1'–C4: 15° rotations
psi (ψ): C1'–O1'–C4–C3: 15° rotations

Scheme S1. Torsion Angle Constraints Used in DFT Calculations of **4**^{c}.

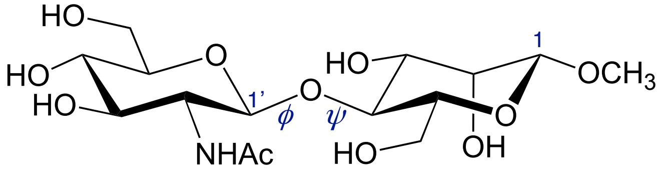

βGlcNAc-(1→4)-βManOCH$_3$ (**5**^{c})

C2–C1–O1–CH$_3$: initial, 180°
C1–C2–O2–H: initial, -40°
C2–C3–O3–H: initial, 180°
C4–C5–C6–O6: initial, 180°
C5–C6–O5–H: initial, 180°

C1'–C2'–N'–CO: initial,116°
C2'–N'–C$_{car}$–C$_{Me}$: initial, 180°
C2'–C3'–O3'–H: initial, 180°
C3'–C4'–O4'–H: initial, 180°
C4'–C5'–C6'–O6': initial, 170°
C5'–C6'–O6'–H: initial, –60°

phi (ϕ) : C2'–C1'–O1'–C4: 15° rotations
psi (ψ): C1'–O1'–C4–C3: 15° rotations

Scheme S2. Torsion Angle Constraints Used in DFT Calculations of **5**^{c}.

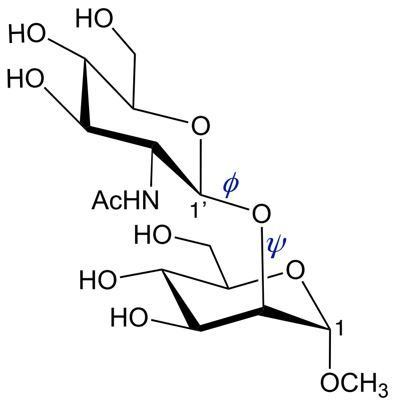

βGlcNAc-(1→2)-αManOCH$_3$ (**6**^{c})

C2–C1–O1–CH$_3$: initial, 180°
C2–C3–O3–H: initial, 180°
C3–C4–O4–H: initial, 50°
C4–C5–C6–O6: initial, 180°
C5–C6–O5–H: initial, 180°

C1'–C2'–N'–C$_{car}$: fixed, 116°
C2'–N'–C$_{car}$–C$_{Me}$: initial, 180°
C2'–C3'–O3'–H: initial, 170°
C3'–C4'–O4'–H: initial, 160°
C4'–C5'–C6'–O6': initial, 60°
C5'–C6'–O6'–H: initial, 160°

phi (ϕ) : C2'–C1'–O1'–C2: 15° rotations
psi (ψ): C1'–O1'–C2–C1: 15° rotations

Scheme S3. Torsion Angle Constraints Used in DFT Calculations of **6**^{c}.

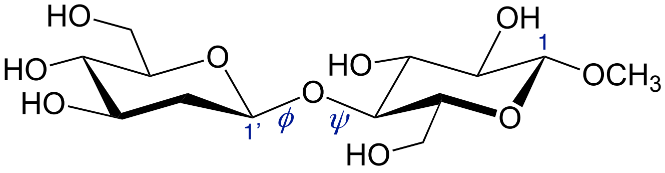

2dβGlc-(1→4)-βGlcOCH3 (**7**c)

C2–C1–O1–CH_3: initial, 180°
C1–C2–O2–H: initial, 60°
C2–C3–O3–H: initial, 180°
C4–C5–C6–O6: initial, 180°
C5–C6–O5–H: initial, 180°

C2'–C3'–O3'–H: initial, 180°
C3'–C4'–O4'–H: initial, 180°
C4'–C5'–C6'–O6': initial, 180°
C5'–C6'–O6'–H: initial, 180°

phi (ϕ) : C2'–C1'–O1'–C4: 15° rotations
psi (ψ): C1'–O1'–C4–C3: 15° rotations

Scheme S4. Torsion Angle Constraints Used in DFT Calculations of **7**c.

DFT–Parameterized Spin–Coupling Equations: Structures 4–7

Structure 4 (trimmed data set)
ϕ: H1'–C1'–O1'–C4
ψ: C1'–O1'–C4–H4

$$^{2}J_{C1',C4}\text{ (Hz)} = -2.52 + 0.65\cos\phi - 0.83\sin\phi - 0.13\cos 2\phi - 0.38\sin 2\phi \quad \text{RMSD} = 0.44\text{ Hz} \quad [S1]$$

$$^{2}J_{C2',H1'}\text{ (Hz)} = 3.12 + 1.06\cos\phi - 1.15\sin\phi - 1.40\cos 2\phi - 1.84\sin 2\phi \quad \text{RMSD} = 0.40\text{ Hz} \quad [S2]$$

$$^{3}J_{C2',C4}\text{ (Hz)} = 1.66 + 0.23\cos\phi - 0.29\cos 2\phi + 1.79\sin 2\phi \quad \text{RMSD} = 0.27\text{ Hz} \quad [S3]$$

$$^{3}J_{H1',C4}\text{ (Hz)} = 3.13 - 1.85\cos\phi + 0.38\sin\phi + 3.81\cos 2\phi + 0.98\sin 2\phi \quad \text{RMSD} = 0.49\text{ Hz} \quad [S4]$$

Structure 4 (constrained data set)
ϕ: H1'–C1'–O1'–C4
ψ: C1'–O1'–C4–H4

$$^{2}J_{C1',C4}\text{ (Hz)} = -2.49 + 0.69\cos\phi - 0.41\sin\phi - 0.17\sin 2\phi \quad \text{RMSD} = 0.25\text{ Hz} \quad [S5]$$

$$^{2}J_{C2',H1'}\text{ (Hz)} = 2.62 + 0.87\cos\phi - 0.17\sin\phi - 1.02\cos 2\phi - 1.42\sin 2\phi \quad \text{RMSD} = 0.48\text{ Hz} \quad [S6]$$

$$^{3}J_{C2',C4}\text{ (Hz)} = 1.57 + 0.20\cos\phi + 0.16\sin\phi - 0.07\cos 2\phi + 1.90\sin 2\phi \quad \text{RMSD} = 0.27\text{ Hz} \quad [S7]$$

$$^{3}J_{H1',C4}\text{ (Hz)} = 3.60 - 1.01\cos\phi + 3.71\cos 2\phi + 0.71\sin 2\phi \quad \text{RMSD} = 0.32\text{ Hz} \quad [S8]$$

****

Structure 5 (trimmed data set)
ϕ: H1'–C1'–O1'–C4
ψ: C1'–O1'–C4–H4

$$^{2}J_{C1',C4}\text{ (Hz)} = -2.85 + 0.60\cos\phi - 0.52\sin\phi - 0.07\cos 2\phi - 0.11\sin 2\phi \quad \text{RMSD} = 0.47\text{ Hz} \quad [S9]$$

$$^{2}J_{C2',H1'}\text{ (Hz)} = 2.57 + 0.47\cos\phi - 0.22\sin\phi - 1.48\cos 2\phi - 0.93\sin 2\phi \quad \text{RMSD} = 0.48\text{ Hz} \quad [S10]$$

$^3J_{C2',C4}$ (Hz) = 1.57 + 0.23 cos ϕ + 0.24 sin ϕ – 0.22 cos 2ϕ + 1.86 sin 2ϕ

RMSD = 0.31 Hz [S11]

$^3J_{H1',C4}$ (Hz) = 3.34 – 1.80 cos ϕ + 0.29 sin ϕ + 3.90 cos 2ϕ + 0.68 sin 2ϕ

RMSD = 0.52 Hz [S12]

Structure 5 (constrained data set)
ϕ: H1'–C1'–O1'–C4
ψ: C1'–O1'–C4–H4

$^2J_{C1',C4}$ (Hz) = –2.63 + 0.69 cos ϕ – 0.21 sin ϕ – 0.26 sin 2ϕ

RMSD = 0.31 Hz [S13]

$^2J_{C2',H1'}$ (Hz) = 2.47 + 0.43 cos ϕ + 0.07 sin ϕ – 1.06 cos 2ϕ – 0.85 sin 2ϕ

RMSD = 0.37 Hz [S14]

$^3J_{C2',C4}$ (Hz) = 1.41 + 0.31 cos ϕ + 0.51 sin ϕ + 0.11 cos 2ϕ + 1.80 sin 2ϕ

RMSD = 0.47 Hz [S15]

$^3J_{H1',C4}$ (Hz) = 3.48 – 1.13 cos ϕ + 0.06 sin ϕ + 3.73 cos 2ϕ + 0.77 sin 2ϕ

RMSD = 0.38 Hz [S16]

****

Structure 6 (trimmed data set)
ϕ: H1'–C1'–O1'–C2
ψ: C1'–O1'–C2–H2

$^2J_{C1',C2}$ (Hz) = –2.37 + 0.54 cos ϕ – 0.77 sin ϕ – 0.12 cos 2ϕ – 0.33 sin 2ϕ

RMSD = 0.51 Hz [S17]

$^2J_{C2',H1'}$ (Hz) = 2.63 + 0.43 cos ϕ – 0.51 sin ϕ – 1.48 cos 2ϕ – 1.00 sin 2ϕ

RMSD = 0.45 Hz [S18]

$^3J_{C2',C2}$ (Hz) = 1.57 + 0.29 cos ϕ + 0.29 sin ϕ – 0.13 cos 2ϕ + 1.80 sin 2ϕ

RMSD = 0.32 Hz [S19]

$^3J_{H1',C2}$ (Hz) = 3.58 – 1.41 cos ϕ – 0.16 sin ϕ + 3.71 cos 2ϕ + 0.58 sin 2ϕ

RMSD = 0.56 Hz [S20]

Structure 6 (constrained data set)
ϕ: H1'–C1'–O1'–C2
ψ: C1'–O1'–C2–H2

$^2J_{C1',C2}$ (Hz) = –2.20 + 0.78 cos ϕ – 0.38 sin ϕ + 0.27 cos 2ϕ – 0.26 sin 2ϕ
RMSD = 0.39 Hz [S21]

$^2J_{C2',H1'}$ (Hz) = 2.41 + 0.20 cos ϕ – 1.04 cos 2ϕ – 1.05 sin 2ϕ
RMSD = 0.35 Hz [S22]

$^3J_{C2',C2}$ (Hz) = 1.48 + 0.15 cos ϕ + 0.45 sin ϕ + 0.28 cos 2ϕ + 1.63 sin 2ϕ
RMSD = 0.31 Hz [S23]

$^3J_{H1',C2}$ (Hz) = 3.64– 1.01 cos ϕ – 0.51 sin ϕ + 3.84 cos 2ϕ + 0.82 sin 2ϕ
RMSD = 0.33 Hz [S24]

****

Structure 7 (trimmed data set)
ϕ: H1'–C1'–O1'–C4
ψ: C1'–O1'–C4–H4

$^2J_{C1',C4}$ (Hz) = –2.50 + 0.73 cos ϕ – 0.83 sin ϕ – 0.08 cos 2ϕ – 0.33 sin 2ϕ
RMSD = 0.39 Hz [S25]

$^2J_{C2',H1'}$ (Hz) = 4.43 + 0.53 cos ϕ – 0.14 sin ϕ – 1.33 cos 2ϕ – 1.33 sin 2ϕ
RMSD = 0.47 Hz [S26]

$^3J_{C2',C4}$ (Hz) = 1.76 + 0.29 cos ϕ – 0.10 sin ϕ – 0.51 cos 2ϕ + 1.89 sin 2ϕ
RMSD = 0.29 Hz [S27]

$^3J_{H1',C4}$ (Hz) = 3.56 – 1.57 cos ϕ + 3.79 cos 2ϕ + 0.57 sin 2ϕ
RMSD = 0.49 Hz [S28]

Structure 7 (constrained data set)
ϕ: H1'–C1'–O1'–C4
ψ: C1'–O1'–C4–H4

$^2J_{C1',C4}$ (Hz) = –2.23 + 0.90 cos ϕ – 0.75 sin ϕ – 0.12 cos 2ϕ – 0.42 sin 2ϕ
RMSD = 0.22 Hz [S29]

$^2J_{C2',H1'}$ (Hz) = 4.52 + 0.50 cos ϕ – 1.17 cos 2ϕ – 1.31 sin 2ϕ
RMSD = 0.32 Hz [S30]

$^3J_{C2',C4}$ (Hz) = 1.79 + 0.32 cos ϕ – 0.14 sin ϕ – 0.40 cos 2ϕ + 1.91 sin 2ϕ
RMSD = 0.46 Hz [S31]

$^3J_{H1',C4}$ (Hz) = 3.72 – 0.88 cos ϕ – 0.06 sin ϕ + 3.85 cos 2ϕ + 0.69 sin 2ϕ
RMSD = 0.26 Hz [S32]

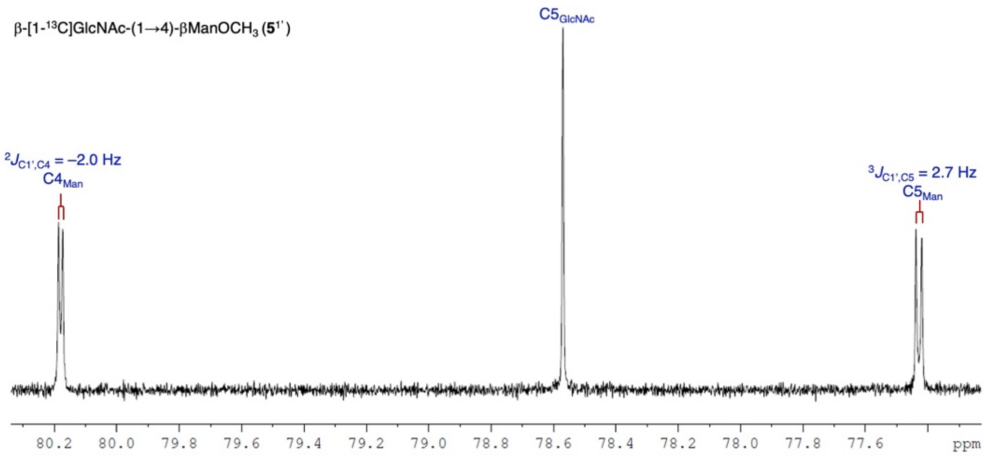

Figure S8. Partial 1D $^{13}C\{^{1}H\}$ NMR spectrum (200 MHz) of **5**$^{1'}$ in $^{2}H_2O$ at ~25 °C showing signals arising from carbons in the βMan and βGlcNAc residues as shown. Signal splittings were used to determine the J_{CC} values shown.

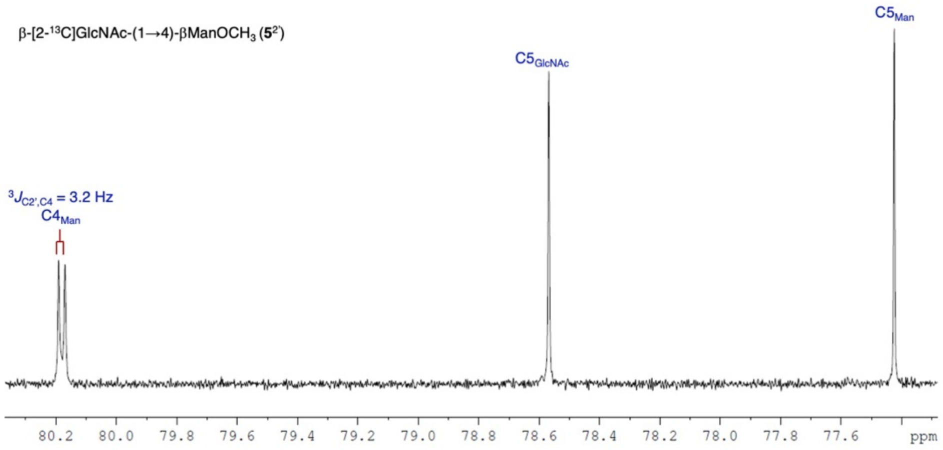

Figure S9. Partial 1D $^{13}C\{^{1}H\}$ NMR spectrum (200 MHz) of $\mathbf{5}^{2'}$ in $^{2}H_2O$ at ~25 °C showing signals arising from carbons in the βMan and βGlcNAc residues as shown. Signal splittings were used to determine the J_{CC} values shown.

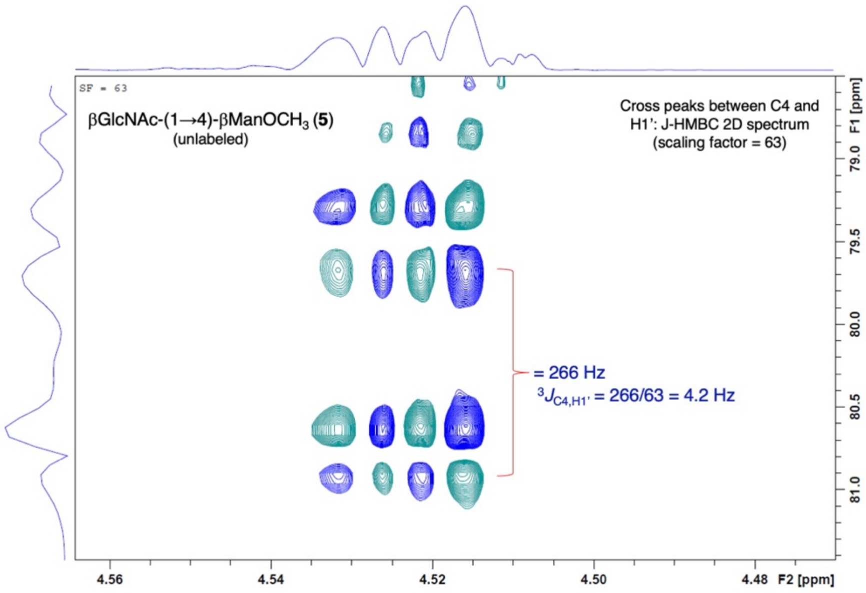

Figure S10. Partial 2D J-HMBC spectrum (800 MHz) of **5** in $^{2}H_2O$ at ~25 ^{o}C showing cross peaks between C4 and H1'. Cross peak splitting in the ^{13}C dimension (F1) was used to determine the trans-*O*-glycosidic $^{3}J_{C4,H1'}$ value as shown.

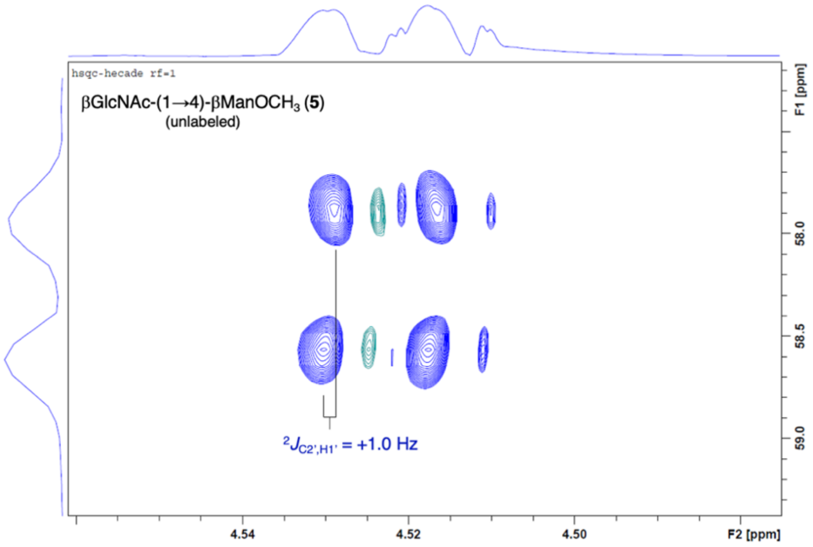

Figure S11. Partial 2D HSQC-HECADE spectrum (800 MHz) of **5** in ^2H_2O at ~25 ºC showing cross peaks between C2’ and H1’. Cross peak offset in the ^1H dimension (F2) was used to determine the intra-residue $^3J_{C2',H1'}$ value as shown.

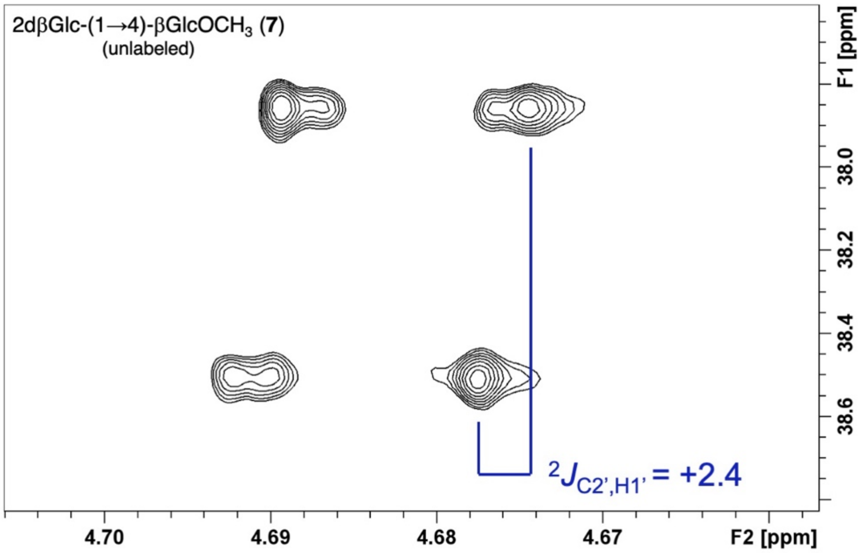

Figure S12. Partial 2D HSQC-HECADE spectrum (800 MHz) of **7** in $^{2}H_2O$ at ~25 ^{o}C showing cross peaks between C2' and H1'. Cross peak offset in the ^{1}H dimension (F2) was used to determine the intra-residue $^{3}J_{C2',H1'}$ value as shown.

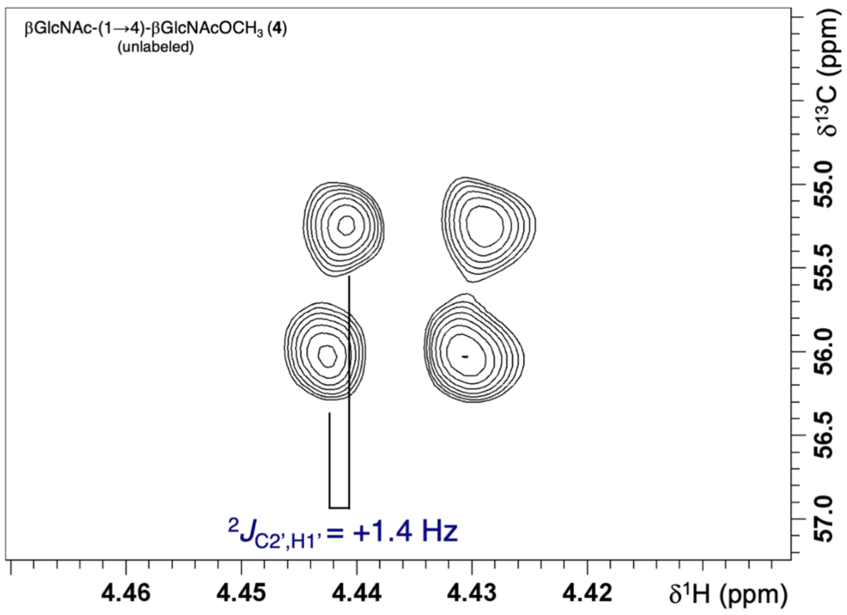

Figure S13. Partial 2D HSQC-HECADE spectrum (800 MHz) of **4** in $^{2}H_2O$ at ~25 ^{o}C showing cross peaks between C2' and H1'. Cross peak offset in the ^{1}H dimension (F2) was used to determine the intra-residue $^{3}J_{C2',H1'}$ value as shown.

Preparation of Disaccharide $4^{1',4}$

(Methyl 2-Acetamido-2-deoxy-β-D-[1-^{13}C]glucopyranosyl-(1→4)-2-acetamido-2-deoxy-β-D-[4-^{13}C]glucopyranoside)

A. Preparation of Donor F (Scheme S1)

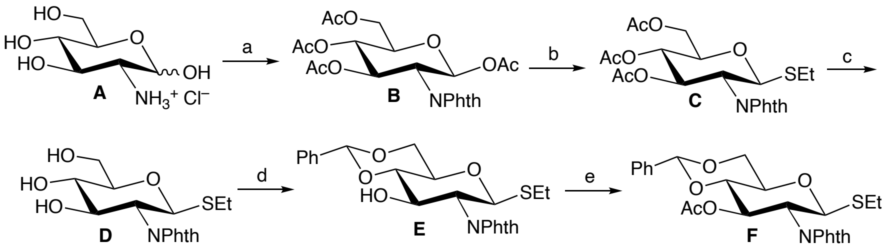

Scheme S1. Reagents and conditions: (a) (i) NaOH, MeOH:H_2O, rt, 1 h; (ii) phthalic anhydride, acetone, 15 °C, 2 h; (iii) $NaHCO_3$, 50 °C, 30 min; (iv) HCl, 20 °C, 1 h, filtered; (v) NaOAc, Ac_2O, refluxed, 30 min, 83%. (b) EtSH, DCM, $BF_3{\cdot}Et_2O$, 5 °C, 5 h, 85%. (c) MeOH, NaOMe, rt, 20 min. (d) $PhCH(OMe)_2$, *p*-TsOH, CH_3CN, rt, 6 h, 88%. (e) Ac_2O, py, rt, 6 h, 85%.

A1. *1,3,4,6-Tetra-O-acetyl-2-deoxy-2-N-phthalimido-β-D-glucopyranose* (***B***)[1]. To a solution of D-glucosamine hydrochloride (**A**) (10 g, 46.5 mmol) in methanol-distilled water (60 mL, 1:2 *v*/*v*) was added sodium hydroxide (1.9 g, 46.5 mmol) and the reaction mixture was allowed to stir at rt for 1 h. The reaction mixture was then cooled to 15 °C and a solution of phthalic anhydride (8 g, 54.0 mmol) in acetone (40 mL) was added to it slowly maintaining the temperature below 15 °C. After stirring at rt for 2 h, solid $NaHCO_3$ (8 g, 95.2 mmol) was added in portions and the reaction mixture was allowed to stir at 50 °C for 30 min. The reaction mixture was then stirred at rt for 12 h. The reaction mixture was neutralized with cold HCl maintaining the temperature below 20 °C. On cooling the resulting reaction mixture, 2-deoxy-2-*N*-phthalimido-α/β-D-glucopyranose precipitated as a white solid. The solid product was collected by filtration, washed with cold distilled water, and dried. To a suspension of crude product in acetic anhydride (100 mL, 1.06 mol) was added anhydrous sodium acetate (24 g, 291.1 mmol) and the reaction mixture was refluxed for 30 min. After cooling, the reaction mixture was diluted with CH_2Cl_2 (200 mL) and washed successively with distilled water and satd. aqueous $NaHCO_3$. The organic layer was dried over anhydrous Na_2SO_4 and concentrated to a yellow syrup. Column chromatography of the crude product on silica gel, using hexane-EtOAc (8:1 *v*/*v*) as the eluant, gave pure compound **B**[1] (18.4 g, 83%) as a white solid.

A2. *Ethyl 3,4,6-Tri-O-acetyl-2-deoxy-2-N-phthalimido-1-thio-β-D-glucopyranoside* (***C***)[2]. To a stirred solution of **B** (12 g, 25.9 mmol) in anhydrous CH_2Cl_2 (40 mL) were added 4Å molecular sieves (4 g), EtSH (7.6 mL, 103.7 mmol) and $BF_3{\cdot}Et_2O$ (9.8 mL, 77.7 mmol), and the resulting reaction mixture was stirred at 5 °C for 5 h. The reaction mixture was filtered and the washed with CH_2Cl_2 (150 mL). The organic layer was washed with satd. aqueous $NaHCO_3$ and distilled water, dried over anhydrous Na_2SO_4, and concentrated. The crude product was purified on silica gel using hexane-EtOAc (5:1 *v*/*v*) as the eluant to afford pure compound (**C**)[2] (10.2 g, 85%) as a yellow oil.

A3. *Ethyl 4,6-O-Benzylidene-2-deoxy-2-N-phthalimido-1-thio-β-D-glucopyranoside* (***E***)[3]. A solution of **C** (3 g, 6.3 mmol) in 0.05 *M* CH_3ONa in CH_3OH (25 mL) was stirred at rt for 20 min.

The reaction mixture was neutralized with batchwise addition of Dowex HCR (H^+) ion-exchange resin, vacuum-filtered, and the filtrate was concentrated to dryness to give an amorphous solid (**D**) in quantitative yield. To a solution of the crude mass in anhydrous CH_3CN (15 mL) were added benzaldehyde dimethylacetal (2.3 mL, 12 mmol) followed by *p*-TsOH (300 mg, 1.78 mmol), and the reaction mixture was stirred at rt for 10 h. The reaction was quenched with Et_3N (1 mL) and the reaction mixture was evaporated to dryness. The crude mass was purified on silica gel using hexane-EtOAc (3:1 *v*/*v*) as the eluant to give pure compound (**E**)[3] (2.43 g, 88%) as a white solid. <u>^{1}H NMR</u> (400 MHz, $CDCl_3$): δ 7.80–7.26 (m, 9 H, Ar-H), 5.57 (s, 1 H, PhC*H*), 5.42–5.39 (d, *J* = 10.6 Hz, 1 H, H-1), 4.66–4.40 (m, 1 H, H-4), 4.39–4.29 (m, 2 H, H-6_a, H-6_b), 3.83 (t, *J* = 10.1 Hz each, 1 H, H-3), 3.69–3.60 (m, 1 H, H-5), 3.58 (t, *J* = 9.2 Hz each, 1 H, H-2), 2.71–2.63 (m, 2 H, SCH_2CH_3), 1.21 (t, *J* = 7.4 Hz each, 3 H, SCH_2CH_3). <u>^{13}C NMR</u> (100 MHz, $CDCl_3$): δ 168.5, 167.9 (2 Phth), 134.4–123.5 (Ar-C), 102.2 (Ph*C*H), 82.3 (C-1), 82.1 (C-4), 70.5 (C-3), 69.7 (C-6), 68.8 (C-5), 55.6 (C-2), 24.4 (S*C*H_2CH_3), 15.1 (SCH_2*C*H_3).

A4. *Ethyl 3-O-Acetyl-4,6-O-benzylidene-2-deoxy-2-N-phthalimido-1-thio-β-D-glucopyranoside (**F**)*[4]. To a solution of **E** (2 g,4.5 mmol) in pyridine (15 mL) was added acetic anhydride (10 mL, 108.1 mmol), and and the reaction mixture was stirred at rt for 6 h. The solvents were removed under reduced pressure to give the crude product, which was purified on silica gel using hexane-EtOAc (3:1 *v*/*v*) as the eluant to give pure compound **F**[4] (1.9 g, 85%) as a white solid. <u>^{1}H NMR</u> (400 MHz, $CDCl_3$): δ 7.87–7.26 (m, 9 H, Ar-H), 5.92 (t, *J* = 9.3 Hz each, 1 H, H-3), 5.59 (d, *J* = 8.0 Hz, 1 H, H-1), 5.55 (s, 1 H, PhC*H*), 4.43–4.34 (m, 2 H, H-2, H-5), 3.81–3.75 (m, 3 H, H-4, H-6_{ab}), 2.71–2.66 (m, 2 H, SCH_2CH_3), 1.89 (s, 3 H, $COCH_3$), 1.20 (t, *J* = 7.4 Hz each, 3 H, SCH_2CH_3). <u>^{13}C NMR</u> (100 MHz, $CDCl_3$): δ 170.3 (*C*OCH_3), 167.5, 167.6 (2 *C*O, Phth), 134.3–123.6 (Ar-C), 102.0 (Ph*C*H), 82.0 (C-1), 79.6 (C-4), 70.9 (2 C, C-3, C-5), 69.0 (C-6), 54.6 (C-2), 24.7 (S*C*H_2CH_3), 20.9 (CO*C*H_3), 15.3 (SCH_2*C*H_3).

<u>B. Preparation of Acceptor L (Scheme S2)</u>

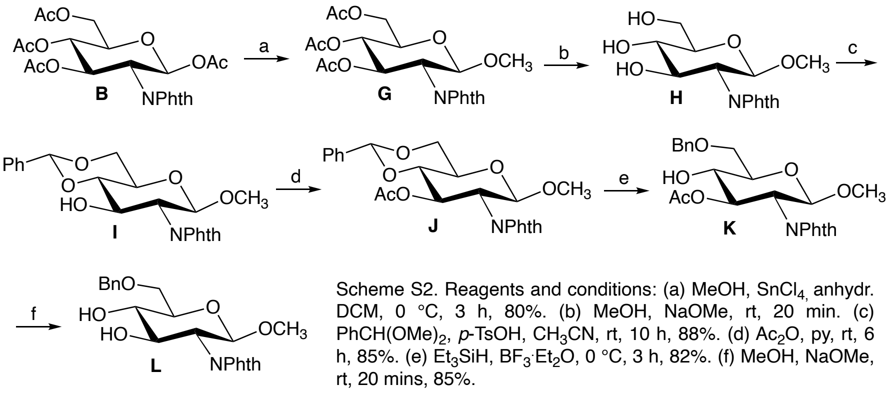

Scheme S2. Reagents and conditions: (a) MeOH, $SnCl_4$, anhydr. DCM, 0 °C, 3 h, 80%. (b) MeOH, NaOMe, rt, 20 min. (c) $PhCH(OMe)_2$, *p*-TsOH, CH_3CN, rt, 10 h, 88%. (d) Ac_2O, py, rt, 6 h, 85%. (e) Et_3SiH, $BF_3{\cdot}Et_2O$, 0 °C, 3 h, 82%. (f) MeOH, NaOMe, rt, 20 mins, 85%.

B1. *Methyl 3,4,6-Tri-O-acetyl-2-deoxy-2-N-phthalimido-β-D-glucopyranoside (**G**)*[5]. To a stirred solution of **B** (5 g, 10.47 mmol) and methanol (0.636 mL, 15.70 mmol) in anhydrous CH_2Cl_2 (30 mL) was stirred under nitrogen for 30 min. To the reaction mixture was added stannic chloride (4.80 mL, 10.91 g, 41.89 mmol) dropwise at 0 °C, and the reaction was continued at rt. After 3 h, TLC (10:1 chloroform-acetone) showed the formation of a single compound. The reaction mixture was added to a satd. aqueous solution of $NaHCO_3$ and the mixture was extracted with $CHCl_3$.

The organic extract was washed with distilled water, dried over anhydrous Na_2SO_4, and concentrated. Crystallization from methanol gave **G**[5] (4.2 g, 80%) as a white solid. <u>^{1}H NMR</u> (400 MHz, $CDCl_3$): δ 7.85–7.74 (m, 4 H, Ar-H), 5.81–5.76 (t, *J* = 9.3 Hz each, 1 H, H-3), 5.31 (d, *J* = 8.0 Hz, 1 H, H-1), 5.21–5.16 (t, 1 H, H-2), 4.36–4.28 (m, 2 H, H-4, H-6_b), 4.21–4.18 (dd, 1 H, H-6_a), 3.89–3.87 (m, 1 H, H-5), 3.45 (s, 3 H, OCH_3), 2.12, 2.03, 1.86 (3 s, 9 H, 3 $COCH_3$). <u>^{13}C NMR</u> (100 MHz, $CDCl_3$): δ 171.0, 170.4, 169.7, (3 $COCH_3$), 167.5, 167.6 (2 *C*O, Phth), 134.5–123.8 (Ar-C), 99.2 (C-1), 72.0 (C-4), 71.0 (C-3), 69.2 (C-6), 62.5 (C-5), 57.3 (OCH_3) 54.7 (C-2), 21.0, 20.8, 20.6 (3 $COCH_3$).

B2. *Methyl 4,6-O-Benzylidene-2-deoxy-2-N-phthalimido-β-D-glucopyranoside (**I**)*[3]. A solution of **G** (3 g, 6.67 mmol) in 0.05 *M* CH_3ONa in CH_3OH (25 mL) was stirred at rt for 20 min. The reaction mixture was neutralized with batchwise addition of Dowex HCR (H^+) ion-exchange resin, vacuum-filtered, and the filtrate was evaporated to dryness to give an amorphous solid (**H**) in quantitative yield. To a solution of the crude mass in anhydrous CH_3CN (15 mL) were added benzaldehyde dimethylacetal (2.3 mL, 12 mmol) followed by *p*-TsOH (300 mg, 1.78 mmol), and the resulting reaction mixture was stirred at rt for 10 h. The reaction was quenched with the addition of Et_3N (1 mL) and the reaction mixture was evaporated to dryness. The crude mass was purified on silica gel using hexane-EtOAc (3:1 *v*/*v*) as the eluant to give pure compound **I**[3] (2.43 g, 88%) as a white solid. <u>^{1}H NMR</u> (400 MHz, $CDCl_3$): δ 7.83–7.36 (m, 9 H, Ar-H), 5.56 (s, 1 H, PhC*H*), 5.18 (d, *J* = 8.0 Hz, 1 H, H-1), 4.63–4.58 (t, 1 H, H-3), 4.40–4.38 (dd, 1 H, H-6_a), 4.23–4.19 (t, 1 H, H-2), 3.85–3.80 (m, 1 H, H-5), 3.61–3.57 (m, 2 H, H-4, H-6_b), 3.43 (s, 3 H, OCH_3). <u>^{13}C NMR</u> (100 MHz, $CDCl_3$): δ 168.4 (2 *C*O, Phth), 134.3–126.5 (Ar-C), 102.1 (C-1), 100.0 (Ph*C*H), 82.4 (C-4), 68.9 (C-3), 68.7 (C-6), 66.4 (C-5), 57.3 (OCH_3) 56.8 (C-2).

B3. *Methyl 3-O-Acetyl-4,6-O-benzylidene-2-deoxy-2-N-phthalimido-β-D-glucopyranoside (**J**)*[3]. To a solution of **I** (2 g,4.86 mmol) in pyridine (15 mL) was added acetic anhydride (10 mL, 97.2 mmol),and the reaction mixture was stirred at rt for 6 h. The solvents were evaporated under reduced pressure to give a crude product, which was purified on silica gel using hexane-EtOAc (3:1 *v*/*v*) as the eluant to furnish pure compound **J**[3] (1.9 g, 85%) as a white solid.

B4. *Methyl 3-O-Acetyl-6-O-benzyl-2-deoxy-2-N-phthalimido-β-D-glucopyranoside (**K**)*[3]. To a solution of **J** (2.0 g, 4.41 mmol) in CH_2Cl_2 (15 mL) were added triethylsilane (4.22 mL, 26.46 mmol) and $BF_3{\cdot}Et_2O$ (0.544 mL, 4.41 mmol), and the reaction mixture was stirred at 0 °C for 3 h. The reaction mixture was poured into distilled water (200 mL) and the mixture was extracted with CH_2Cl_2 (100 mL). The organic layer was washed successively with satd. aqueous $NaHCO_3$ and distilled water, dried over anhydrous Na_2SO_4, and concentrated. The solvents were removed under reduced pressure and the crude product was purified on silica gel using hexane-EtOAc (1:1 *v*/*v*) as the eluant to give pure compound **K**[3] (1.2 g, 82%) as a white solid. <u>^{1}H NMR</u> (400 MHz, $CDCl_3$): δ 7.87–7.36 (m, 9 H, Ar-H), 5.70–5.65 (t, *J* = 9.3 Hz each, 1 H, H-3), 5.46 (d, *J* = 8.0 Hz, 1 H, H-1), 4.70 (d, *J* = 12.0 Hz, 1 H, $PhCH_2$), 4.65 (d, *J* = 12.0 Hz, 1 H, $PhCH_2$), 4.60 (t, 1 H, H-2), 4.34 (m, 1 H, H-5), 3.90–3.70 (m, 3 H, H-4, H-6_{ab}), 3.44 (s, 3 H, OCH_3), 1.93 (s, 3 H, $COCH_3$). <u>^{13}C NMR</u> (100 MHz, $CDCl_3$): δ 170.3 ($COCH_3$), 168.4 (2 *C*O, Phth), 134.4–123.7 (Ar-C), 99.2 (C-1), 74.3 (C-4), 74.0 (C-3), 73.8 (C-6), 71.8 (C-5), 57.1 (OCH_3), 54.7 (C-2), 20.9 ($COCH_3$).

B5. *Methyl 6-O-Benzyl-2-deoxy-2-N-phthalimido-β-D-glucopyranoside (**L**)*[6]. A solution of **K** (3 g, 6.3 mmol) in 0.05 *M* CH_3ONa in CH_3OH (25 mL) was stirred at rt for 20 min. The reaction

mixture was neutralized with batchwise addition of Dowex HCR (H^+) ion-exchange resin, vacuum-filtered, and the filtrate was evaporated to dryness to give an amorphous solid in quantitative yield. The crude product was purified on silica gel using hexane-EtOAc (1:1 *v*/*v*) as the eluant to give pure compound **L**[6] (1.6 g, 85%) as a white solid. ^{1}H NMR (400 MHz, $CDCl_3$): δ 7.83–7.35 (m, 9 H, Ar-H), 5.14 (d, *J* = 8.0 Hz, 1 H, H-1), 4.64 (d, *J* = 12.0 Hz, 1 H, PhC*H*$_2$), 4.61 (d, *J* = 12.0 Hz, 1 H, PhC*H*$_2$), 4.31 (t, 1 H, H-2), 4.15–4.10 (m, 1 H, H-5), 3.83–3.78 (m, 2 H, H-6_{ab}), 3.64–3.60 (m, 2 H, H-3, H-4), 3.41 (s, 3 H, OC*H*$_3$). ^{13}C NMR (100 MHz, $CDCl_3$): δ 167.5, 167.6 (2 *C*O, Phth), 134.3–123.6 (Ar-C), 99.4 (C-1), 74.3 (C-4), 74.0 (C-3), 73.8 (C-6), 71.9 (C-5), 57.0 (O*C*H$_3$), 56.3 (C-2).

C. Condensation of Donor **F** and Acceptor **L** To Give Disaccharide **4** (Scheme S3)

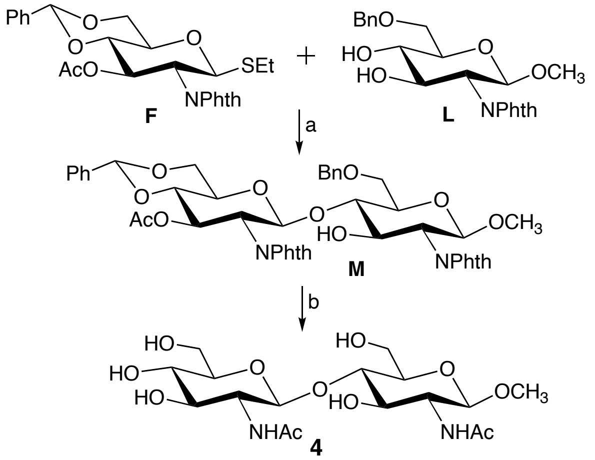

Scheme S3. Reagents and conditions: (a) NIS, TMSOTf, anhydr. DCM, –40 °C, 1 h, 82%. (b) (i) NH_2NH_2, EtOH, 70 °C, 24 h; (ii) Ac_2O, py, rt, 3 h; (iii) CH_3OH, NaOMe, rt, 3 h; (iv) H_2, Pd/C, MeOH, rt, 24 h, 60%.

C1. *Methyl 3-O-Acetyl-4,6-benzylidene-2-deoxy-2-phthalimido-β-D-glucopyranosyl]-(1→4)-6-O-benzyl-2-deoxy-2-phthalimido-β-D-glucopyranoside (**M**)*[7]. To a solution of **L** (200 mg, 0.48 mmol) and **F** (351 mg, 0.72 mmol) in anhydrous CH_2Cl_2 (5 mL) was added 4Å molecular sieves (2.0 g), and the reaction mixture was cooled to –40 °C. To the cooled reaction mixture were added *N*-iodosuccinimide (180 mg, 0.79 mmol) and TMSOTf (13μL, 0.07 mmol), and the reaction mixture was stirred at –40 °C for 1 h. The reaction mixture was filtered through a Celite® pad and the pad was washed with CH_2Cl_2 (100 mL). The organic layer was washed successively with 5% aqueous $Na_2S_2O_3$, satd. aqueous $NaHCO_3$ and distilled water, dried over anhydrous Na_2SO_4, and concentrated under reduced pressure. The crude product was purified on silica gel using hexane-EtOAc (1:1 *v*/*v*) as the eluant to give pure **M**[7] (200 mg, 82%) as a white solid. ^{1}H NMR (400 MHz, $CDCl_3$): δ 7.84–7.10 (m, 18 H, Ar-H), 5.91–5.85 (t, 1 H, H-3_B), 5.58–5.56 (d, *J* = 8.0 Hz, 1 H, H-1_B), 5.49 (s, 1 H, PhC*H*), 5.03–5.01 (d, *J* = 8.0 Hz, H-1_A), 4.38–4.36 (m, 3 H, -C*H*$_2$-, 2 PhC*H*$_2$, H-5_B), 4.14–4.09 (m, 4 H, H-3_A, H-6_{abA}, H-2_A), 3.91 (m, 1 H, H-4_B), 3.76–3.65 (m, 4 H, H-6_{abB}, H-4_A, H-2_B), 3.34 (s, 3 H, OC*H*$_3$), 3.29 (m, 1 H, H-5_A), 1.88 (COC*H*$_3$). ^{13}C NMR (100 MHz, $CDCl_3$): δ 170.1 (CO*C*H$_3$), 167.5, 167.6 (2 *C*O, Phth), 134.3–123.9 (Ar-C), 101.9 (C-1_A), 99.6 (Ph*C*H), 99.1 (C-1_B), 81.9 (C-4_A), 78.8 (C-3_B), 74.2 (C-3_A), 73.1 (Ph*C*H$_2$), 70.0 (C-4_B),

69.6 (C-6_A), 68.4 (C-5_A), 68.0 (C-6_B), 66.3 (C-5_B), 56.7 (OCH_3), 55.9 (C-2_B), 55.5 (C-2_A), 20.7 (CO*C*H_3).

C2. *Methyl 2-Acetamido-2-deoxy-β-D-glucopyranosyl-(1→4)-2-acetamido-2-deoxy-β-D-glucopyranoside (4).* To a solution of **M** (200 mg, 0.23 mmol) in EtOH (2 mL) was added NH_2NH_2 (0.5 mL, 15.64 mmol), and the reaction mixture was stirred at 70 °C for 24 h. The solvents were removed under reduced pressure, and the crude product was dissolved in pyridine (3 mL) and acetic anhydride (1 mL, 10.58 mmol) and the solution kept at rt for 3 h. The solvents were removed under reduced pressure to give the crude acetylated product in quantitative yield after purification on a silica gel column using ethyl acetate-hexane (1:1 *v*/*v*) as the solvent. To a solution of acetylated product was added CH_3OH and CH_3ONa, and the reaction mixture was stirred at rt for 3 h. The reaction mixture was neutralized with Dowex HCR (H^+) ion-exchange resin, filtered, and concentrated *in vacuo* to dryness to afford a crude product. To a solution of the *N*-acetylated product in CH_3OH (5 mL) was added Pd-C (50 mg), and the reaction mixture was stirred at rt under a positive pressure of H_2 for 24 h. The reaction mixture was then filtered through a Celite® pad, the pad was washed with CH_3OH/H_2O (20 mL, 2:1 *v*/*v*), and the filtrates were collected and concentrated under reduced pressure. The deprotected product was purified on a column (2.5 cm x 100 cm) containing Dowex 50 x 8 (200-400 mesh) ion-exchange resin in the Ca^{2+} form[8] using distilled water as the eluant to give pure disaccharide (**4**) (80 mg, 60%). See Tables S1–S3 for ^1H and ^{13}C chemical shifts, and ^1H-^1H NMR spin-couplings, in **4**. Representative ^1H and $^{13}C\{^1H\}$ NMR spectra of **4** are shown in Figures S1–S3. HRMS (ESI-TOF) *m/z* $[M+Na]^+$: calcd. for $C_{17}H_{30}N_2O_{11}Na$, 461.1742; found, 461.1744.

D. *Preparation of Disaccharide $4^{1',4}$*. ^{13}C-Labeled disaccharide $\mathbf{4}^{1',4}$ was prepared by the route described above by substituting D-[1-^{13}C]glucosamine hydrochloride for **A** in Scheme S1 to give ethyl 3-*O*-acetyl-4,6-*O*-benzylidene-2-deoxy-2-*N*-phthalimido-1-thio-β-D-[1-^{13}C]glucopyranoside ($\mathbf{F}^1$), and D-[4-^{13}C]glucosamine hydrochloride for **A** in Scheme S1 to prepare 1,3,4,6-tetra-O-acetyl-2-deoxy-2-*N*-phthalimido-β-D-[4-^{13}C]glucopyranose ($\mathbf{B}^4$) for use in Scheme S2. The singly ^{13}C-labeled D-glucosamines were obtained from Omicron Biochemicals, Inc. (South Bend, IN).

References

1. R. U. Lemieux, T. Takeda and B. Y. Chung, *Synthetic Methods for Carbohydrates,* 1976, **39**, 90–115.
2. J. Xia, C. F. Piskorz, J. L. Alderfer, R. D. Locke and K. L. Matta, *Tetrahedron Lett.,* 2000, **41**, 2773–2776.
3. M. Jana, A. Ghosh, A. Santra, R. K. Kar, A. K. Misra and A. Bhunia, A., *J. Colloid Interface Sci.,* 2017, **498**, 395–404.
4. I. Bhaumik, T. Ghosh and A. K. Misra, *Carbohydr. Res.,* 2014, **399**, 21–25.
5. M. T. Campos-Val Des, J. R. Marino-Albernas and V. Verez-Bencomo, *J. Carbohydr. Chem.,* 1987, **6**, 509–513.
6. R. K. Jain, R. Vig, R. Rampal, E. V. Chandrasekaran and K. L. Matta, *J. Am. Chem. Soc.*, 1994, **116**, 12123–12124.
7. J. Zhang, A. Otter and D. R. Bundle, *Bioorg. Med. Chem.,* 1996, **11**, 1989–2001.
8. S. J. Angyal, G. S. Bethell and R. J. Beveridge, R. J., *Carbohydr. Res.*, 1979, **73**, 9–18.

Table S1. ^{1}H Chemical Shifts[a] of βGlcNAc-(1→4)-βGlcNAcOCH$_3$ (**4**).

nucleus	GlcNAc residue	
	a	b
H1	4.459	4.609
H2	3.746	3.772[b]
H3	3.709	3.592
H4	3.632	*nm*
H5	*nm*	*nm*
H6*R*[c]	3.693	3.773
H6*S*[c]	3.889	3.941
OCH$_3$	3.522	
CH$_3$ (acetyl)	2.053	2.097

[a]In ppm, referenced to the internal residual HO^{2}H signal at 4.810 ppm; measured at 25 °C in ^2H_2O solvent; ± 0.001 ppm. An "*nm*" entry denotes values that were *n*ot *m*easured. [b]H2 (residue b) chemical shift, ± 0.005 ppm. [c]Stereochemical assignments of the H6*R* and H6*S* signals were made by analogy to those for methyl β-D-glucopyranoside in which the H6*S* signal is downfield of the H6*R* signal (see: Thibaudeau *et al.*, *J. Am. Chem. Soc.* **2004**, *126*, 15668–15685).

Table S2. ^{13}C Chemical Shifts[a] of βGlcNAc-(1→4)-βGlcNAcOCH$_3$ (**4**).

nucleus	GlcNAc residue	
	a	b
C1	101.78	101.41
C2	54.78	55.51
C3	72.53	73.37
C4	79.40	69.62
C5	74.43	75.83
C6	60.06	60.45
OCH$_3$	57.05	
CO (acetyl)	174.62	174.55
CH$_3$ (acetyl)	22.08	22.04

[a]In ppm relative to external DSS; measured at 25 °C in ^2H_2O solvent; ± 0.01 ppm.

Table S3. ^{1}H-^{1}H Spin-coupling Constants[a] in βGlcNAc-(1→4)-βGlcNAcOCH$_3$ (**4**).

J-coupling	GlcNAc residue	
	a	b
$^3J_{H1,H2}$	8.3	8.5
$^3J_{H2,H3}$	10.4	10.5
$^3J_{H3,H4}$	8.3	8.6
$^3J_{H4,H5}$	9.8	*nm*
$^3J_{H5,H6R}$	5.6	5.7
$^3J_{H5,H6S}$	2.2	2.2
$^2J_{H6R,H6S}$	–12.2	–12.4

[a]In Hz at 25 °C in ^2H_2O solvent; ± 0.1 Hz. All values have positive signs except those shown with a (–) prefix. The entry "*nm*" denotes values that were *n*ot *m*easured.

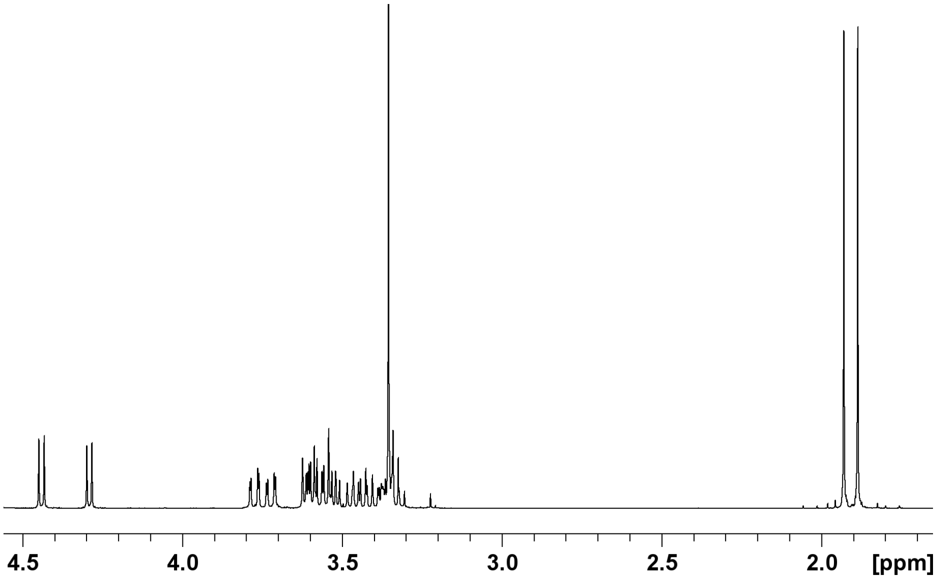

Figure S1. Partial ^{1}H NMR spectrum (800 MHz) of disaccharide **4** in ^2H_2O at ~25 ^oC. The signals from the anomeric hydrogens appear between 4.2–4.5 ppm (two doublets), and those from the two methyl group hydrogens of the *N*-acetyl side-chains appear most upfield at ~1.9 ppm (two singlets).

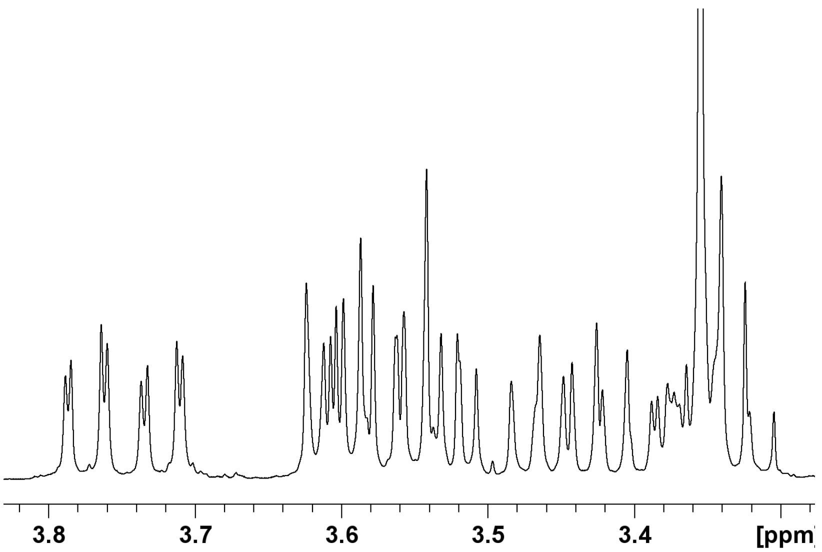

Figure S2. Expansion of the ^1H NMR spectrum in Figure S1, showing signals from the non-anomeric hydrogens of both GlcNAc residues, and the intense singlet at ~ 3.35 ppm from the hydrogens of the aglycone methyl group.

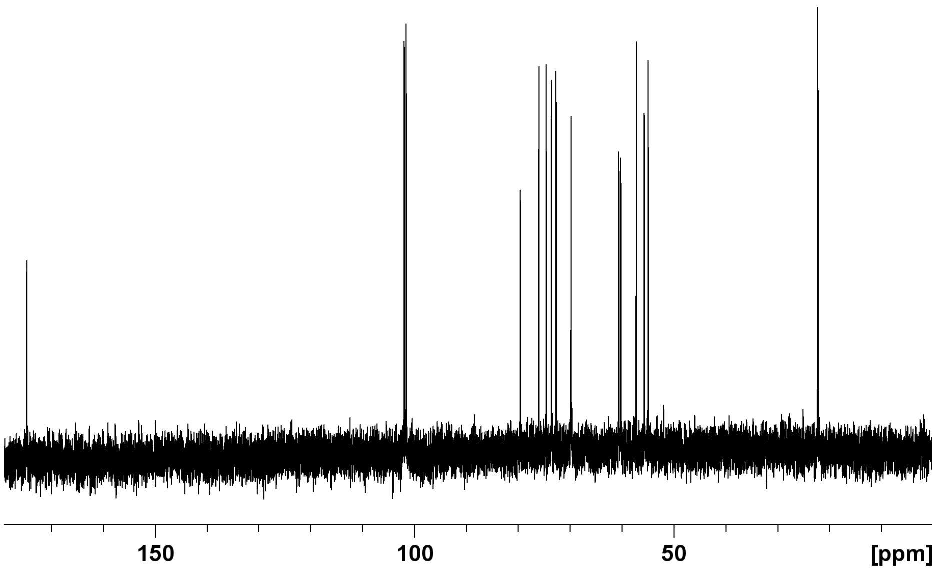

Figure S3. The $^{13}C\{^1H\}$ NMR spectrum (200 MHz) of **4** in ^2H_2O at ~ 25 °C. The most downfield signals arise from the carbonyl carbons of the *N*-acetyl side-chains, and the most upfield signals arise from the methyl carbons of the *N*-acetyl side-chains. The two anomeric carbon signals appear at ~100 ppm.

Preparation of Disaccharides 5[1'] and 5[2']

(Methyl 2-Acetamido-2-deoxy-β-D-[1-^{13}C]glucopyranosyl-(1→4)-β-D-mannopyranoside and Methyl 2-Acetamido-2-deoxy-β-D-[2-^{13}C]glucopyranosyl-(1→4)-β-D-mannopyranoside)

A. Preparation of Glycosyl Acceptor **C** and Glycosyl Donor **G** (Scheme S1)

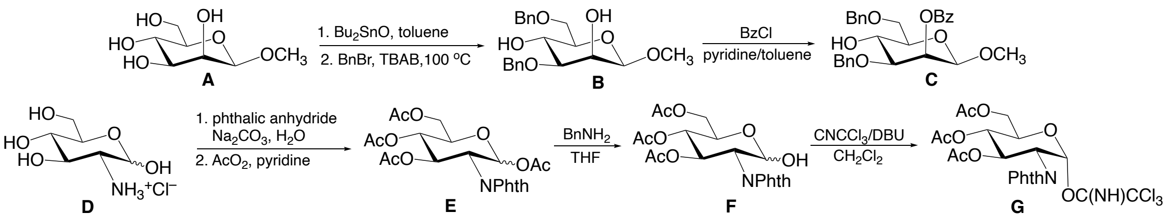

Scheme S1. Preparation of glycosyl acceptor **C** and glycosyl donor **G**.

A1. *Methyl 2-O-Benzoyl-3,6-di-O-benzyl-β-D-mannopyranoside* (**C**). Methyl β-D-mannopyranoside (**A**) (5.90 g, 30.4 mmol) and dibutyltin oxide (17.0 g, 68.3 mmol) were added to anhydrous toluene (60 mL). After stirring at 100 °C for 3 h, the reaction mixture was concentrated to 30 mL, and benzyl bromide (20 mL, 168 mmol) and tetrabutylammonium bromide (5.00 g, 15.5 mmol) were added. The resulting mixture was stirred at 100 °C for an additional 20 h, and then concentrated *in vacuo*. The residue was dissolved in ethyl acetate, washed with distilled water, dried over anhydrous Na_2SO_4, and concentrated to a syrup, which was purified by flash chromatography on silica gel to afford glycoside **B** (7.50 g, 20.1 mmol, 66%).[1] In this and the following steps, flash column chromatography on silica gel (preparative scale) was performed on a Reveleris® X2 flash chromatography system using a mixture of hexanes and ethyl acetate as the eluent. Compound **B** (7.50 g, 20.1 mmol) was dissolved in anhydrous toluene (60 mL) and anhydrous pyridine (8 mL) was added. Benzoyl chloride (2.50 mL, 21.5 mmol) was then added dropwise at 0 °C and the reaction mixture was stirred at 0 °C for 2 h. The mixture was evaporated to dryness and purified by flash chromatography on silica gel, affording product **C** (8.40 g, 17.6 mmol, 87%). **C**: ^{1}H NMR (600 MHz, $CDCl_3$): δ 8.12 (m, 2H), 7.57–7.26 (m, 13H), 5.85 (dd, *J* = 3.1, 1.0 Hz, H-2, 1H), 4.86 (d, *J* = 11.3 Hz, PhC*H*$_2$, 1H), 4.74 (d, *J* = 12.0 Hz, PhC*H*$_2$, 1H), 4.66 (d, *J* = 12.0 Hz, PhC*H*$_2$, 1H), 4.52 (d, *J* = 1.0 Hz, H-1, 1H), 4.51 (d, *J* = 11.3 Hz, PhC*H*$_2$, 1H), 4.07 (dd, *J* = 9.6, 9.3 Hz, H-4, 1H), 3.92 (m, H-6a, H-6b, 2H), 3.57 (dd, *J* = 9.3, 3.1 Hz, H-3, 1H), 3.55 (m, H-5, 1H), 3.53 (s, OCH_3, 3H), 2.96 (s, OH-4, 1H). ^{13}C{^{1}H} NMR (150 MHz, $CDCl_3$): δ 166.1 (Ph*C*O), 138.3, 137.4, 133.1, 130.1, 130.0, 128.6, 128.5, 128.4, 128.2, 128.0, 127.7, 100.3 (C-1), 79.7 (C-3), 75.4 (C-5), 73.8 (Ph*C*H$_2$), 71.2 (Ph*C*H$_2$), 70.1 (C-6), 67.9 (C-2), 67.7 (C-4), 57.3 (O*C*H$_3$). HRMS (ESI-TOF) *m/z* [M + Na]$^+$: calcd for $C_{28}H_{30}O_7Na$, 501.1889; found, 557.1867.

A2. *2-Deoxy-2-phthalimido-3,4,6-tri-O-acetyl-α-D-glucopyranosyl trichloroacetimidate* (**G**). D-Glucosamine hydrochloride (**D**) (6.33 g, 29.2 mmol), Na_2CO_3 (3.10 g, 29.2 mmol) and phthalic anhydride (4.32 g, 29.2 mmol) were added to distilled water (38 mL). The mixture was stirred at rt overnight and concentrated to dryness. The residue was dissolved in pyridine (100 mL), and Ac_2O (40 mL, 423 mmol) was added. After stirring at rt for 12 h, the mixture was concentrated *in*

vacuo. The residue was dissolved in ethyl acetate, washed with distilled water, dried over anhydrous Na_2SO_4, and concentrated to give compound **E**. Compound **E** was dissolved in THF (100 mL) and benzylamine (3.82 mL, 35.0 mmol) was added at 0 °C. After stirring for 4 h at rt, the THF was removed *in vacuo*. The residue was dissolved in ethyl acetate, washed with 1 *N* aqueous HCl solution, saturated aqueous $NaHCO_3$ solution, and distilled water sequentially, and then dried over anhydrous Na_2SO_4. After concentration, crystallization from an ethyl acetate/hexane (3:1) mixed solvent afforded pure compound **F** (8.00 g, 18.4 mmol, 63%). Compound **F** (3.00 g, 6.89 mmol) was dissolved in CH_2Cl_2 (30 mL), and trichloroacetonitrile (2.00 mL, 19.9 mmol) and several drops of 1,8-diazabicyclo[5.4.0]undec-7-ene (DBU) were added. The reaction solution was stirred for 3 h at rt and concentrated *in vacuo*. Flash chromatography on silica gel gave trichloroacetimidate **G** (3.10 g, 5.35 mmol, 78%).[2,3]

B. Preparation of Disaccharide **5** (Scheme S2)

B1. *Methyl 2-Deoxy-2-phthalimido-3,4,6-tri-O-acetyl-β-D-glucopyranosyl-(1→4)-2-O-benzoyl-3,6-di-O-benzyl-β-D-mannopyranoside (**H**).* Trichloroacetimidate **G** (750 mg, 1.30 mmol) and methyl glycoside **C** (480 mg, 1.00 mmol) were dissolved in anhydrous CH_2Cl_2 (20 mL) after drying under high vacuum, and the solution was treated with molecular sieves (4 Å) (2.0 g). A

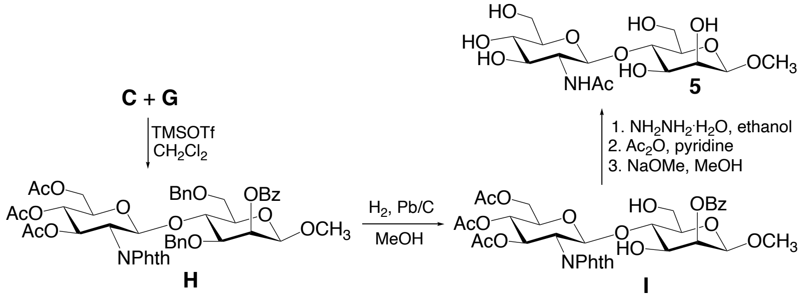

Scheme S2. Preparation of disaccharide **5** from **C** and **G** via **H** and **I**.

catalytic amount of TMSOTf (20 μL, 0.11 mmol) was added under a N_2 atmosphere at 0 °C. After 2 h, the reaction was quenched with the addition of a few drops of triethylamine and the molecular sieves were removed by filtration. The solution was concentrated to a syrup *in vacuo*, and the residue was purified by flash chromatography on silica gel to afford disaccharide **H** (790 mg, 0.88 mmol, 88%). **H**: ^{1}H NMR (600 MHz, $CDCl_3$): δ 8.03–7.10 (m, 19H), 5.77 (dd, *J* = 3.2, 0.9 Hz, H-2, 1H), 5.70 (d, *J* = 8.5 Hz, H-1′, 1H), 5.68 (dd, *J* = 10.6, 9.1 Hz, H-3′, 1H), 5.11 (dd, *J* = 10.6, 9.5 Hz, H-4′, 1H), 4.91 (d, *J* = 11.9 Hz, PhCH_2, 1H), 4.60 (d, *J* = 11.9 Hz, PhCH_2, 1H), 4.40 (d, *J* = 0.9 Hz, H-1, 1H), 4.36 (d, *J* = 12.0 Hz, PhCH_2, 1H), 4.30–4.26 (m, H-4, H-2′, 2H) 4.23 (d, *J* = 12.0 Hz, PhCH_2, 1H), 3.95 (dd, *J* = 12.3, 3.8 Hz, H-6a′, 1H), 3.78 (dd, *J* = 9.0, 3.2 Hz, H-3, 1H), 3.74 (dd, *J* = 12.3, 2.0 Hz, H-6b′, 1H), 3.55 (dd, *J* = 11.5, 4.0 Hz, H-6a, 1H), 3.51 (dd, *J* = 11.5, 1.7 Hz, H-6b, 1H), 3.42 (m, H-5, 1H), 3.40 (s, OCH_3, 3H), 3.35 (m, H-5′, 1H). 1.94 (s, COCH_3, 3H), 1.92 (s, COCH_3, 3H), 1.80 (s, COCH_3, 3H). ^{13}C{^{1}H} NMR (150 MHz, $CDCl_3$): δ 170.6 (*C*OCH$_3$), 170.1 (*C*OCH$_3$), 169.4 (*C*OCH$_3$), 165.9 (Ph*C*O), 138.4–126.6, 100.1 (C-1), 98.2 (C-1′), 78.6 (C-3), 75.1

(C-5), 74.2 (C-4), 72.9 (Ph*C*H_2), 71.6 (C-5′), 70.8 (C-3′), 70.7 (Ph*C*H_2), 68.5 (C-4′), 68.2 (C-2), 68.0 (C-6), 61.3 (C-6′), 57.0 (O*C*H_3), 55.3 (C-2′), 20.7 (CO*C*H_3), 20.6 (CO*C*H_3), 20.4 (CO*C*H_3). HRMS (ESI-TOF) *m/z* $[M + Na]^+$: calcd for $C_{48}H_{49}NO_{16}Na$, 918.2944; found, 918.2818.

B2. *Methyl 2-Deoxy-2-phthalimido-3,4,6-tri-O-acetyl-β-D-glucopyranosyl-(1→4)-2-O-benzoyl-β-D-mannopyranoside (**I**).* Compound **H** (750 mg, 0.84 mmol) was dissolved in methanol (20 mL) and treated with Pd/C (10%, 200 mg) and H_2 overnight. The Pd/C catalyst was removed by filtration and the filtrate was concentrated to dryness *in vacuo* to afford **I** (580 mg, 0.81 mmol, 96%). **I**: ^{1}H NMR (600 MHz, $CDCl_3$): δ 8.03–7.39 (m, 9H), 5.79 (dd, J = 10.6, 9.0 Hz, H-3′, 1H), 5.68 (dd, J = 3.3, 0.9 Hz, H-2, 1H), 5.51 (d, J = 8.5 Hz, H-1′, 1H), 5.05 (dd, J = 10.2, 9.2 Hz, H-4′, 1H), 4.48 (d, J = 0.9 Hz, H-1, 1H), 4.34 (dd, J = 10.6, 8.5 Hz, H-2′, 1H), 4.15 (dd, J = 12.2, 2.3 Hz, H-6a′, 1H), 4.08 (dd, J = 12.2, 7.2 Hz, H-6b′, 1H), 4.00 (ddd, J = 10.2, 7.2, 2.2 Hz, H-5′, 1H), 3.96 (dd, J = 9.3, 9.1 Hz, H-4, 1H), 3.85 (dd, J = 9.1, 3.4 Hz, H-3, 1H), 3.45 (dd, J = 12.1, 2.1 Hz, H-6a, 1H), 3.38 (s, OCH_3, 3H), 3.25 (ddd, J = 9.3, 3.7, 2.1 Hz, H-5, 1H), 3.20 (dd, J = 12.1, 3.7 Hz, H-6b, 1H), 2.00 (s, $COCH_3$, 3H), 1.83 (s, $COCH_3$, 3H), 1.77 (s, $COCH_3$, 3H). $^{13}C\{^1H\}$ NMR (150 MHz, $CDCl_3$): δ 170.7 (*C*OCH_3), 170.1 (*C*OCH_3), 169.6 (*C*OCH_3), 165.8 (Ph*C*O), 133.1, 130.1, 130.0, 128.4, 100.6 (C-1), 99.0 (C-1′), 79.3 (C-4), 74.5 (C-5), 72.0 (C-5′), 71.2 (C-3), 70.6 (C-2), 70.4 (C-3′), 69.0 (C-4′), 62.1 (C-6′), 60.9 (C-6), 57.4 (O*C*H_3), 54.7 (C-2′), 20.7 (CO*C*H_3), 20.5 (CO*C*H_3), 20.2 (CO*C*H_3). HRMS (ESI-TOF) *m/z* $[M + Na]^+$: calcd for $C_{34}H_{37}NO_{16}Na$, 738.2010; found, 738.1913.

B3. *Methyl 2-Acetamido-2-deoxy-β-D-glucopyranosyl-(1→4)-β-D-mannopyranoside (**5**).* Compound **I** (100 mg, 0.140 mmol) dissolved in ethanol (10 mL) and hydrazine hydrate (1.50 mL) was added. After refluxing for 20 h, the reaction mixture was concentrated *in vacuo* to a syrup, which was dried under high vacuum. The dried residue was dissolved in pyridine (10 mL) and Ac_2O (2.00 mL) was added. The mixture was stirred at rt overnight and concentrated *in vacuo*. The residue was purified by flash chromatography on silica gel to give an acetylated disaccharide, which was treated with sodium methoxide in methanol (20 mL, pH > 10) overnight. Final product (**5**) was dissolved in ~0.5 mL of distilled water, and the solution was applied to a column (2.5 x 100 cm) containing Bio-gel P2 gel-filtration resin (45–90 μm). The column was eluted with distilled water at ~1.5 mL/min, and fractions (~10 mL) were collected. Fractions containing pure product were collected and concentrated at 30 °C *in vacuo* to give **5** as a white solid (43 mg, 0.109 mmol, 78%). See Tables S1 and S2 for ^{1}H and ^{13}C chemical shifts, and NMR spin-couplings in **5**. HRMS (ESI-TOF) *m/z* $[M + Na]^+$: calcd for $C_{15}H_{26}NO_{11}Na$, 419.1405; found, 419.1391.

C. *Preparation of Disaccharides $\mathbf{5}^{1'}$ and $\mathbf{5}^{2'}$*. ^{13}C-Labeled Disaccharides $\mathbf{5}^{1'}$ and $\mathbf{5}^{2'}$ were prepared using the route described above but substituting either D-[1-^{13}C]glucosamine or D-[2-^{13}C]glucosamine for unlabeled D-glucosamine (Scheme S1) in the protocol. The singly ^{13}C-labeled D-glucosamines were obtained from Omicron Biochemicals, Inc. (South Bend, IN). See Table S3 for intra-residue J_{CC} values in $\mathbf{5}^{1'}$ and $\mathbf{5}^{2'}$.

References

1. H. Qin and T. B. Grindley, *J. Carbohydr. Chem.,* 1994, **13,** 475–490.
2. Y. Du, M. Zhang and F. Kong, *Tetrahedron,* 2001, **57**, 1757–1763.
3. R. R. Schmidt and J. Michel, *Angew. Chem., Int. Ed. Engl.*, 1980, **19**, 731–732.

Table S1. ^{1}H and ^{13}C Chemical Shifts[a] in Disaccharide **5**.

**compound/ residue**	**^{1}H chemical shifts (ppm)**								
	H1	H2	H3	H4	H5	H6a	H6b	OCH_3	$COCH_3$
βMan	4.555	4.007	3.729	3.672	3.417	3.833	3.650	3.514	
βGlcNAc	4.524	3.728	3.544	3.441	3.498	3.920	3.729		2.051
**compound/ residue**	**^{13}C chemical shifts (ppm)**								
	C1	C2	C3	C4	C5	C6	OCH_3	$\textit{C}OCH_3$	$CO\textit{C}H_3$
βMan	103.57	72.36	74.41	80.18	77.43	63.17	59.50		
βGlcNAc	104.27	58.24	76.11	72.45	78.57	63.28		177.27	24.79

[a]In $^{2}H_2O$ at 22 °C. Chemical shifts are given in ppm relative to external DSS; ± 0.001 ppm for ^{1}H, ± 0.01 ppm for ^{13}C. H6a is defined as the less shielded H6 hydrogen.

Table S2. Intra-Residue ^{1}H-^{1}H Spin-Coupling Constants[a] in Disaccharide **5**.

**compound/ residue**	**^{1}H-^{1}H spin-coupling constants (Hz)**						
	$^{3}J_{H1,H2}$	$^{3}J_{H2,H3}$	$^{3}J_{H3,H4}$	$^{3}J_{H4,H5}$	$^{3}J_{H5,H6a}$	$^{3}J_{H5,H6b}$	$^{2}J_{H6a,H6b}$
βMan	1.0	3.2	9.4	9.6	2.2	6.1	–12.1
βGlcNAc	8.5	10.4	8.8	9.7	2.3	6.1	–12.4

[a]In Hz ± 0.1 Hz, $^{2}H_2O$ at 22 °C. H6a is defined as the less shielded H6 hydrogen; $^{2}J_{HH}$ values were assumed to have negative signs.

Table S3. Intra-Residue ^{13}C-^{13}C Spin-Coupling Constants in Disaccharides **5**[1'] and **5**[2'].

**compound/ residue**	**^{13}C-^{13}C spin-coupling constants (Hz)**					
	$^{1}J_{C1,C2}$	$^{2}J_{C1,C3}$	$^{2}J_{C1,C5}$	$^{3}J_{C1,C6}$	$^{1}J_{C2,C3}$	$^{2}J_{C2,C4}$
βGlcNAc	45.5	+4.7	0	4.0	37.1	+2.4

[a]In Hz ± 0.1 Hz, $^{2}H_2O$ at 22 °C. H6a is defined as the less shielded H6 hydrogen.

Representative ^{1}H and $^{13}C\{^{1}H\}$ NMR spectra of Compounds C, H and I

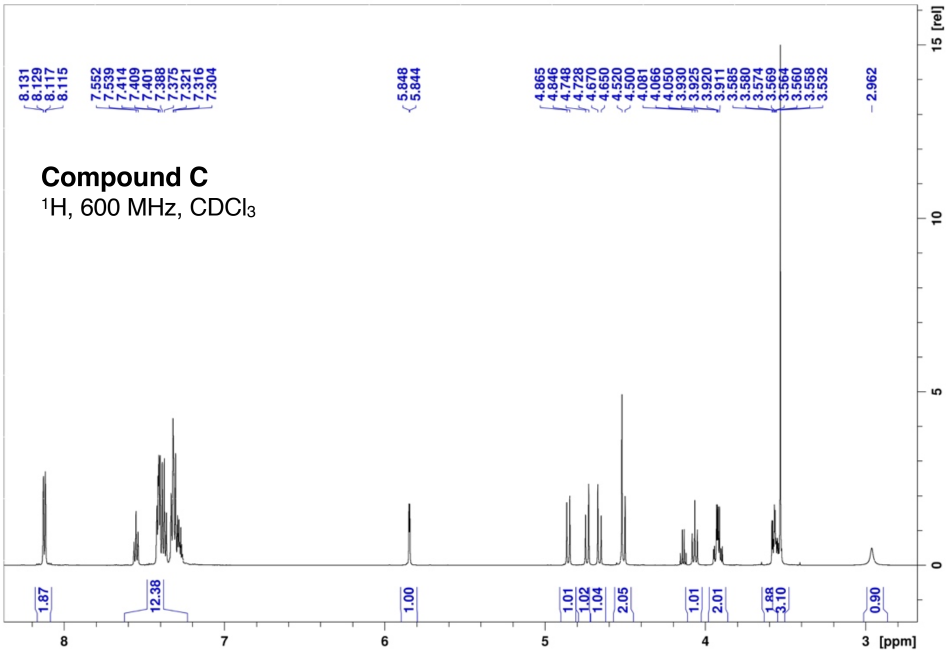

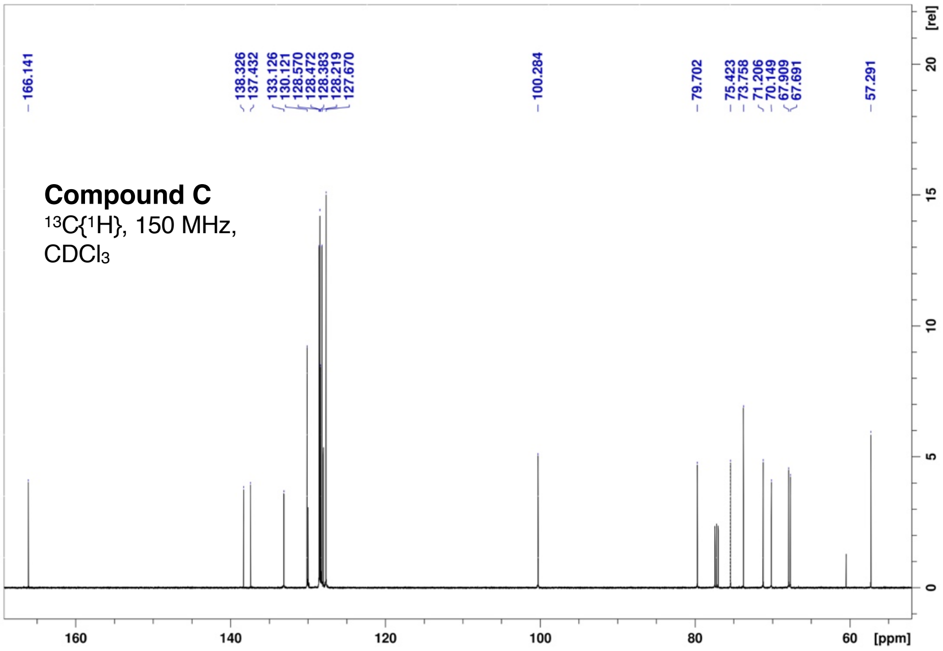

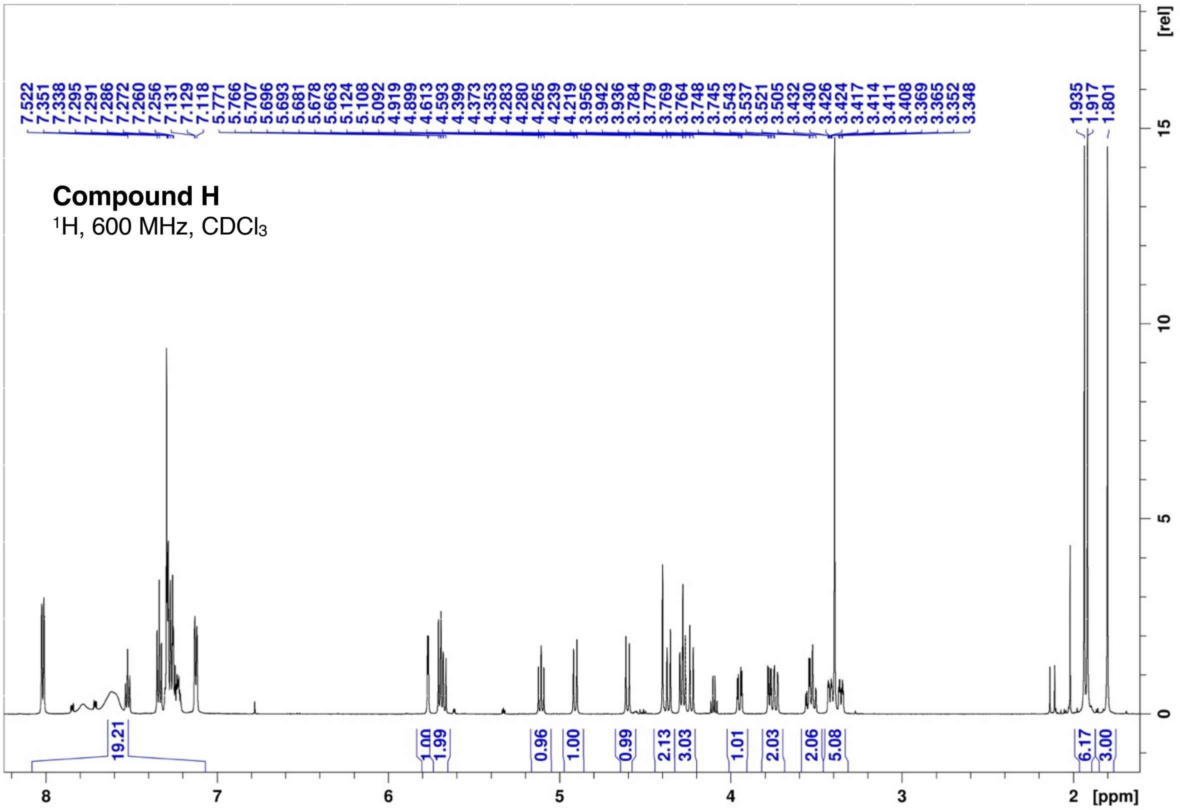
7.522
7.351
7.338
7.295
7.291
7.286
7.272
7.260
7.256
7.131
7.129
7.118
5.771
5.766
5.707
5.696
5.693
5.681
5.678
5.663
5.124
5.108
5.092
4.919
4.899
4.613
4.593
4.399
4.373
4.353
4.283
4.280
4.265
4.239
4.219
3.956
3.942
3.936
3.784
3.779
3.769
3.764
3.748
3.745
3.543
3.537
3.521
3.505
3.432
3.430
3.426
3.424
3.417
3.414
3.411
3.408
3.369
3.365
3.352
3.348
1.935
1.917
1.801
Compound H
¹H, 600 MHz, CDCl₃
19.21
1.00
1.99
0.96
1.00
0.99
2.13
3.03
1.01
2.03
2.06
5.08
6.17
3.00
[rel]
[ppm]

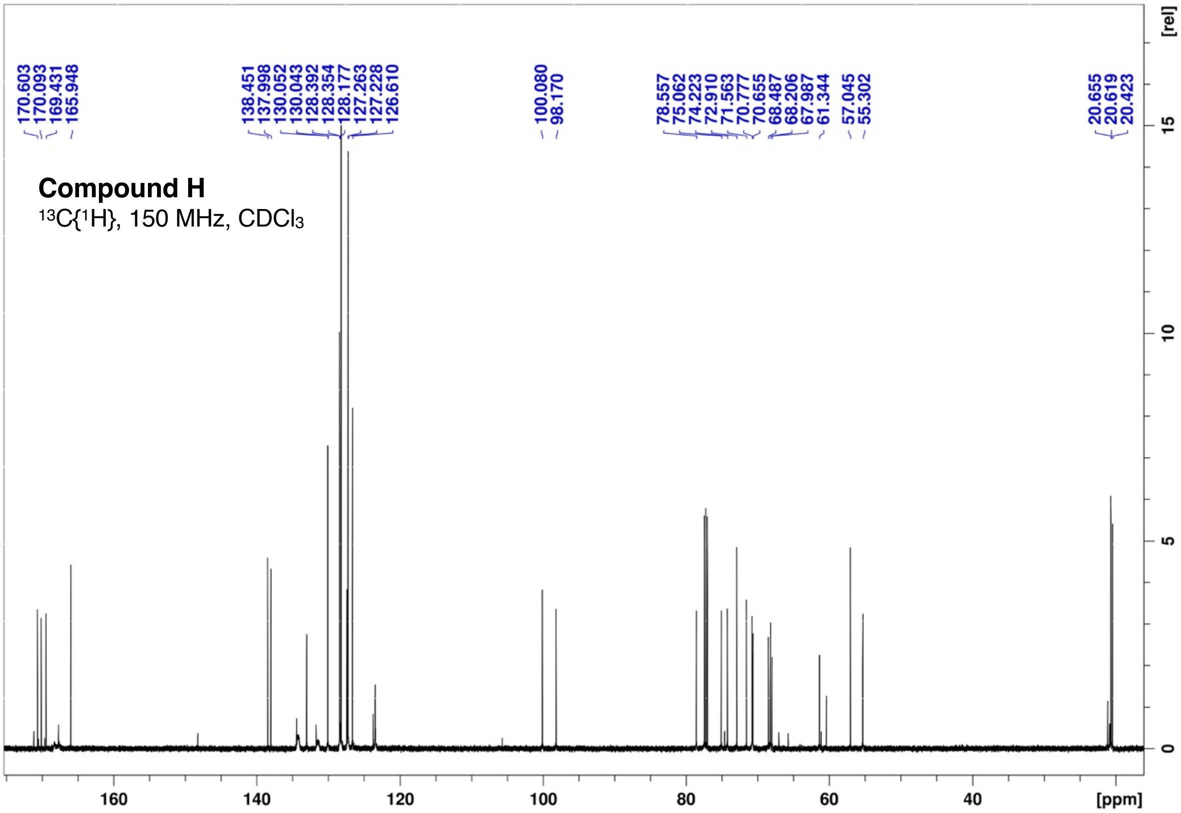
170.603
170.093
169.431
165.948
138.451
137.998
130.052
130.043
128.392
128.354
128.177
127.263
127.228
126.610
100.080
98.170
78.557
75.062
74.223
72.910
71.563
70.777
70.655
68.487
68.206
67.987
61.344
57.045
55.302
20.655
20.619
20.423
Compound H
¹³C{¹H}, 150 MHz, CDCl₃
[rel]
[ppm]

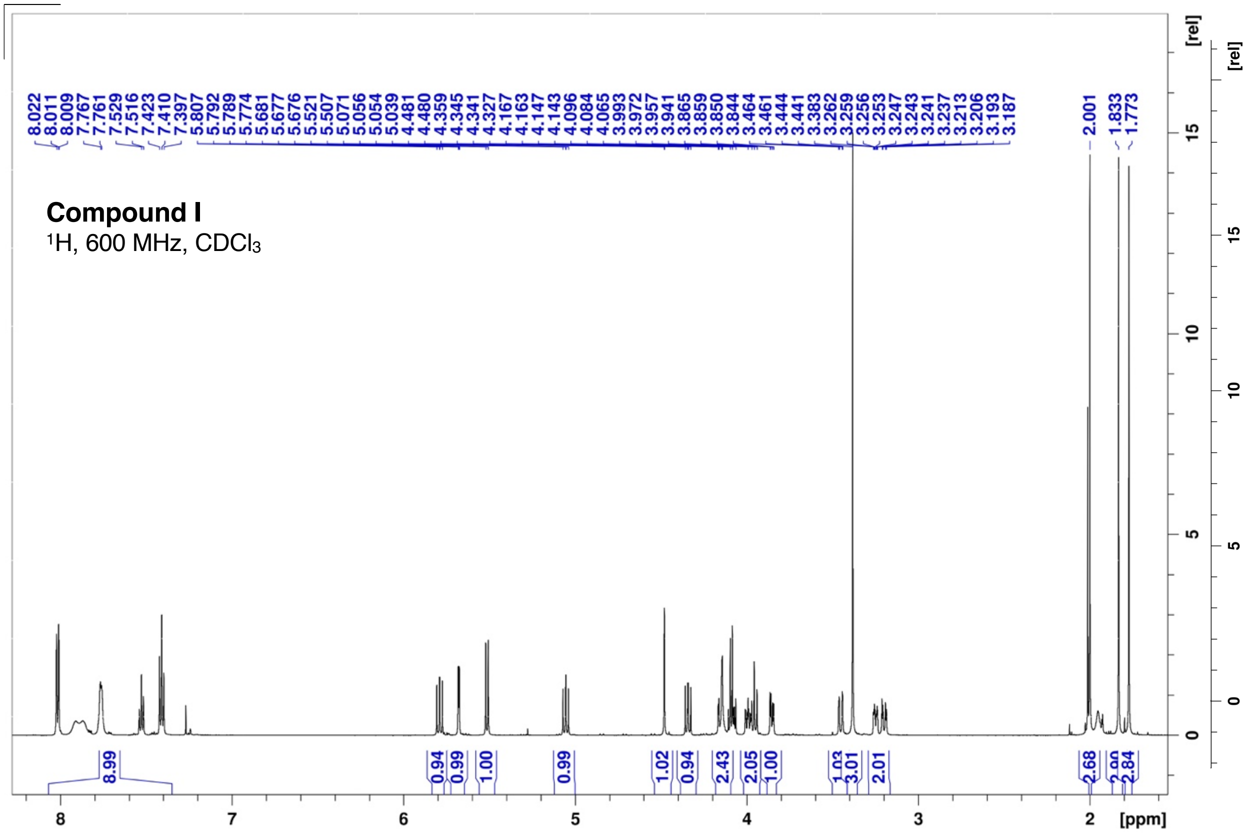
Compound I
¹H, 600 MHz, CDCl3

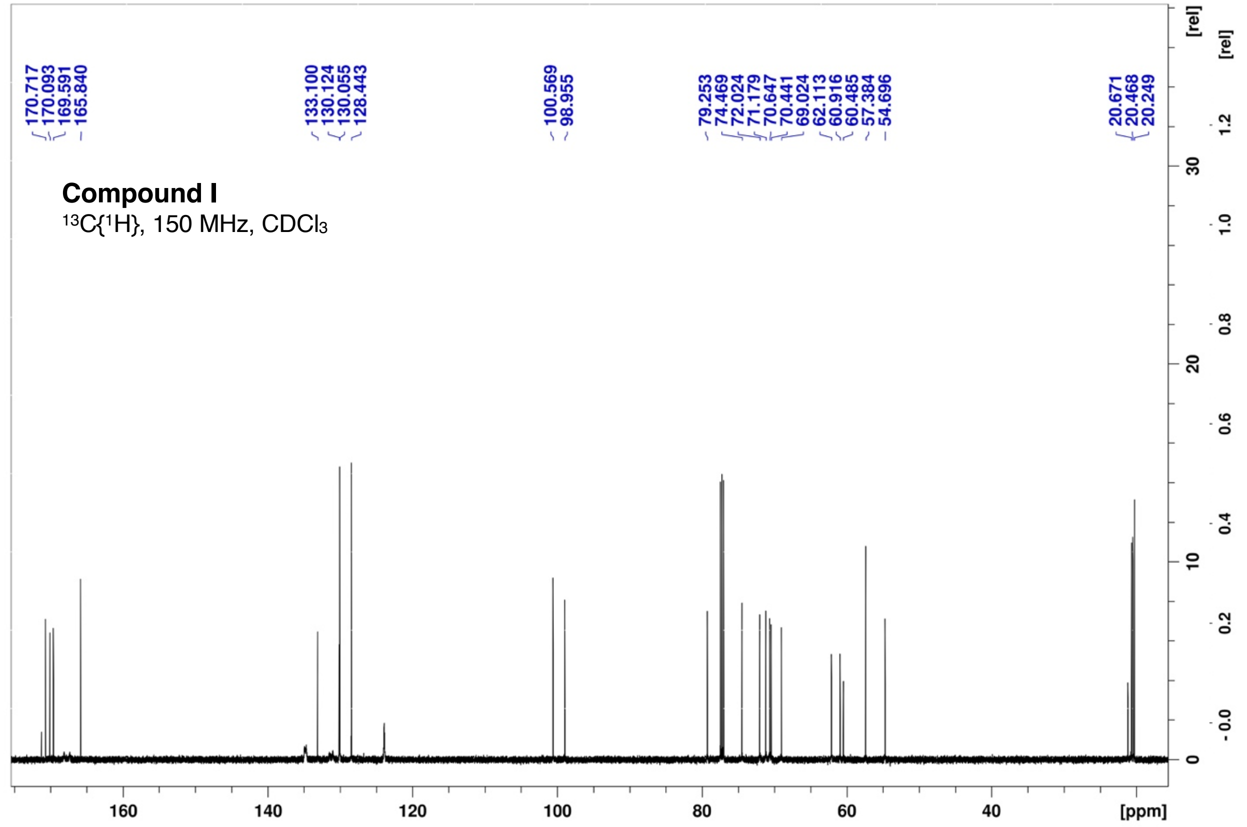
Compound I
¹³C{¹H}, 150 MHz, CDCl3

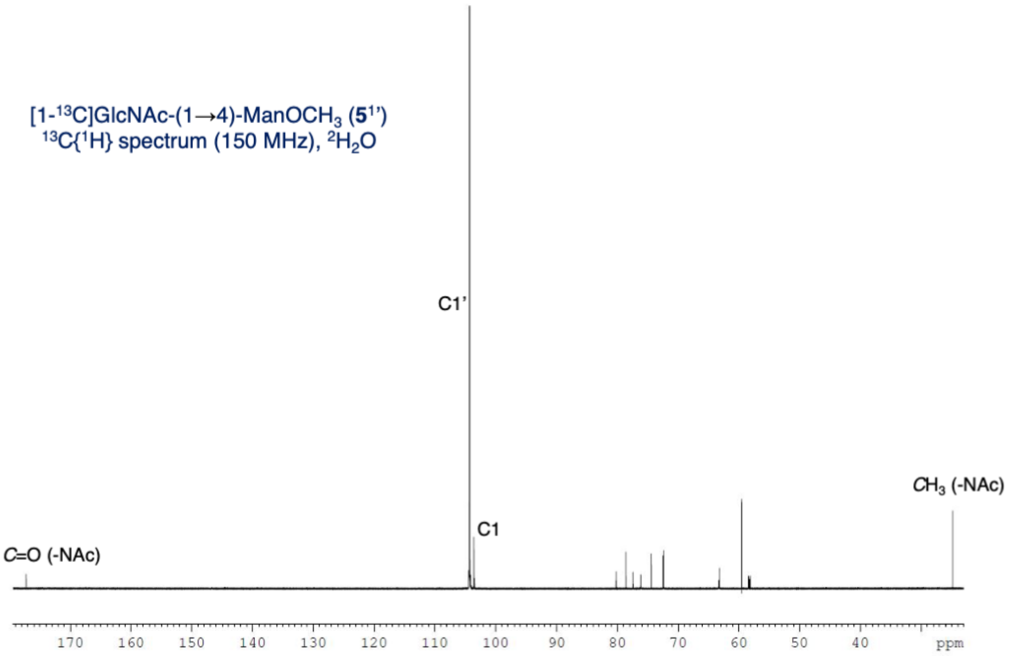

Figure S1. Full $^{13}C(^{1}H\}$ NMR spectrum of **5**$^{1'}$ showing signal assignments for the anomeric carbons and the carbons of the *N*-acetyl side-chain.

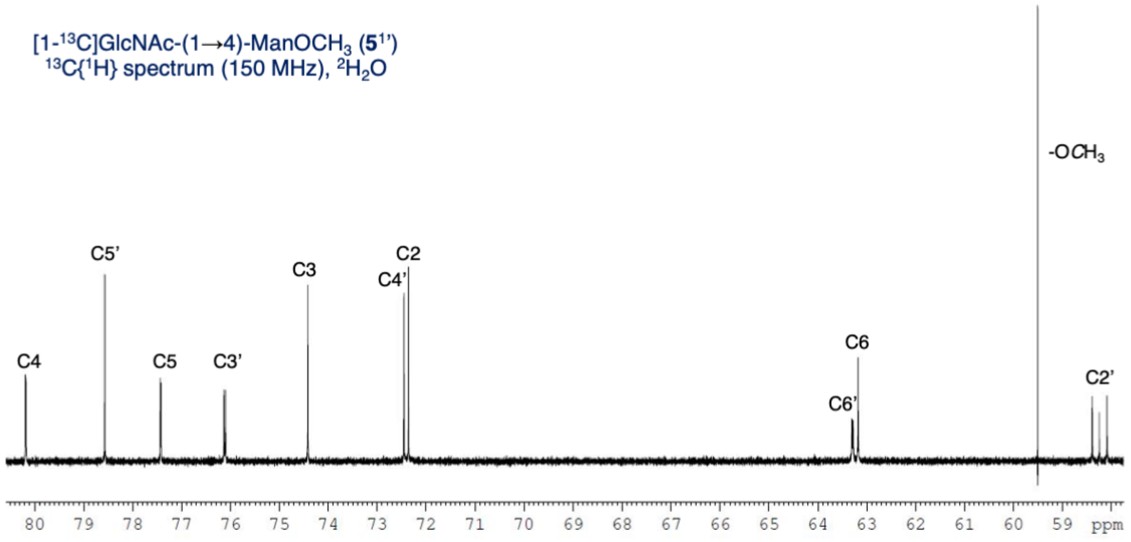

Figure S2. Expanded 58–80 ppm region of the ^{13}C(^{1}H} NMR spectrum of **5**$^{1'}$ in Figure S1 showing signal assignments for the natural abundance non-anomeric carbons and the aglycone methyl carbons.

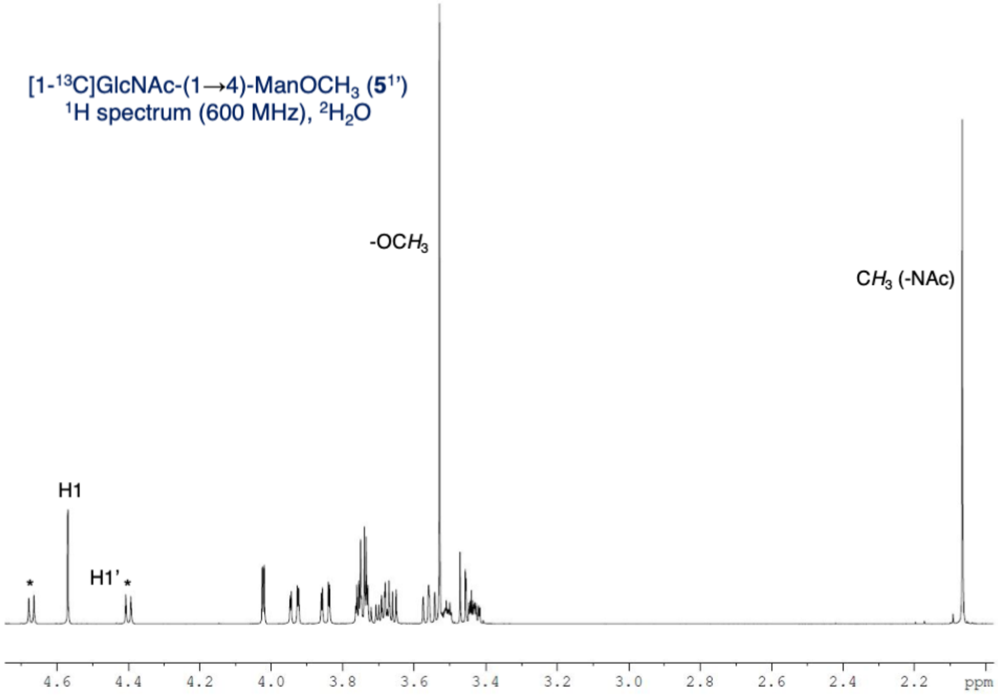

Figure S3. Full ^{1}H NMR spectrum of **5**$^{1'}$ showing signal assignments for the anomeric hydrogens and the methyl hydrogens of the *N*-acetyl side-chain and aglycone.

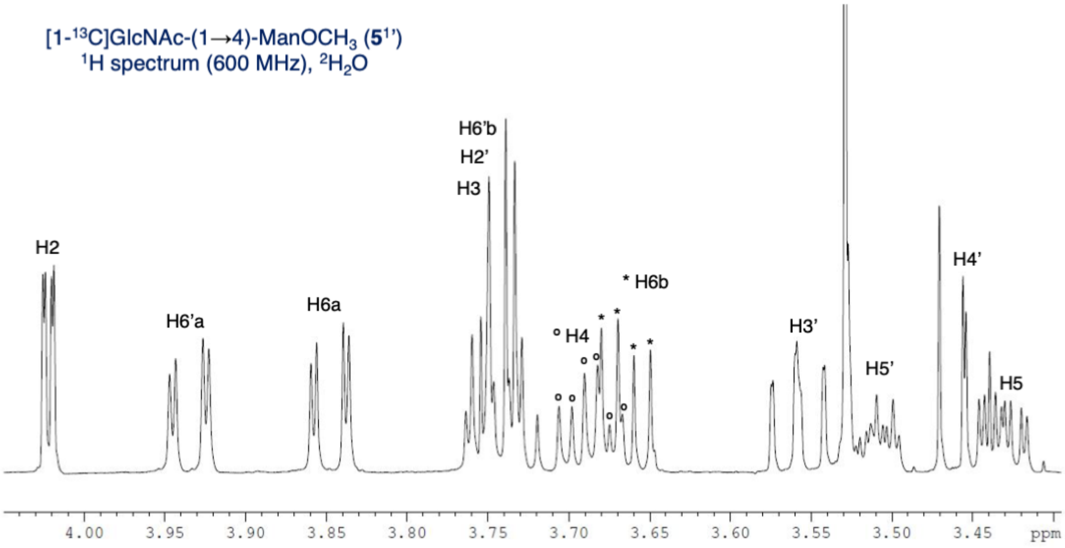

Figure S4. Expanded 3.40–4.05 ppm region of the ^{1}H NMR spectrum of **5**$^{1'}$ in Figure S3 showing signal assignments for the non-anomeric hydrogens.

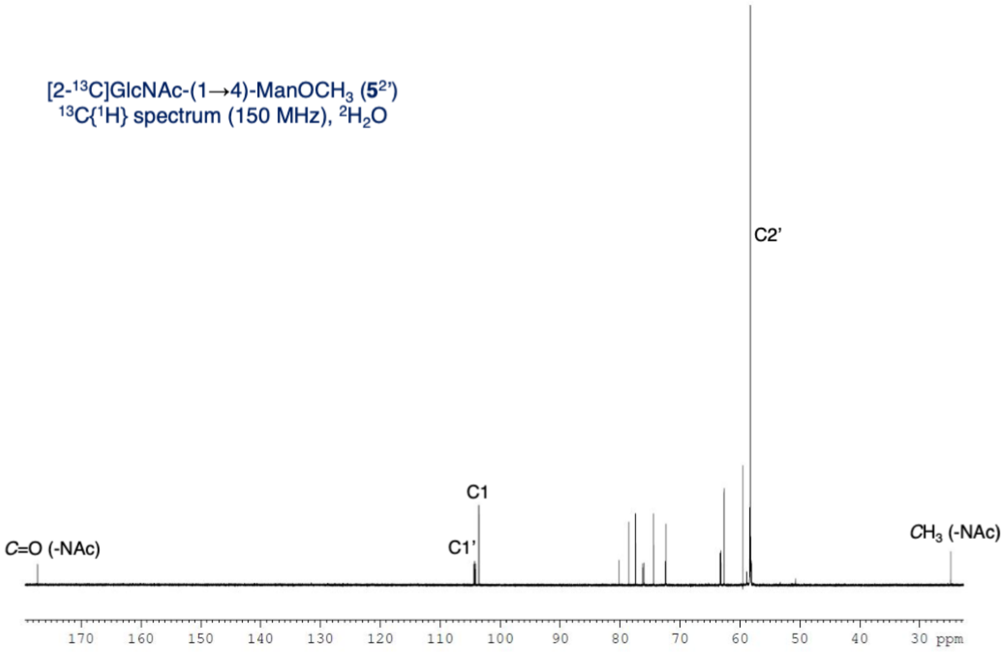

Figure S5. Full $^{13}C(^{1}H\}$ NMR spectrum of $\mathbf{5}^{2'}$ showing signal assignments for the labeled C2' carbon, and the natural abundance anomeric carbons and the carbons of the *N*-acetyl side-chain.

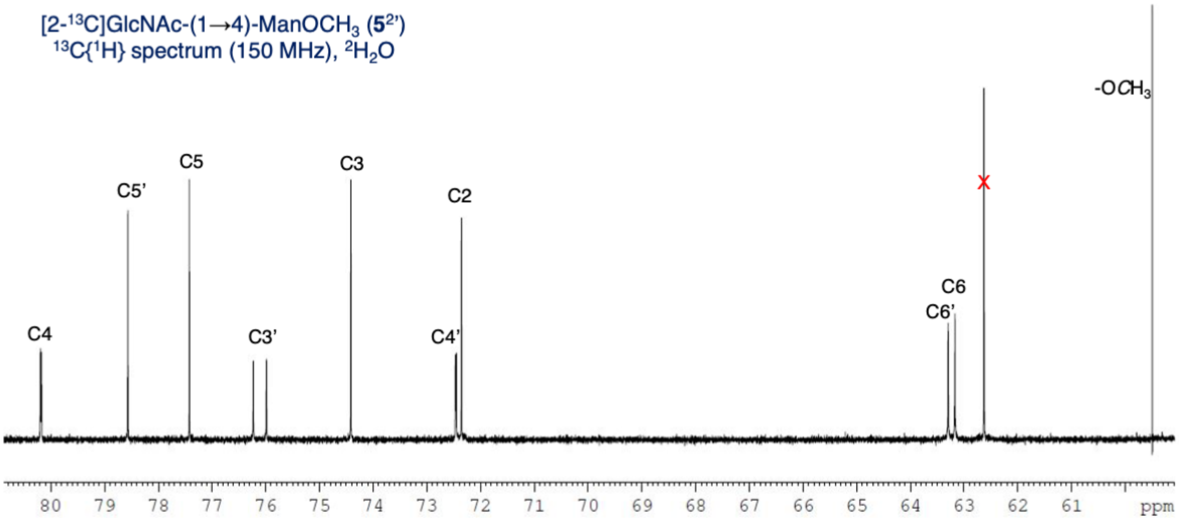

Figure S6. Expanded 58–80 ppm region of the $^{13}C\{^{1}H\}$ NMR spectrum of $\mathbf{5}^{2'}$ in Figure S5 showing signal assignments for the natural abundance non-anomeric carbons and the aglycone methyl carbons.

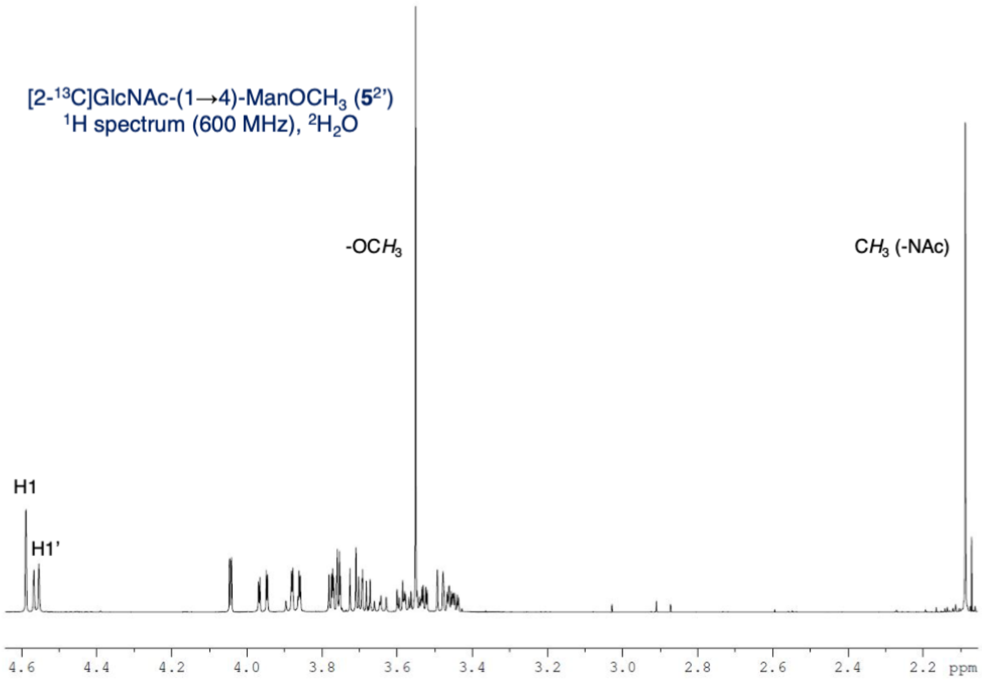

Figure S7. Full ^{1}H NMR spectrum of **5**$^{2'}$ showing signal assignments for the anomeric hydrogens and the methyl hydrogens of the *N*-acetyl side-chain and aglycone.

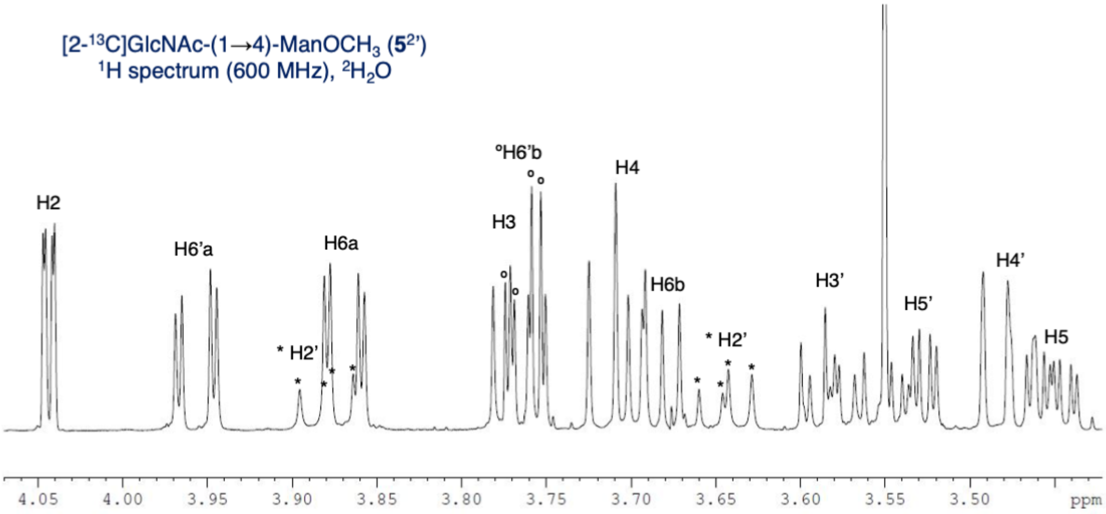

Figure S8. Expanded 3.40–4.05 ppm region of the ^{1}H NMR spectrum of $\mathbf{5}^{2'}$ in Figure S7 showing signal assignments for the non-anomeric hydrogens.

Preparation of Disaccharide 6[1',2]

(Methyl 2-Acetamido-2-deoxy-β-D-[1-^{13}C]glucopyranosyl-(1→2)-α-D-[2-^{13}C]mannopyranoside)
(Scheme S1)

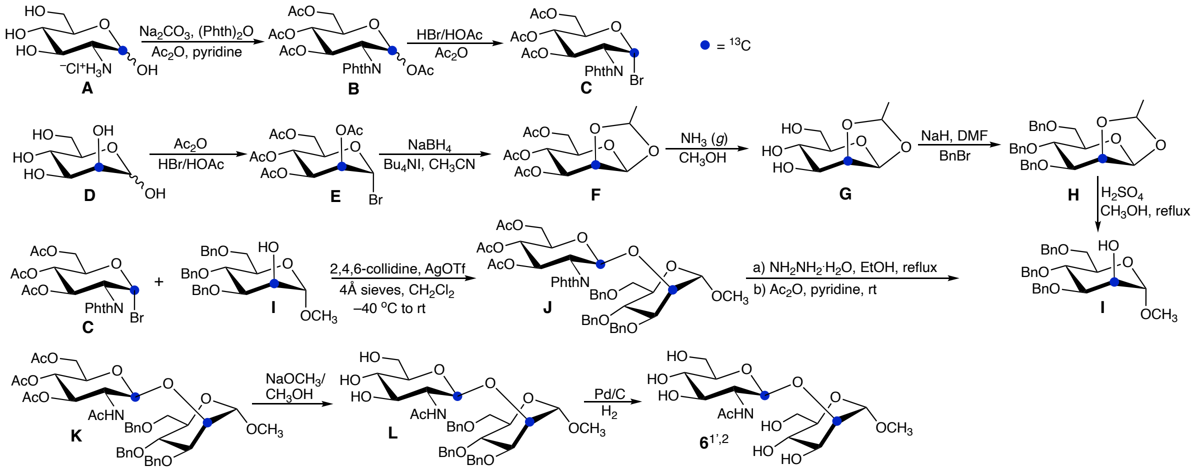

Scheme S1. Chemical route to prepare disaccharide **6**[1',2].

1. *1,3,4,6-Tetra-O-acetyl-2-deoxy-2-phthalimido-α/β-D-glucopyranose (**B**α and **B**β).* D-Glucosamine hydrochloride (**A**) (1.43 g, 6.61 mmol), Na_2CO_3 (0.830 g, 6.63 mmol) and phthalic anhydride (0.982 g, 6.63 mmol) were dissolved in distilled water (8.6 mL) and the solution was stirred at rt for 20 h.[1] The mixture was evaporated to dryness to give crude 2-deoxy-2-phthalimido-β-D-glucopyranose, which was dissolved in acetic anhydride (11.0 mL, 116 mmol) and dry pyridine (22.0 mL). The solution was stirred at rt for 24 h. The reaction mixture was then diluted with CH_2Cl_2 (30 mL) and poured into ice water (30 mL). The solution was extracted twice with 25 mL of CH_2Cl_2, and the combined organic extracts were washed with cold distilled water, 1 *M* aqueous HCl, saturated aqueous $NaHCO_3$, and cold distilled water again. Solvent removal *in vacuo* afforded a yellow foam-like solid, which contained a mixture of **B**α and **B**β. This mixture was used in the next step without further purification.

2. *3,4,6-Tri-O-acetyl-2-deoxy-2-phthalimido-α-D-glucopyranosyl bromide (**C**).* The mixture of **B**α and **B**β from (A) was dissolved in acetic anhydride (2.0 mL, 21 mmol), and solution was cooled to 0 °C in an ice-bath. A solution of HBr in glacial acetic acid (2 mL, 33 wt. %, 138 mmol) was then added to the cooled solution. The reaction mixture was stirred in an ice bath for 1 h, followed by stirring at rt for an additional 22 h.[1] The mixture was then diluted with CH_2Cl_2, chilled with ice, and washed with cold distilled water three times and saturated aqueous $NaHCO_3$ once. The organic layer was concentrated *in vacuo* and the residue was purified by flash chromatography on a silica gel column (eluent: ethyl acetate/hexanes, 1:2), affording glycosyl bromide **C** (2.95 g, 5.92 mmol, 89.6% yield from two steps).

3. *3,4,6-Tri-O-acetyl-1,2-O-ethylidene-β-D-mannopyranose (**F**).* D-Mannose (**D**) (1.91 g, 10.6 mmol) was added to a round-bottom flask immersed in an ice bath. Acetic anhydride (10.0 mL) and hydrobromic acid (33 wt. % solution in glacial HOAc) (2.0 mL) was then added. The reaction mixture was stirred for 10 min until all of the D-mannose had dissolved. Another portion of hydrobromic acid (33 wt. % solution in glacial HOAc) (10.0 mL) was then added dropwise and

the reaction mixture was stirred for 23 h at rt.[2] The mixture was concentrated *in vacuo* to dryness to afford 2,3,4,6-tetra-*O*-acetyl-α-D-mannopyranosyl bromide (**E**) (4.37 g, 10.6 mmol).

Sodium borohydride (0.72 g, 19.0 mmol) and tetra-*n*-butylammonium iodide (1.72 g, 4.66 mmol) were then added to the flask containing **E** (4.37 g, 10.6 mmol), anhydrous acetonitrile (30.0 mL) was added, and the reaction mixture was stirred overnight.[3] The reaction mixture was diluted with CH_2Cl_2 (30 mL), washed with cold distilled water, and extracted with CH_2Cl_2 (25 mL) three times. The organic extracts were combined and concentrated *in vacuo*, and the residue was purified by flash chromatography on a silica gel column (eluent: ethyl acetate/hexane, 1:1) to afford compound **F** (3.21 g, 9.63 mmol, 90.9% yield from two steps).

4. *3,4,6-Tri-O-benzyl-1,2-O-ethylidene-β-D-mannopyranose (**H**).* Compound **F** (3.21 g, 9.63 mmol) was dissolved in methanol (35 mL) and the solution was saturated with NH_3. The reaction mixture was stirred for 22 h and then concentrated *in vacuo* to dryness to afford crude 1,2-*O*-ethylidene-β-D-mannopyranose (**G**). Crude **G** was dissolved in DMF (30 mL) and NaH (95% dispersion, 3.60 g, 143 mmol) was added to the solution. The mixture was stirred at rt for 1 h. Benzyl bromide (13 mL, 109 mmol) was then added dropwise at 0 ^oC and the mixture was stirred at 0 ^oC for 1 h. After stirring at rt for an additional 23 h, the mixture was washed with distilled water, filtered and the filtrate extracted with CH_2Cl_2 (25 mL) three times. The organic extracts were combined and dried over anhydrous Na_2SO_4, evaporated to dryness *in vacuo*, and the crude product was purified by flash chromatography on a silica gel column (eluent: ethyl acetate/hexanes, 1:2) to afford compound **H** (3.19 g, 6.69 mmol, 69.5% yield from two steps).

5. *Methyl 3,4,6-Tri-O-benzyl-α-D-mannopyranoside (**I**).* Compound **H** (3.19 g, 6.69 mmol) was dissolved in anhydrous CH_3OH (40.0 mL) and H_2SO_4 (98%, 1.84 g/mL) was added dropwise to adjust the solution pH to ~2. The reaction mixture was stirred under reflux for ~24 h, at the end of which time TLC (1:1 ethyl acetate/hexanes) indicated that most of the reactant had been converted to product.[4] The solution was washed with $NaHCO_3$ until the wash remained basic, and then was concentrated *in vacuo* to a syrup. The syrup was purified by flash chromatography on a silica gel column (eluent: ethyl acetate/hexane, 1:2) to afford compound **I** (2.15 g, 4.62 mmol, 69.0%).

6. *Methyl 2-O-(3,4,6-O-Acetyl-2-deoxy-2-phthalimido-β-D-glucopyranosyl)-3,4,6-tri-O-benzyl-α-D-mannopyranoside (**J**).* Compound **I** (1.46 g, 3.14 mmol) was dissolved in anhydrous CH_2Cl_2 (20.0 mL) in a round-bottom flask, and 2,4,6-trimethylpyridine (1.14 mL, 8.56 mmol), silver triflate (2.30 g, 8.95 mmol) and 4 Å molecular sieves (3.0 g) were added. Compound **C** (2.20 g, 4.42 mmol) was dissolved in anhydrous CH_2Cl_2 (20.0 mL) and added to the flask dropwise. The reaction mixture was stirred under a N_2 atmosphere in a –80 ^oC dry-ice/acetone bath for 24 h.[5,6] The reaction mixture was then diluted with CH_2Cl_2 (20.0 mL) and filtered to remove solids. The filtrate was washed with cold distilled water, 1 *M* aqueous HCl, saturated aqueous $NaHCO_3$, and cold distilled water. The organic layer was dried over anhydrous Na_2SO_4 and concentrated *in vacuo* to dryness to afford crude disaccharide **J**, which was used in the next step without further purification.

7. *Methyl 2-O-(3,4,6-O-acetyl-2-deoxy-2-acetamido-β-D-glucopyranosyl)-3,4,6-tri-O-benzyl-α-D-mannopyranoside (**K**).* Crude product **J** was dissolved in ethanol (60.0 mL) and hydrazine monohydrate (8.00 mL, 164 mmol) was added to the solution. The reaction mixture

was refluxed for 3 h and the solvent was then removed *in vacuo*. The resulting residue was dissolved in pyridine (40.0 mL) and acetic anhydride (20.0 mL, 212 mmol), and the solution was stirred at rt for 24 h. The reaction mixture was concentrated *in vacuo* until no more liquid could be removed. The remaining solution was diluted with CH_2Cl_2 and washed with cold distilled water, 1 *M* aqueous HCl, saturated aqueous $NaHCO_3$ and cold distilled water again. The organic layer was concentrated *in vacuo* and the crude product was purified by flash chromatography on a silica gel column (eluent: ethyl acetate/hexane, 2:1) to afford compound **K** (0.760 g, 0.956 mmol, 30.4% yield from two steps).

8. *Methyl 2-O-(2-acetamido-2-deoxy-α-D-glucopyranosyl)-α-D-mannopyranoside (**6**)*. Compound **K** (740 mg, 0.931 mmol) was dissolved in methanol (40.0 mL), and sodium methoxide in methanol (25 wt. %) was added dropwise to adjust the solution to ~ pH 10. The reaction mixture was stirred at rt for 14 h, and then neutralized with batchwise addition of Dowex 50 x 8 ion-exchange resin in the H^+ form. The resin was removed by filtration and the filtrate was concentrated *in vacuo* to dryness. The crude product **L** was dissolved in ethanol (25 mL) and treated with Pd/C (640 mg) and H_2 overnight. The solution was then filtered and concentrated *in vacuo* to remove the solvent. The crude product was purified by flash chromatography on a silica gel column (eluent: CH_2Cl_2/CH_3OH, 2:1) to afford disaccharide **6** (310 mg, 0.777 mmol, 83.4% yield from two steps). ^1H and ^{13}C chemical shifts, and ^1H-^1H spin-couplings, for **6** are given in Tables S1 and S2, respectively.

9. *Preparation of Disaccharide* $\mathbf{6}^{1',2}$. The route described above (Scheme S1) was used to prepare $\mathbf{6}^{1',2}$ by substituting **A** with D-[1-^{13}C]glucosamine hydrochloride and **D** with D-[1-^{13}C]mannose. The singly ^{13}C-labeled monosaccharides were obtained from Omicron Biochemicals, Inc. (South Bend, IN). ^{13}C-^{13}C and ^{13}C-^1H spin-couplings measured in $\mathbf{6}^{1',2}$ are given in Table S2, and representative ^1H and $^{13}C\{^1H\}$ NMR spectra are shown in Figures S1 and S2, respectively.

<u>References</u>

1. R. U. Lemieux, T. Takeda and B. Y. Chung, *Synthetic Methods for Carbohydrates*. El Khadem, H. S. Editor, *ACS Symposium Series*, 1976, **39**, 90–115.
2. K. P. R. Kartha and H. J. Jennings, *J. Carbohydr. Chem.,* 1990, **9**, 777–781.
3. T. Mori, K. Hatano, K. Matsuoka, Y. Esumi, E. J. Toone and D. Terunuma, *Tetrahedron*, 2005, **61**, 2751–2760.
4. J. Ning and F. Kong, *Carbohydr. Res.*, 2001, **330**, 165–175.
5. I. Brockhausen, G. Moller, J. Yang, S. H. Khan, K. L. Matta, H. Paulsen, A. A. Grey, R. N. Shah and H. Schachter, *Carbohydr. Res.,* 1992, **236**, 281–299.
6. T. Ogawa, K. Katano, K. Sasajima and M. Matsui, *Tetrahedron,* 1981, **37,** 2779–2786.

Table S1. ^1H and ^{13}C Chemical Shift Assignments[a] for βGlcNAc-(1→2)-αManOCH$_3$ (**6**).

^1H nuclei							
H1	H2	H3	H4	H5	H6a	H6b	OCH$_3$
4.742	4.032	3.745	3.459	~3.54	~3.88	3.599	3.382
H1'	H2'	H3'	H4'	H5'	H6a'	H6b'	O=C–C<u>H</u>$_3$
4.527	3.677	3.526	3.434	~3.39	~3.89	~3.73	2.029
**^{13}C nuclei**							
C1	C2	C3	C4	C5	C6	OCH$_3$	
100.59	78.84	72.23	69.97	75.33	64.25	57.49	
C1'	C2'	C3'	C4'	C5'	C6'	C=O	O=C–<u>C</u>H$_3$
102.17	57.99	75.99	72.57	78.48	63.27	177.47	24.99

[a]In ppm; in ^2H_2O at 22 °C; ± 0001 ppm for ^1H; ± 0.01 for ^{13}C. Referenced externally to 2-dimethyl-2-silapentane-5-sulfonic acid (DSS) (0 ppm). Values with a ~ symbol were estimated from gCOSY or gHSQC 2D NMR spectra and have errors of ± 0.01 ppm.

Table S2. Other NMR Spin-Coupling Constants[a] in βGlcNAc-(1→2)-αManOCH$_3$ (**6**).

**Man residue**						
$^3J_{H1,H2}$	$^3J_{H2,H3}$	$^3J_{H3,H4}$	$^3J_{H4,H5}$	$^3J_{H5,H6a}$	$^3J_{H5,H6b}$	$^2J_{H6a,H6b}$
1.7	3.5	9.5	9.7	1.8	7.2	–11.7
**GlcNAc residue**						
$^3J_{H1',H2'}$	$^3J_{H2',H3'}$	$^3J_{H3',H4'}$	$^3J_{H4',H5'}$	$^3J_{H5',H6a'}$	$^3J_{H5',H6b'}$	$^2J_{H6a',H6b'}$
8.4	10.4	10.4	9.8	1.9	5.6	–12.4
**^{13}C-^{13}C spin-couplings (intra-residue)**						
$^1J_{C1,C2}$	$^1J_{C2,C3}$	$^3J_{C2,O\underline{C}H3}$	$^1J_{C1',H1'}$	$^1J_{C1',C2'}$	$^2J_{C1',C3'}$	$^3J_{C1',C6'}$
47.4	38.3	3.5	160.9	45.0	+4.5	4.0

[a]In Hz, ±0.1 Hz; ; in ^2H_2O at 22 °C.

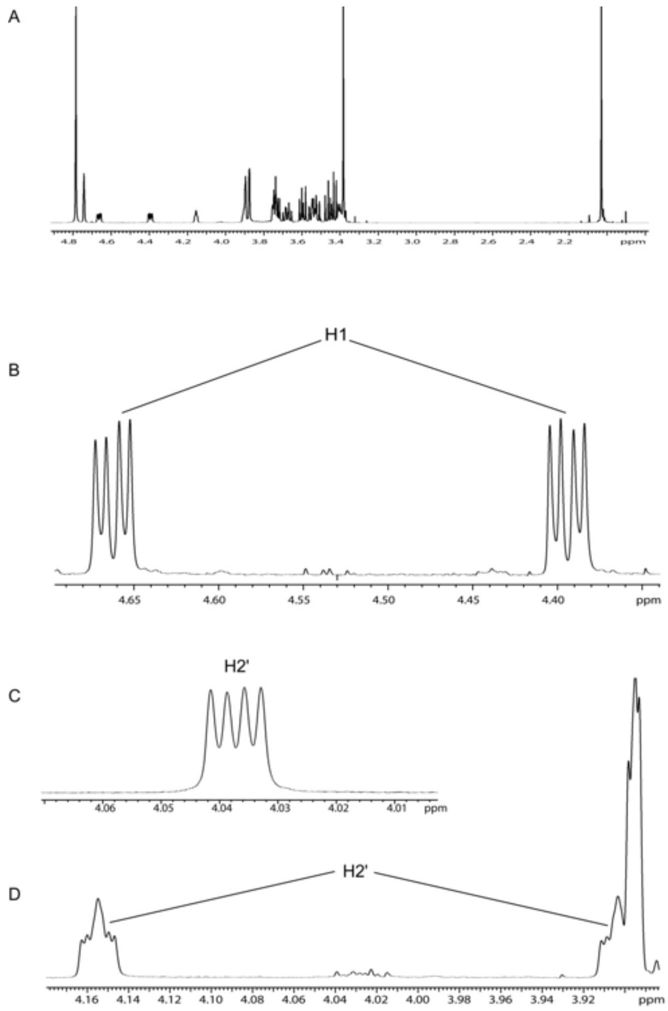

Figure S1. (A) Full ^{1}H NMR spectrum (600 MHz) of disaccharide $\mathbf{6}^{1',2}$ in $^{2}H_2O$. (B) Expanded 4.35–4.70 ppm region of the spectrum in (A) showing the H1' signal split by $^{1}J_{C1',H1'}$ (160.9 Hz), $^{3}J_{H1',H2'}$ (8.4 Hz), and $^{3}J_{C2,H1'}$ (3.9 Hz). (C) Partial ^{1}H NMR spectrum (600 MHz) of unlabeled **6** in $^{2}H_2O$ showing only the H2 signal split by $^{3}J_{H1,H2}$ (1.7 Hz) and $^{3}J_{H2,H3}$ (3.5 Hz). (D) Expanded 3.89–4.18 ppm region of the spectrum in (A) showing the H2 signal split by $^{1}J_{C2,H2}$ (~150 Hz), $^{3}J_{H1,H2}$ (1.7 Hz), $^{3}J_{H2,H3}$ (3.5 Hz) and $^{3}J_{C1',H2}$ (4.3 Hz).

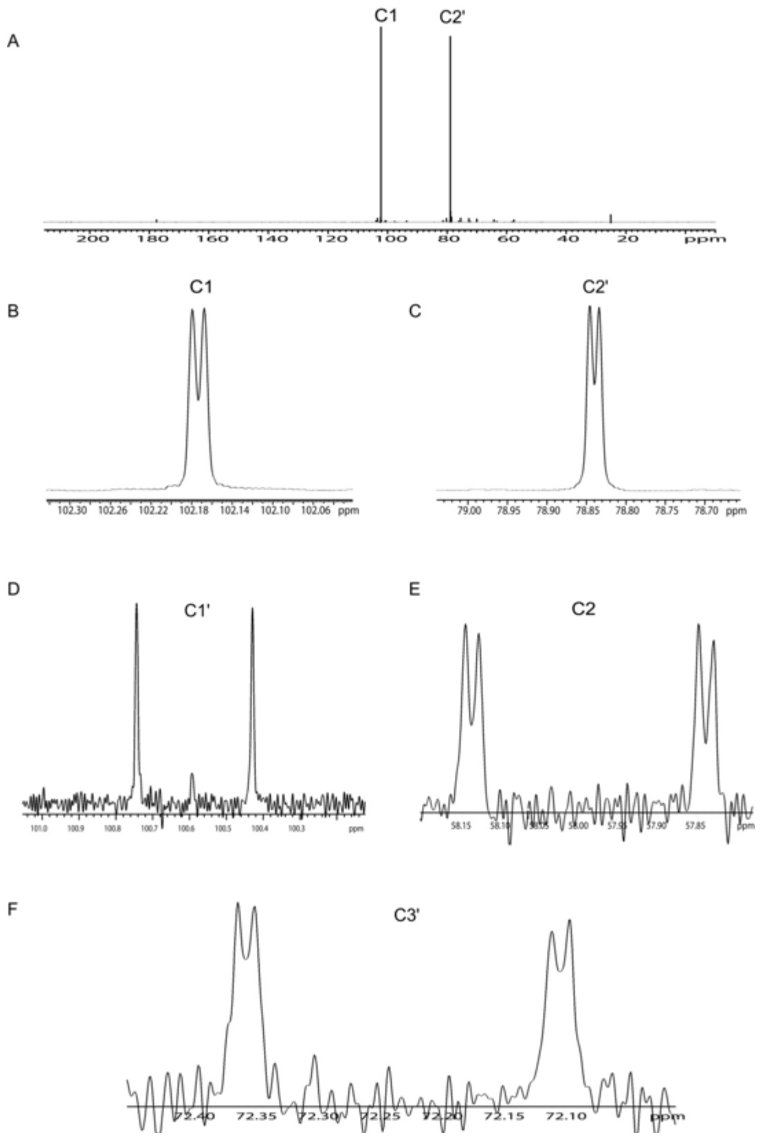

Figure S2. (A) Full $^{13}C\{^{1}H\}$ NMR spectrum (150 MHz) of disaccharide $\mathbf{6}^{1',2}$ in $^{2}H_2O$ showing intense C1' and C2 signals (^{13}C-labeled carbons) and very weak signals from the remaining natural abundance carbons. (B) and (C) Expansions of the C1' and C2 signals, respectively, in the spectrum in (A), showing them to be mutually spin-coupled ($^{2}J_{C1',C2}$ = –1.8 Hz). (D) The natural abundance C1 signal in the spectrum in (A) split by $^{1}J_{C1,C2}$ (47.4 Hz). Each signal of the doublet is also broadened by $^{3}J_{C1',C1}$ (~0.5 Hz). (E) The natural abundance C2' signal in the spectrum in (A) showing $^{1}J_{C1',C2'}$ (45.0 Hz) and $^{3}J_{C2',C2}$ (2.6 Hz). F. The natural abundance C3 signal in the spectrum in (A) showing $^{1}J_{C2,C3}$ (38.3 Hz) and $^{3}J_{C1',C3}$ (2.1 Hz).

Preparation of Disaccharide 7[1',4]

(Methyl 2-Deoxy-β-D-[1-^{13}C]-*arabino*-hexopyranosyl-(1→4)-β-D-[4-^{13}C]glucopyranoside)

(Scheme S1)

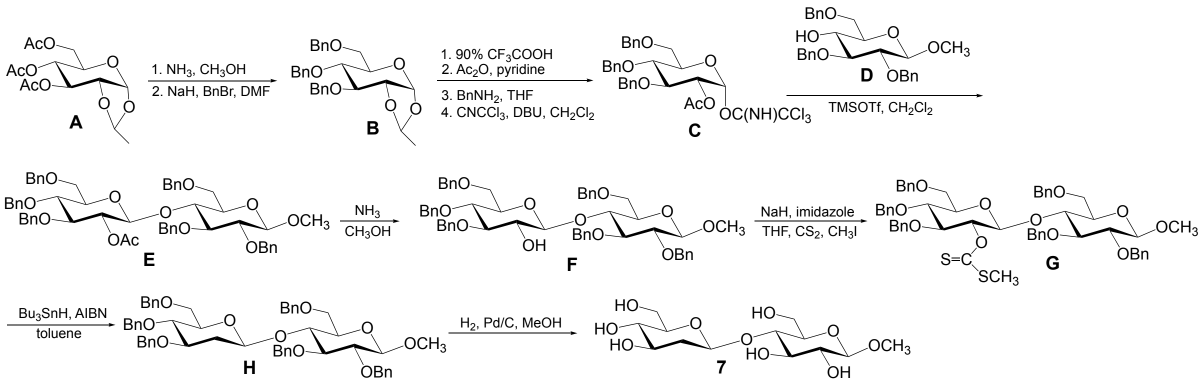

Scheme S1. Synthetic route to prepare deoxydisaccharide **7**.

All commercially-available reagents were used as purchased. Solutions were concentrated *in vacuo* using a rotary evaporator. Analytical thin-layer chromatography (TLC) was performed on precoated silica gel 60 F-254 aluminum plates. TLC visualization involved UV light and/or charring after spraying with 1% (w/v) $CeSO_4$–2.5% (*w/v*) $(NH_4)_6Mo_7O_{24}$ – 10% aq. H_2SO_4 reagent.[1]

High-resolution 1D ^{1}H and ^{13}C{^{1}H} NMR spectra were obtained using 5-mm NMR tubes and a Varian 600-MHz FT-NMR spectrometer equipped with a 5-mm ^{1}H-^{19}F/^{15}N-^{31}P AutoX dual broadband probe. NMR spectra of intermediates were collected in $CDCl_3$ at 22 °C. ^{1}H NMR spectra were typically collected with a ~6,000 Hz spectral window and a ~4.0 s recycle time. ^{13}C{^{1}H} NMR spectra were collected with ~30,000 Hz spectral windows and ~3.0 s recycle times. ^{1}H and ^{13}C chemical shifts were referenced internally to chloroform. NMR spectra of final products were collected in ^2H_2O at 22 °C and ^{1}H and ^{13}C chemical shifts were referenced externally to sodium 4,4-dimethyl-4-silapentane-1-sulfonate (DSS).

(1) *3,4,6-Tri-O-benzyl-1,2-O-S/R-ethylidene-α-D-glucopyranose (**B**)*. 3,4,6-Tri-*O*-acetyl-1,2-*O*-*S*/*R*-ethylidene-α-D-glucopyranose (**A**) was prepared using published methods.[2] Compound **A** (1.00 g, 3.00 mmol) was added to a saturated solution of NH_3 in MeOH (50 mL). After 16 h at rt, the reaction mixture was concentrated to dryness. The syrup was dissolved in DMF (30 mL) and NaH (60%, 0.60 g, 15.0 mmol) was added to the solution. After stirring at rt for 0.5 h, benzyl bromide (1.8 mL, 15.0 mmol) was added dropwise at 0 °C and the mixture was stirred at rt overnight. The mixture was diluted with CH_2Cl_2 (50 mL) and washed with distilled water. The organic phase was dried over anhydrous Na_2SO_4, concentrated *in vacuo* to dryness, and the residue purified by flash chromatography on silica gel (4:1 hexane/ethyl acetate, *v*/*v*) to afford **B** (1.24 g, 2.60 mmol, 87%). **B**: ^{1}H NMR (600 MHz, $CDCl_3$): δ 7.46–7.29 (m, 15H), 5.70 (d, *J* = 5.0 Hz, 1H), 5.16 (dd, *J* = 9.7, 4.8 Hz, 1H), 4.75 (d, *J* = 12.0 Hz, 1H), 4.69 (d, *J* = 12.2 Hz, 1H), 4.67 (d, *J* = 11.9 Hz, 1H), 4.66 (d, *J* = 11.6 Hz, 1H), 4.60 (d, *J* = 12.2 Hz, 1H), 4.47 (d, *J* = 11.6 Hz, 1H), 4.19 (m, 1H), 4.06 (m, 1H), 4.03 (t, *J* = 3.5 Hz, 1H), 3.85 (dd, *J* = 9.6, 3.6 Hz, 1H),

3.77 (m, 2H), 1.56 (d, *J* = 4.9 Hz, 3H). $^{13}C\{^1H\}$ NMR (150 MHz, $CDCl_3$): δ 138.2, 137.9, 137.8, 128.4, 128.3, 128.3, 128.0, 127.9, 127.8, 127.5, 100.5, 97.2, 78.0, 75.7, 75.1, 73.2, 72.5, 71.7, 69.8, 69.1, 19.9.

(2) *2-O-Acetyl-3,4,6-tri-O-benzyl-α-D-glucopyranosyl trichloroacetimidate (**C**).* Compound **B** (1.20 g, 2.52 mmol) was dissolved into a 90% CF_3COOH aqueous solution and stirred for 4 h at rt. The reaction mixture was concentrated *in vacuo* to dryness, and the residue was dissolved in pyridine (25 mL). After acetic anhydride (1.0 mL) was added, the reaction mixture was stirred overnight at rt. The mixture was concentrated, and the residue was dissolved in CH_2Cl_2 (50 mL) and washed with distilled water. The organic phase was dried over anhydrous Na_2SO_4 and concentrated *in vacuo* to dryness. THF (20 mL) and benzylamine (0.34 mL, 3.15 mmol) were added to the residue, and the reaction mixture was stirred overnight at rt. After purification on silica gel column (3:1 hexane/ethyl acetate, *v*/*v*), the product was converted to the corresponding trichloroacetimidate with trichloroacetonitrile and 1,8-diazobicyclo [5.4.0]-undec-7-ene (DBU) as described by Schmidt and coworkers[3], affording **C** (0.80 g, 1.25 mmol, 50%). **C**: ^1H NMR (600 MHz, $CDCl_3$): δ 8.58 (s, 1H), 7.36–7.18 (m, 15H), 6.54 (d, *J* = 3.6 Hz, 1H), 5.09 (dd, *J* = 10.0, 3.6 Hz, 1H), 4.88 (d, *J* = 11.5 Hz, 1H), 4.85 (d, *J* = 10.7 Hz, 1H), 4.79 (d, *J* = 11.6 Hz, 1H), 4.65 (d, *J* = 12.0 Hz, 1H), 4.59 (d, *J* = 10.7 Hz, 1H), 4.52 (d, *J* = 12.0 Hz, 1H), 4.11 (t, *J* = 9.6 Hz, 1H), 4.03 (m, 1H), 3.90 (t, *J* = 9.6 Hz, 1H), 3.83 (dd, *J* = 11.1, 3.4 Hz, 1H), 3.72 (dd, *J* = 11.1, 1.8 Hz, 1H), 1.95 (s, 3H). $^{13}C\{^1H\}$ NMR (150 MHz, $CDCl_3$): δ 170.2, 161.2, 138.5, 138.0, 138.0, 128.7, 128.6, 128.6, 128.4, 128.1, 128.0, 127.9, 94.2, 79.7, 77.2, 75.6, 75.6, 73.7, 73.6, 72.6, 68.1, 20.8.

(3) *Methyl 3,4,6-Tri-O-benzyl-β-D-glucopyranosyl-(1→4)-2,3,6-tri-O-benzyl-β-D-glucopyranoside (**F**).* Methyl 3,4,6-tri-O-benzyl-β-D-glucopyranose (**D**) was prepared using published methods.[4,5] To a mixture of compound **C** (0.50 g, 0.79 mmol), **D** (0.36 g, 0.78 mmol) and molecular sieves (4 Å, 2.0 g) was added anhydrous CH_2Cl_2 (20 mL). The solution was cooled to 0 ^{o}C and was treated with small amount of TMSOTf (0.02 mL) under a N_2 atmosphere overnight. The reaction mixture was quenched with the addition of triethylamine (few drops) and the molecular sieves were removed by filtration. The filtrate was concentrated and purified by flash chromatography on silica gel (hexanes/ethyl acetate, 3:1 *v*/*v*) to afford disaccharide **E** containing some unreacted **D**. The syrup of **E** was added to a saturated solution of NH_3 in MeOH (20 mL). After 4 days at rt, the reaction mixture was concentrated and the residue was purified by flash chromatography on silica gel (hexanes/ethyl acetate, 3:1 *v*/*v*) to afford compound **F** (0.48 g, 0.55 mmol, 70%). **F**: ^1H NMR (600 MHz, $CDCl_3$): δ 7.43-7.17 (m, 30H), 5.05 (d, *J* = 11.6 Hz, 1H), 4.94 (d, *J* = 11.1 Hz, 1H), 4.91 (d, *J* = 11.5 Hz, 1H), 4.83 (m, 2H), 4.76 (d, *J* = 12.0 Hz, 1H), 4.70-4.65 (m, 3H),4.53 (d, *J* = 10.9 Hz, 1H), 4.47 (d, *J* = 12.1 Hz, 1H), 4.42 (d, *J* = 12.1 Hz, 1H), 4.35 (d, *J* = 7.8 Hz, 1H), 4.07 (dd, *J* =9.6, 9.1 Hz, 1H), 4.03 (dd, *J* =11.6, 3.7 Hz, 1H), 3.84 (dd, *J* =11.6, 2.2 Hz, 1H), 3.72 (t, *J* = 9.1 Hz, 1H), 3.66-3.63 (m, 2H), 3.61 (s, 3H), 3.54-3.45 (m, 6H), 3.25 (m, 1H). $^{13}C\{^1H\}$ NMR (150 MHz, $CDCl_3$): δ 139.3, 139.0, 138.5, 138.4, 138.3, 137.6, 128.6, 128.5, 128.4, 128.4, 128.3, 128.3, 128.2, 128.1, 128.1, 127.9, 127.8, 127.6, 127.2, 127.0, 105.0, 103.5, 84.6, 83.7, 82.4, 77.3, 77.2, 76.0, 75.3, 75.2, 75.1, 74.9, 74.4, 73.9, 73.4, 68.9, 68.6, 57.2.

(4) *Methyl 2-O-(Methylthio)thiocarbonyl-3,4,6-tri-O-benzyl-β-D-glucopyranosyl-(1→4)-2,3,6-tri-O-benzyl-β-D-glucopyranoside (**G**).* The syrup of **F** (0.48 g, 0.55 mmol) was dissolved in THF (10 mL), imidazole (3 mg) was added, and then NaH (55 mg, 2.20 mmol) was added. The reaction mixture was stirred for 0.5 h at rt under a N_2 atmosphere. Carbon disulfide (0.26 mL, 4.4 mmol) was added, and the mixture was stirred for 1.5 h. Methyl iodide (0.14 mL, 2.2 mmol) was then added, and the mixture was stirred for an additional 1 h. The reaction mixture was diluted with ethyl acetate, washed with 1 *M* aqueous HCl, aqueous saturated $NaHCO_3$, and brine. The organic layer was dried over anhydrous Na_2SO_4. After evaporation of the solvent, the residue was purified by flash chromatography on silica gel (hexanes/ethyl acetate, 3:1 *v*:*v*) to afford compound **G** (0.41 g, 0.43 mmol, 78%).[6,7] **G**: ^{1}H NMR (600 MHz, $CDCl_3$): δ 7.55-7.32 (m, 30H), 6.15 (dd, *J* = 9.3, 8.1 Hz, 1H), 5.21 (d, *J* = 11.6 Hz, 1H), 5.01 (d, *J* = 11.1 Hz, 1H), 4.96-4.80 (m, 7H), 4.69 (d, *J* = 11.0 Hz, 1H), 4.66 (d, *J* = 12.2 Hz, 1H), 4.57-4.53 (m, 2H), 4.42 (d, *J* = 7.8 Hz, 1H), 4.13 (dd, *J* = 9.4, 9.1 Hz, 1H), 4.00 (dd, *J* =11.1, 3.9 Hz, 1H), 3.93-3.90 (m, 2H), 3.81-3.69 (m, 4H), 3.70 (s, 3H), 3.56 (dd, *J* =9.0, 7.8 Hz, 1H), 3.52-3.50 (m, 1H), 3.46-3.44 (m, 1H), 2.69 (s, 3H). ^{13}C{^{1}H} NMR (150 MHz, $CDCl_3$): δ 215.2, 139.3, 138.6, 138.3, 138.2, 138.2, 137.9, 128.6, 128.4, 128.3, 128.2, 128.1, 128.0, 127.8, 127.7, 127.6, 127.5, 127.1, 104.6, 100.2, 83.0, 82.7, 82.0, 81.8, 77.8, 76.9, 75.2, 75.2, 74.9, 74.9, 74.7, 74.5, 73.5, 73.3, 68.5, 68.2, 57.0, 19.6.

(5) *Methyl 2-Deoxy-3,4,6-tri-O-benzyl-β-D-arabino-hexopyranosyl-(1→4)-2,3,6-tri-O-benzyl-β-D-glucopyranoside (**H**).* A solution of tri-*n*-butyltin hydride (0.70 g) and AIBN (20 mg) in toluene (8.0 mL) was added to compound **G** (0.33 g, 0.34 mmol) in toluene (10 mL) dropwise under a N_2 atmosphere and at 80 ^oC. The reaction mixture was kept stirring for 3 h. After the solvent was removed with a rotary evaporator, acetonitrile (10 mL) and hexane (10 mL) were added to the residue, and the two-phase solution was stirred vigorously for 15 min. The lower acetonitrile layer was then separated, and the hexane phase was washed with acetonitrile (5.0 mL). Extraction of the combined acetonitrile solutions was repeated twice. The combined acetonitrile phase was concentrated and the residue was purified by flash chromatography on silica gel (hexanes/ethyl acetate, 3:1 *v*/*v*) to give product **H** (0.18 g, 0.20 mmol, 59%).[8] **H**: ^{1}H NMR (600 MHz, $CDCl_3$): δ 7.38-7.21 (m, 30H), 5.05 (d, *J* = 11.2 Hz, 1H), 4.91-4.87(m, H), 4.84 (d, *J* = 11.3 Hz, 1H), 4.72-4.66 (m, 3,H), 4.58-4.55 (m, 3H), 4.50 (d, *J* = 11.5 Hz, 1H), 4.49 (d, *J* = 12.1 Hz, 1H), 4.44 (d, *J* = 12.1 Hz, 1H), 4.33 (d, *J* = 7.8 Hz, 1H), 3.92 (t, *J* = 9.3 Hz, 1H), 3.79 (dd, *J* =11.0, 2.2 Hz, 1H), 3.79 (dd, *J* =10.9, 4.1 Hz, 1H), 3.66 (t, *J* = 9.0 Hz, 1H), 3.60 (m, 2H), 3.60 (s, 3H), 3.53-3.47 (m, 2H), 3.43 (dd, *J* =9.1, 7.8 Hz, 1H), 3.30-3.27 (m, 1H), 2.28 (ddd, *J* =12.5, 5.0, 1.9 Hz, 1H), 1.57 (ddd, *J* =12.5, 11.3, 9.8 Hz, 1H). ^{13}C{^{1}H} NMR (150 MHz, $CDCl_3$): δ 139.3, 138.7, 138.6, 138.6, 138.6, 138.3, 128.6, 128.5, 128.5, 128.4, 128.3, 128.3, 128.2, 127.9, 127.8, 127.6, 127.4, 104.9, 100.1, 83.3, 82.2, 79.5, 78.1, 76.4, 75.5, 75.3, 75.1, 75.0, 74.8, 73.6, 73.5, 71.5, 69.3, 69.0, 57.3, 29.9.

(6) *Methyl 2-deoxy-β-D-arabino-hexopyranosyl-(1→4)-β-D-glucopyranoside (**7**).* Compound **H** (180 mg, 0.20 mmol) was dissolved in methanol (10.0 mL) and treated with Pd/C (10%, 50 mg) and H_2 overnight to remove *O*-benzyl groups. The Pd/C catalyst was removed by filtration and the reaction solution was dried at 30 ^oC *in vacuo*. The residue was dissolved in ~1 mL of distilled water and the solution was applied to a column (2.5 x 100 cm) containing Biogel

P2 gel filtration resin. The column was eluted with distilled, decarbonated water at ~1.5 mL/min, and fractions (5 mL) were collected and assayed by TLC. Fractions containing disaccharide **7** were pooled and concentrated at 30 ^{o}C in vacuo (54 mg, 0.16 mmol, 80%). ^{1}H and ^{13}C chemical shifts, and ^{1}H-^{1}H spin-coupling constants in **7** are shown in Tables S1 and S2.

(7) *Preparation of ^{13}C-Labeled Disaccharide* $\mathbf{7}^{1',4}$. Doubly ^{13}C-labeled disaccharide $\mathbf{7}^{1',4}$ was prepared using the synthetic route shown in Scheme S1 but substituting [1-^{13}C]**C** and [4-^{13}C]**D** in the protocol. [1-^{13}C]**C** and [4-^{13}C]**D** were prepared from D-[1-^{13}C]glucose and D-[4-^{13}C]glucose, respectively, which were obtained from Omicron Biochemicals, Inc. (South Bend, IN). ^{13}C-^{13}C Spin-coupling constants in $\mathbf{7}^{1',4}$ are shown in Table S3.

References

1. F. D.Tropper, F. O. Andersson, C. Grant-Maitre and R. Roy, *Carbohydr. Res.,* 1992, **229,** 149–154.
2. W. Zhang, Q. Pan and A. S. Serianni, *J. Label. Compd. Radiopharm.,* 2016, **59***,* 673–679.
3. R. R. Schmidt and J. Michel, *J. Carbohydr. Chem.,* 1985, **4**, 141–169.
4. M. Sakagami and H. Hamana, *Tetrahedron Lett*., 2000, **41**, 5547–5551.
5. W. Zhang, T. Turney, R. Meredith, Q. Pan, L. Sernau, X. Wang, X. Hu, R. J. Woods, I. Carmichael and A. S. Serianni, *J. Phys. Chem. B,* 2017, **121**, 3042–3058.
6. A. K. Sanyal, and C. B. Purves, *Can. J. Chem*., 1956, **34**, 426–435.
7. S. V. Chetyrkin, W. Zhang, B. G. Hudson, A. S. Serianni and P. A. Voziyan, *Biochemistry,* 2008, **47**, 997–1006.
8. D. H. R. Barton and S. W. McCombie, *J. Chem. Soc., Perkin Trans. 1,* 1975, **16**, 1574–1585.

^{1}H (top) and ^{13}C{^{1}H} (bottom) NMR Spectra of Compound **B**

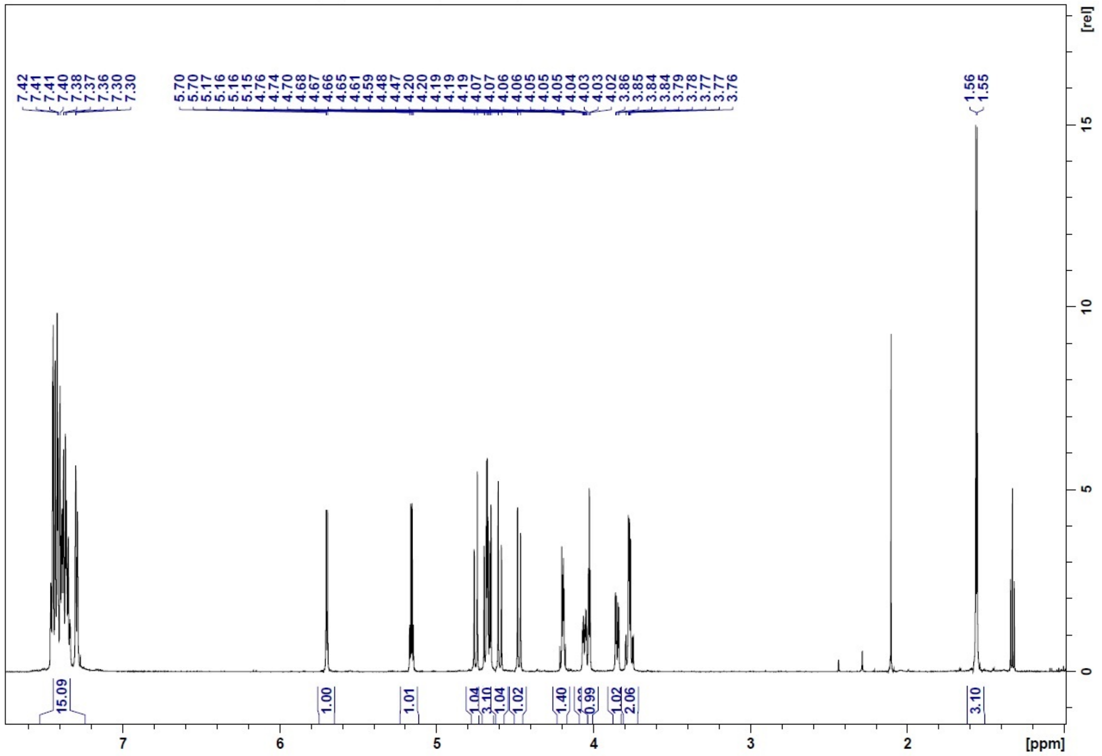

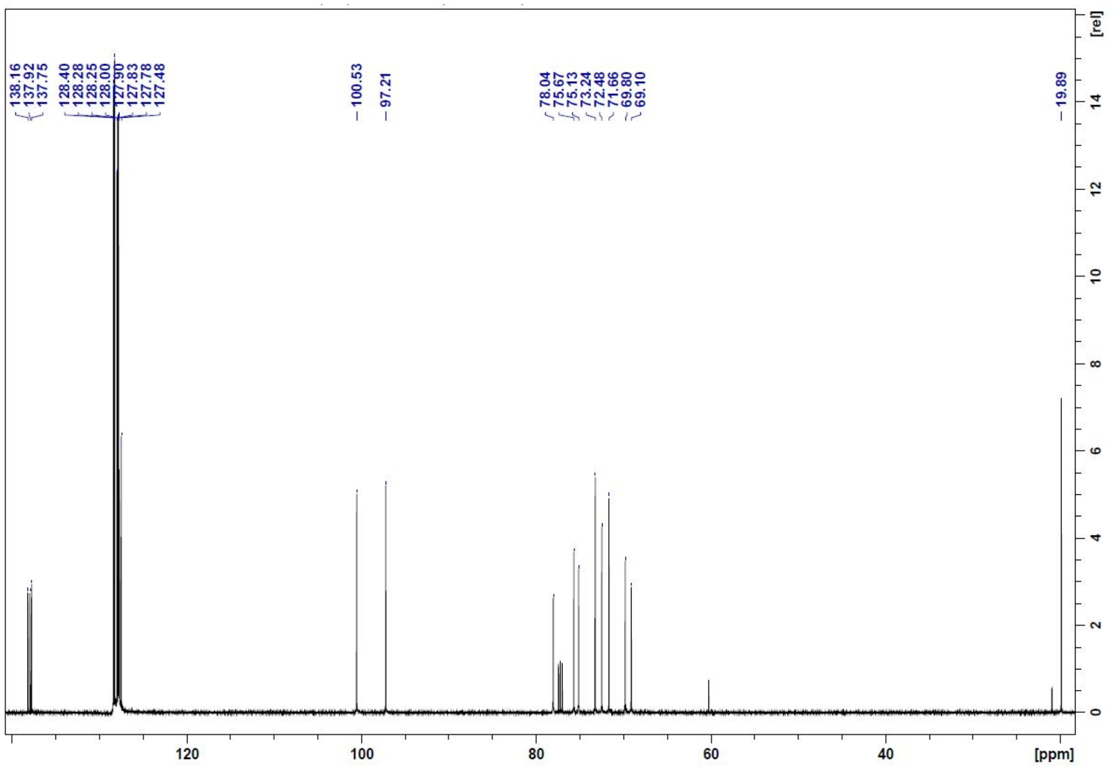

^{1}H (top) and ^{13}C{^{1}H} (bottom) NMR Spectra of Compound **C**

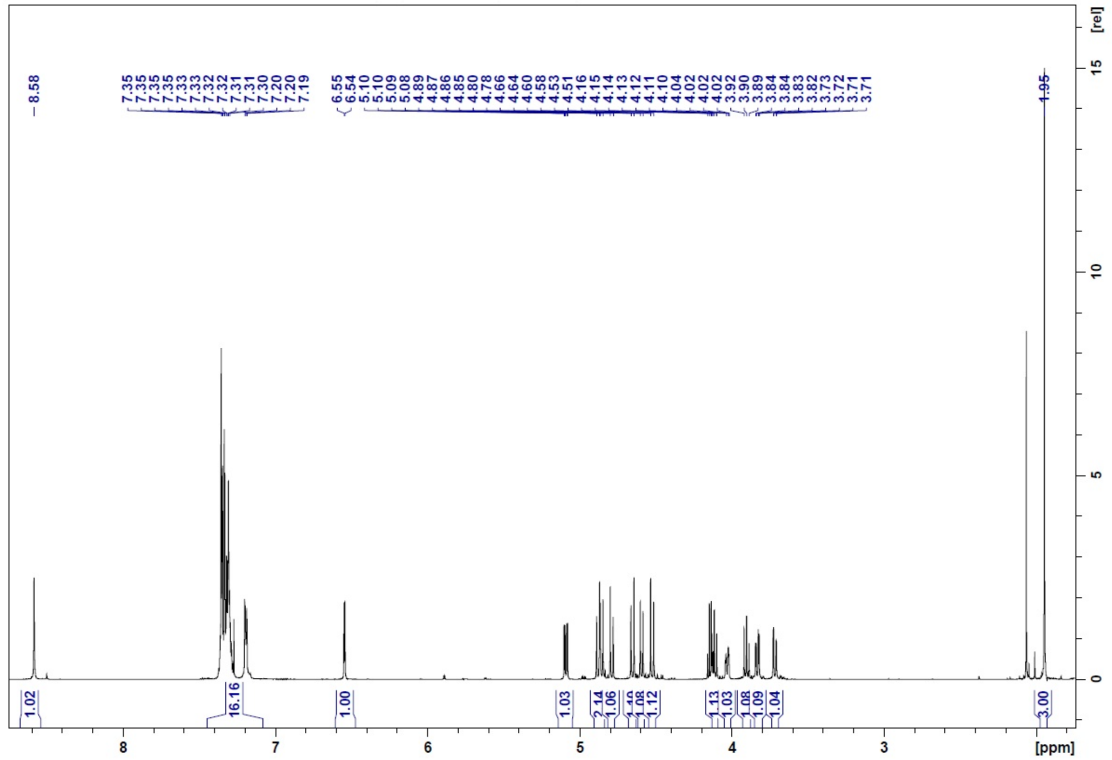

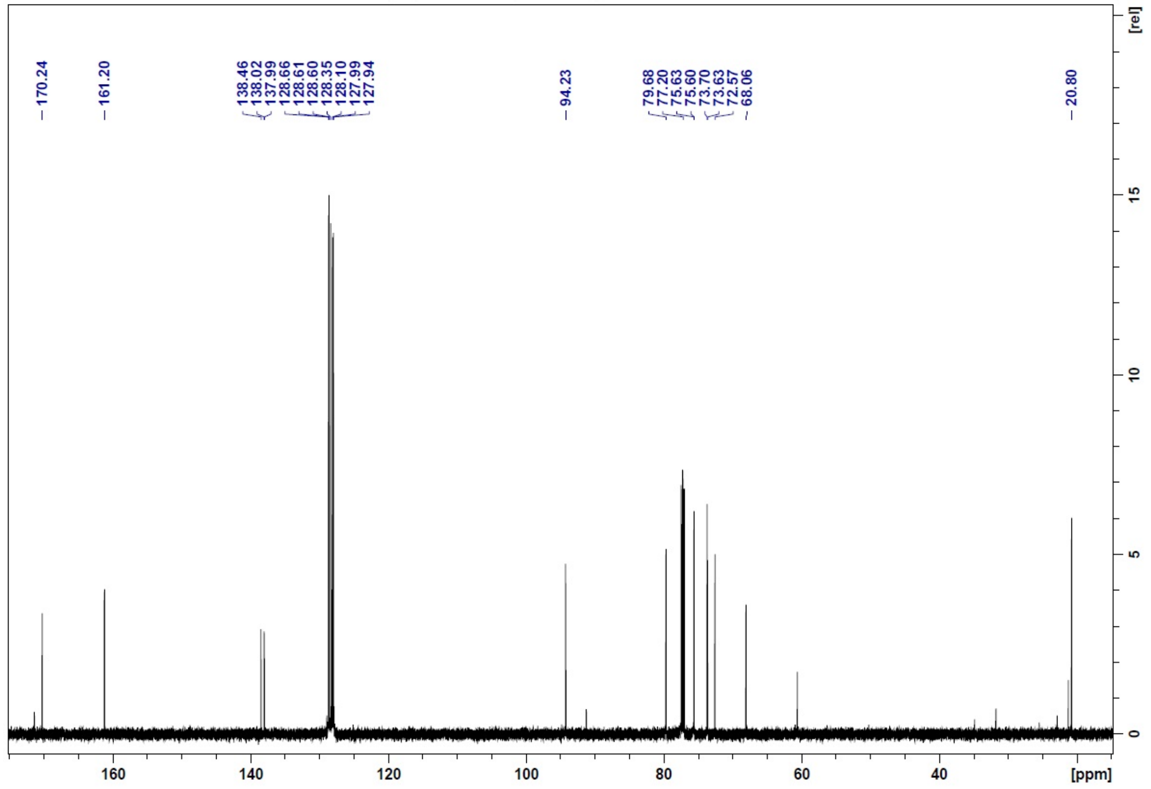

^{1}H (top) and ^{13}C{^{1}H} (bottom) NMR Spectra of Compound **F**

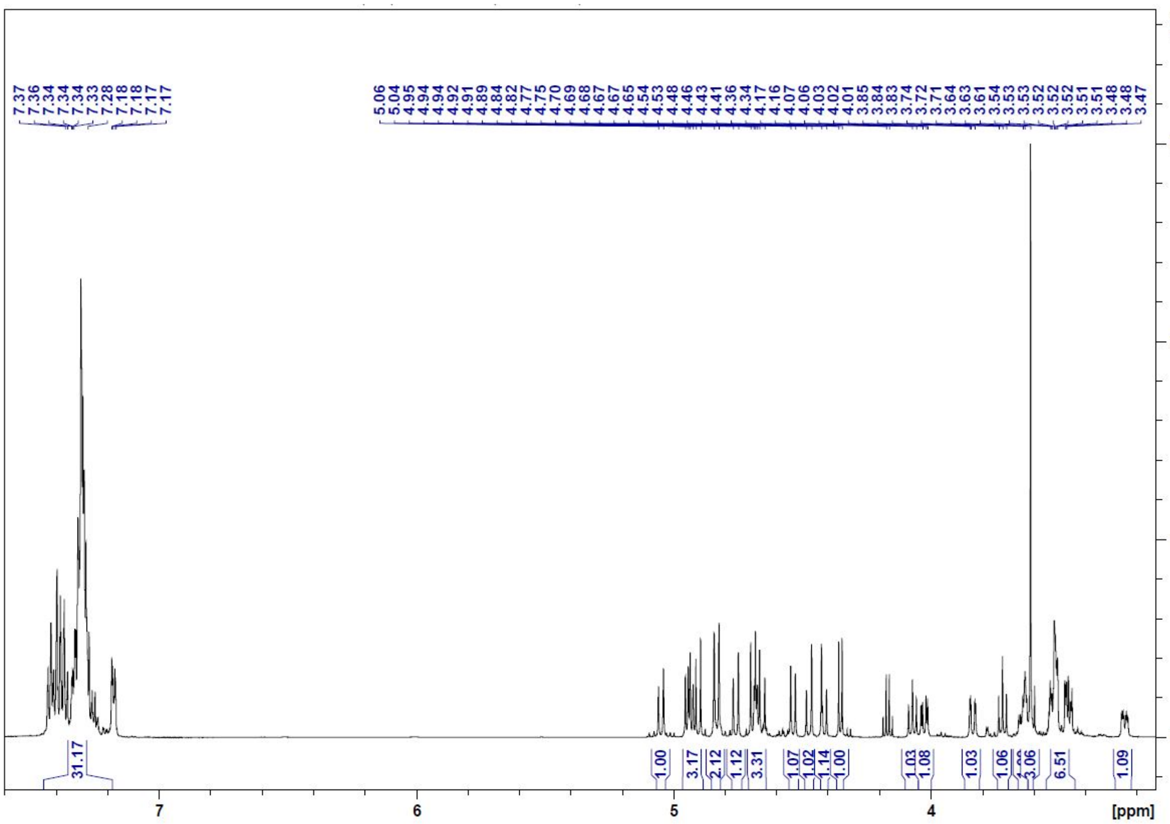

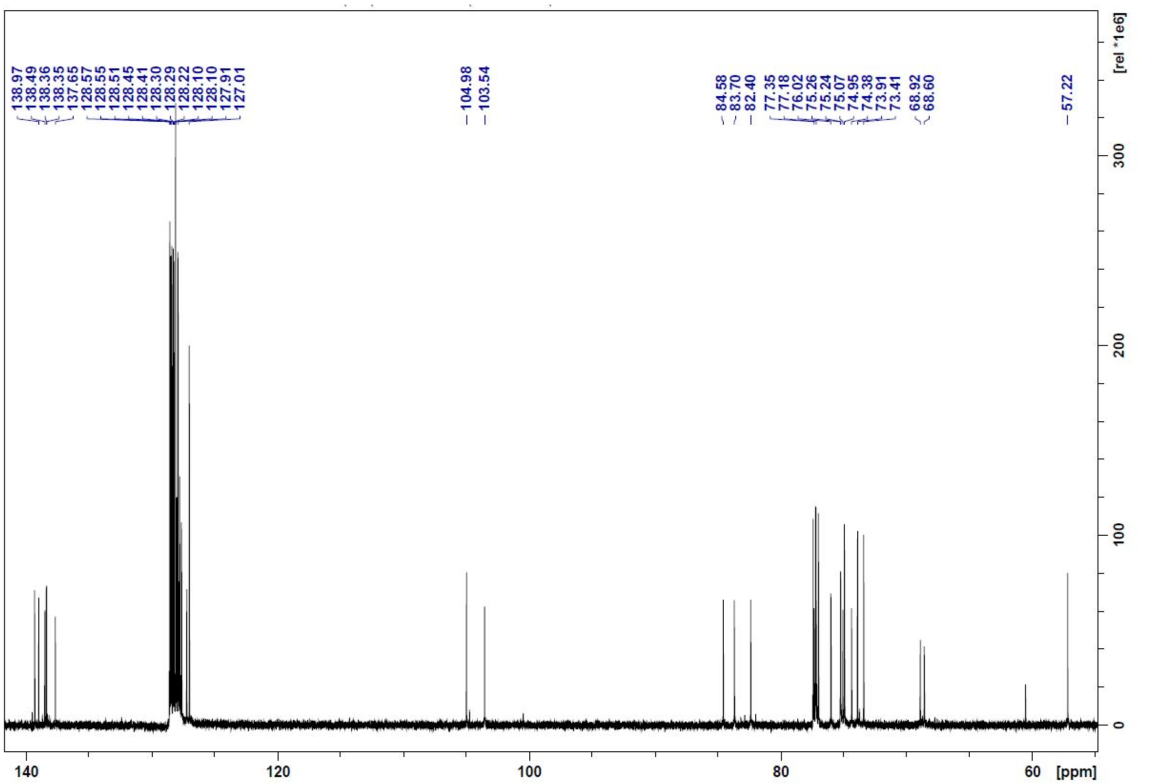

^{1}H (top) and $^{13}C\{^{1}H\}$ (bottom) NMR Spectra of Compound **G**

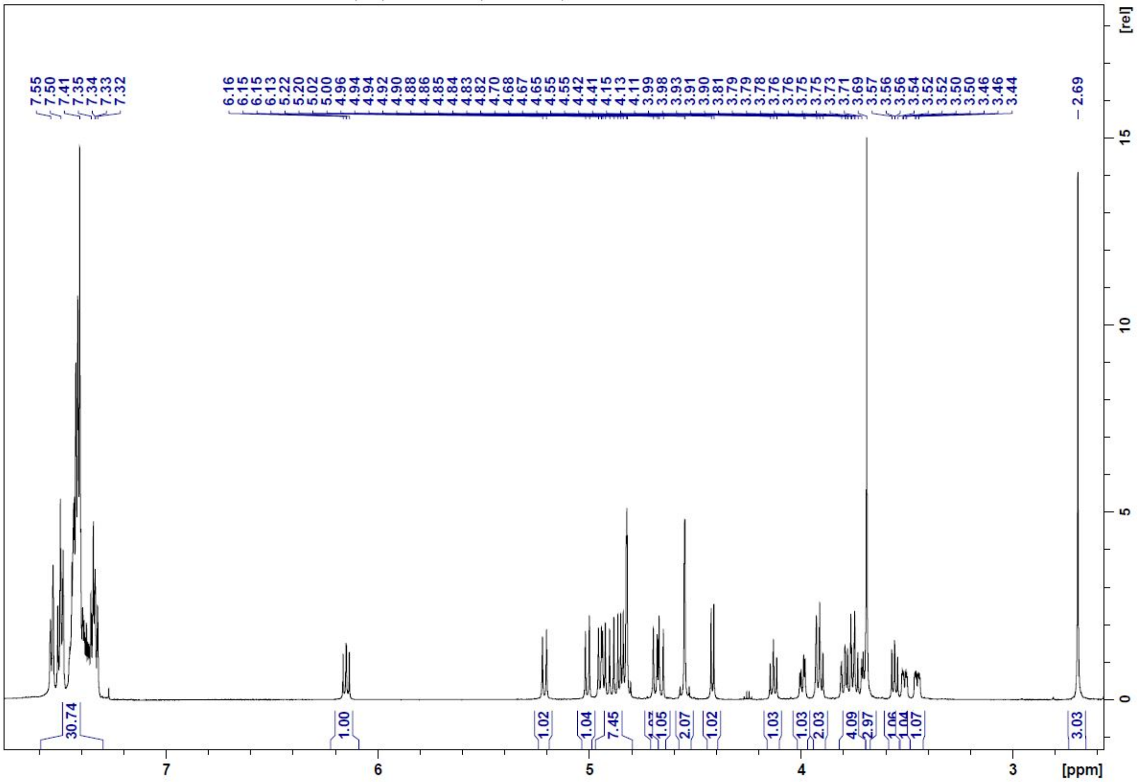

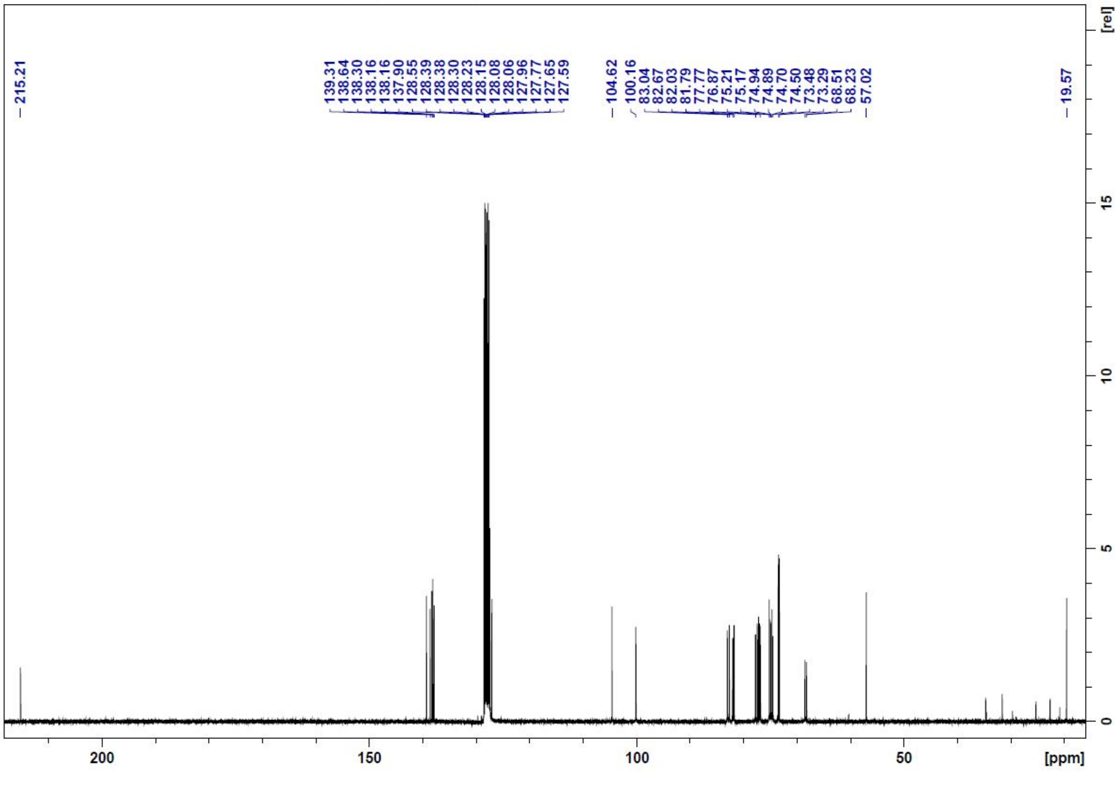

^{1}H (top) and $^{13}C\{^{1}H\}$ (bottom) NMR Spectra of Compound **H**

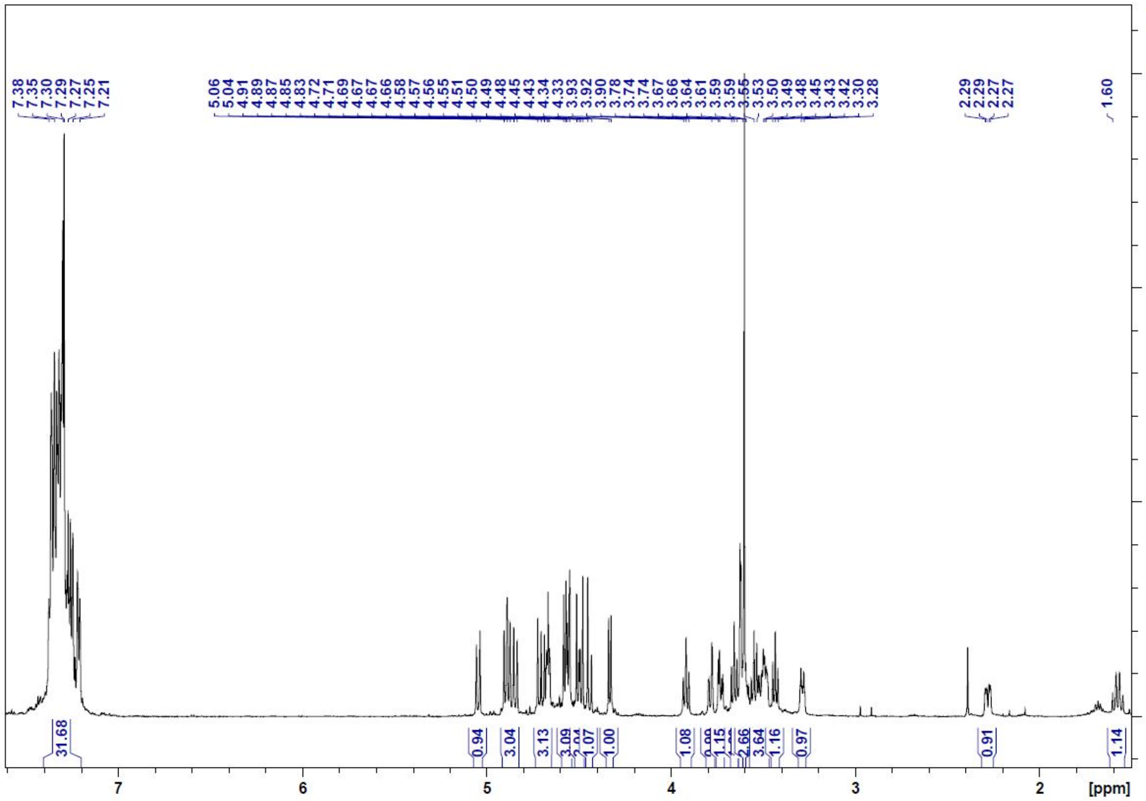

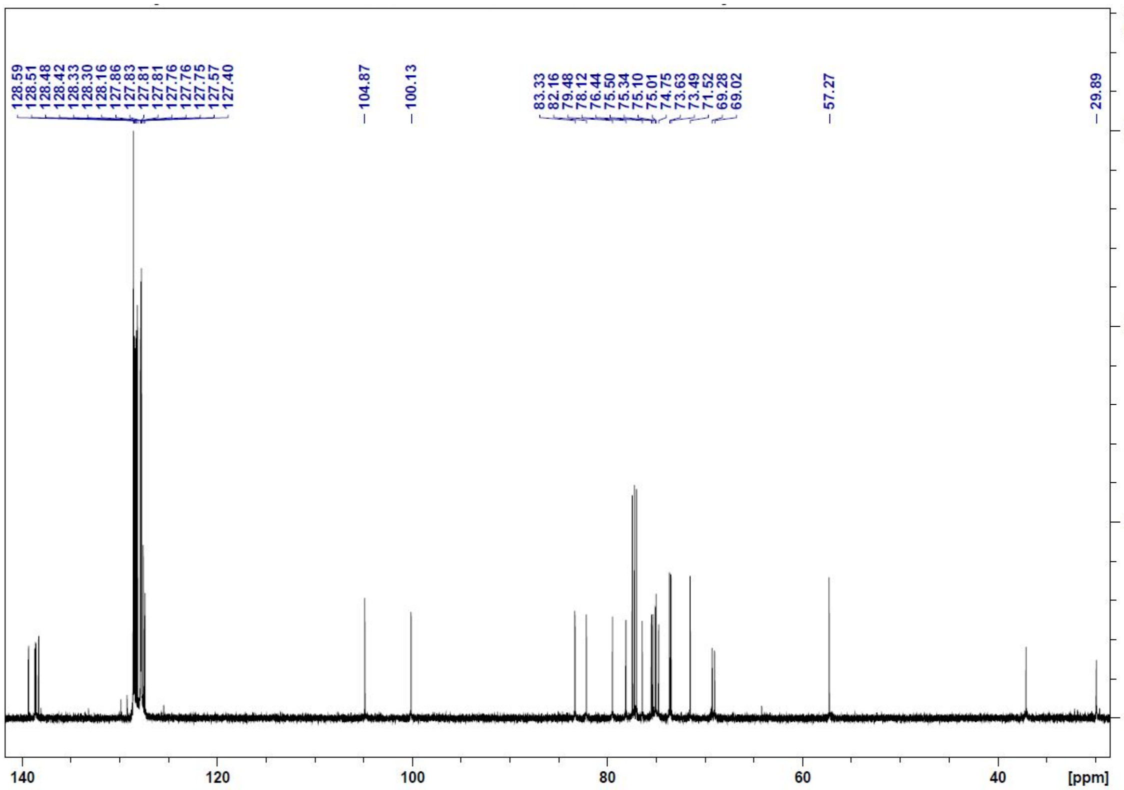

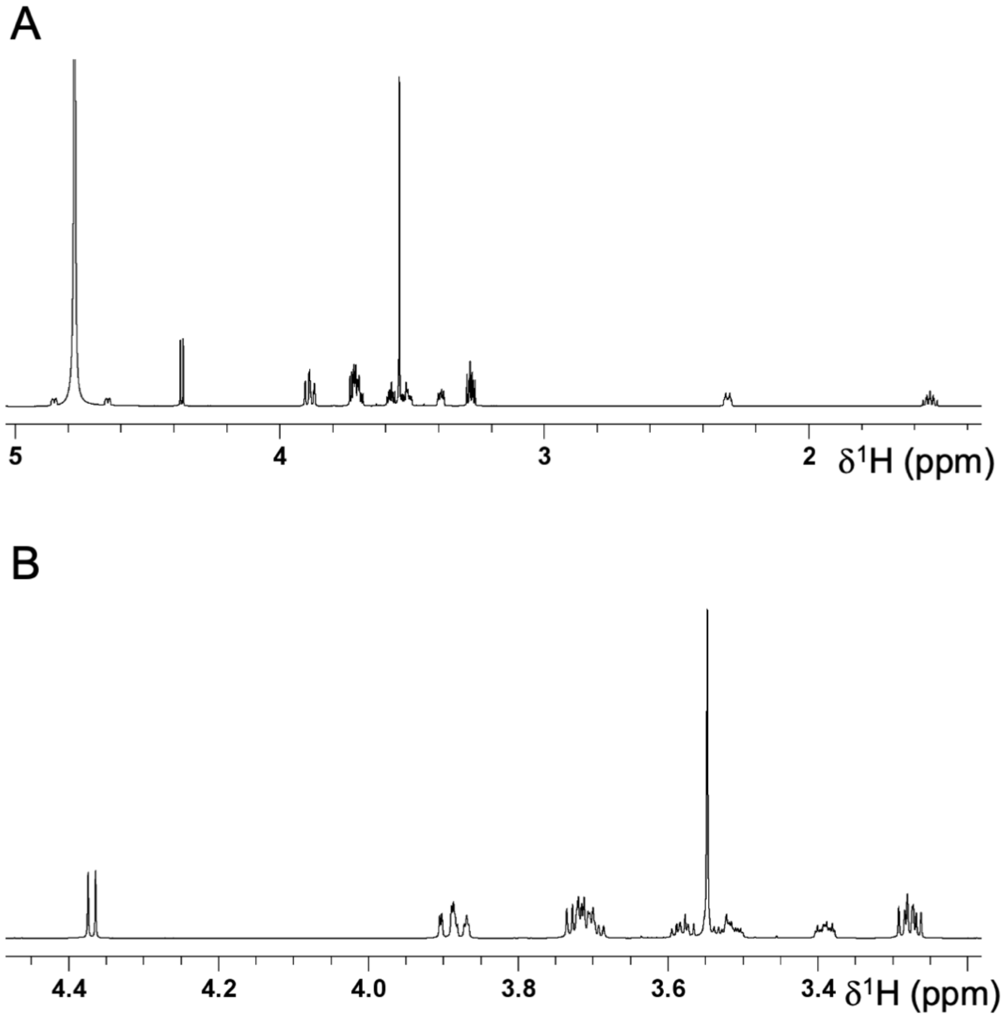

Figure S1. (A) ^{1}H NMR spectrum (800 MHz) of disaccharide **7**$^{1',4}$ in ^2H_2O and ~25 ^oC showing all signals. The intense signal at ~4.8 ppm is the residual HOD signal. (B) An expansion of the 3.5–4.4 ppm region of the spectrum shown in (A).

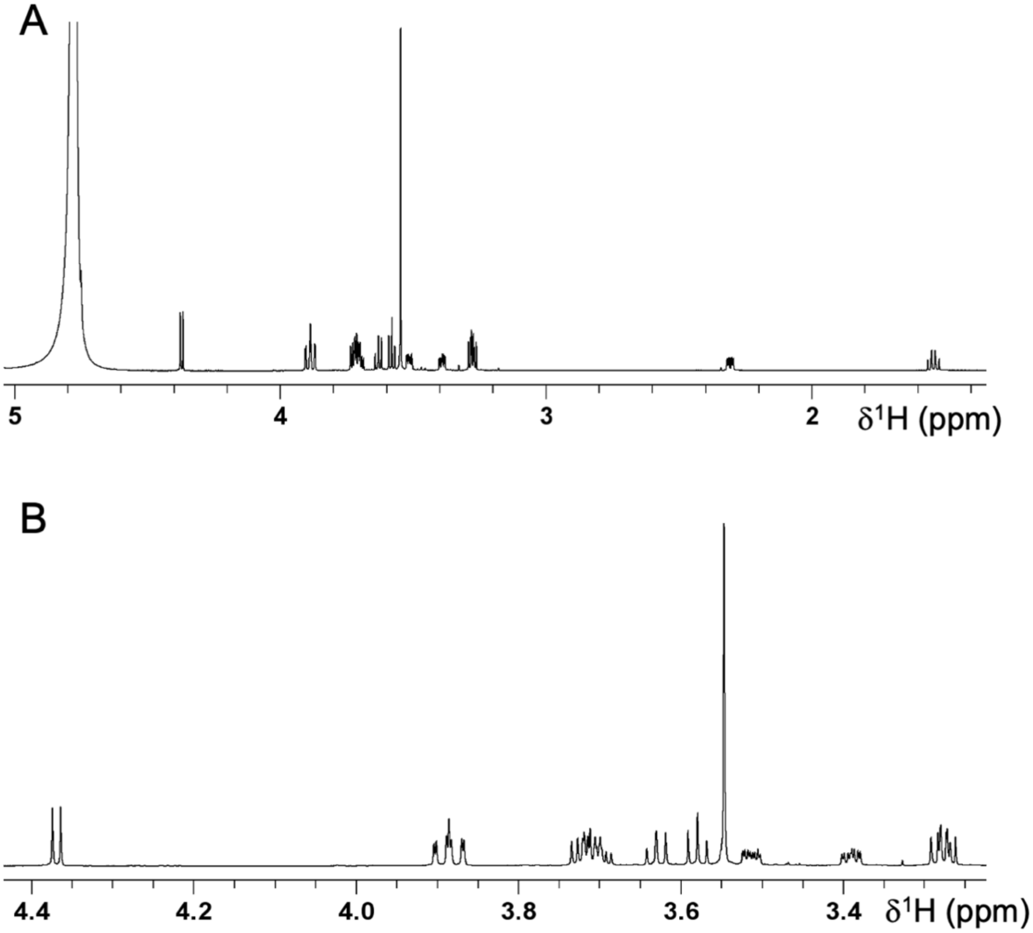

Figure S2. (A) ^{1}H NMR spectrum (800 MHz) of disaccharide **7** (unlabeled) in $^{2}H_2O$ and ~25 ^{o}C showing all signals. The off-scale signal at ~4.8 ppm is the residual HOD signal. (B) An expansion of the 3.5–4.4 ppm region of the spectrum shown in (A).

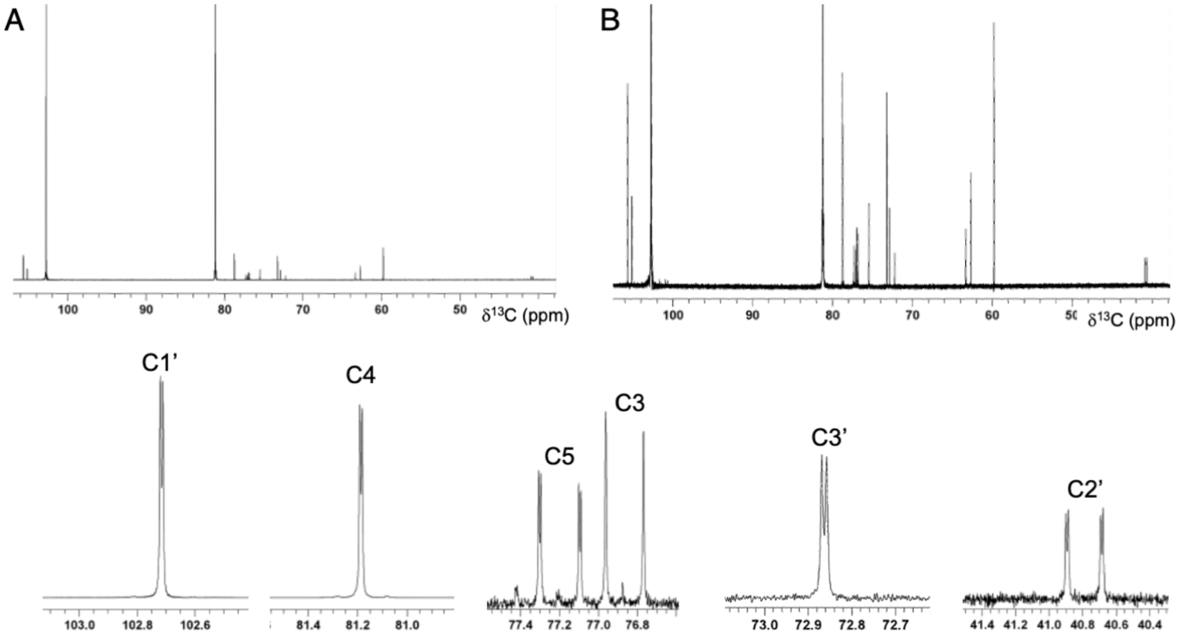

Figure S3. (A) $^{13}C\{^{1}H\}$ NMR spectrum (200 MHz) of $\mathbf{7}^{1',4}$ in $^{2}H_2O$ and ~25 ^{o}C showing all signals. The intense signals at ~103 ppm and ~81 ppm arise from the enriched C1' and C4 carbons, respectively. (B) An expansion of the 40–106 ppm region of the spectrum in A, showing all carbon signals except that from C2'. The upfield signal at ~105 ppm is a ^{13}C-labeled impurity. Insets on the bottom row show the splitting of the natural abundance C1', C4, C5, C3, C3' and C2' carbons in $\mathbf{7}^{1',4}$ caused by spin-coupling to the ^{13}C-enriched C1' and/or C4 carbons.

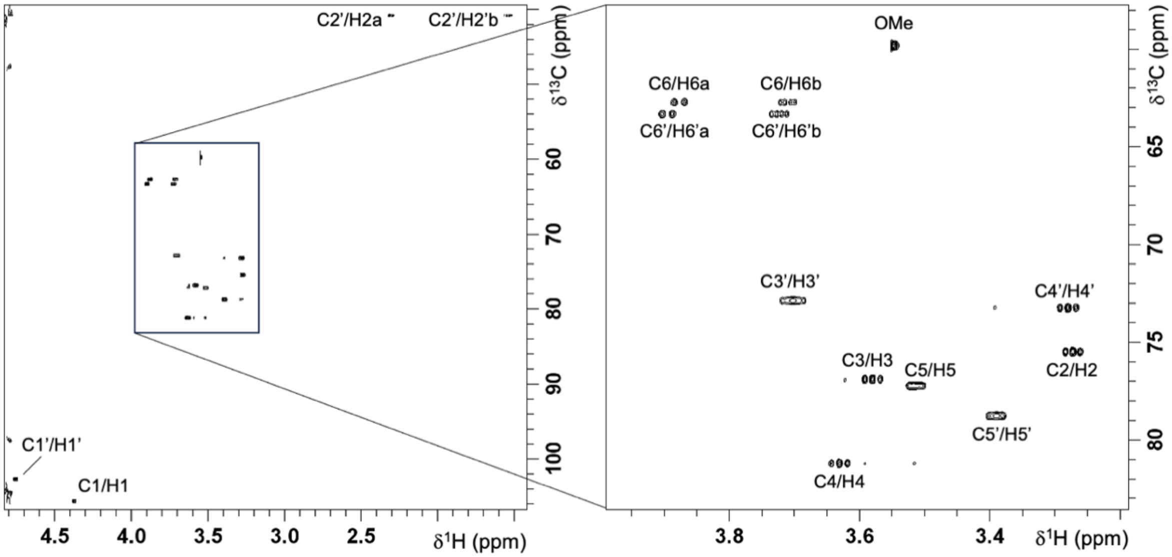

Figure S4. 2D ^{1}H-^{13}C HSQC spectrum (800 MHz) of disaccharide **7** (unlabeled) in $^{2}H_2O$ and ~25 ^{o}C showing C/H correlations that assisted in ^{1}H and ^{13}C chemical shift assignments (Table S1). The full 2D spectrum on the left shows the assignments of the C1/H1, C1'/H1', C2'/H2'a and C2'/H2'b cross peaks, and the expanded area of this spectrum on the right shows the assignments of the remaining C/H cross peaks.

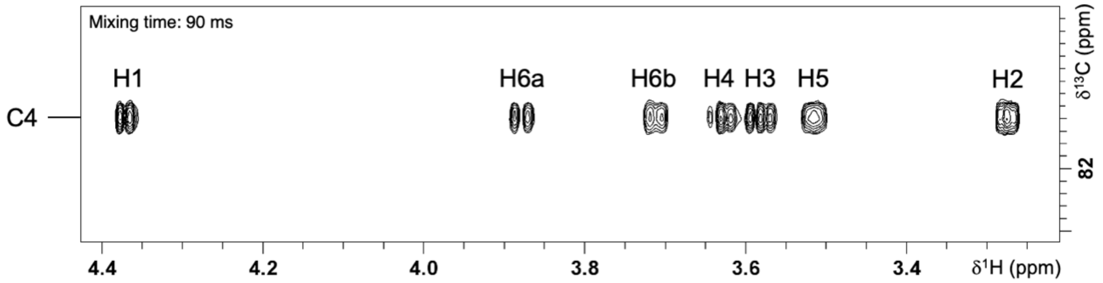

Figure S5. Slice of the 2D ^{1}H-^{13}C HSQC-TOCSY spectrum (800 Hz) of **7** in $^{2}H_2O$ and ~25 ^{o}C showing ^{1}H connectivities to C4 of the β-D-glucopyranosyl ring.

Table S1. ^{1}H and ^{13}C Chemical Shifts[a] for Disaccharide **7**.

residue	atom number	δ_H (ppm)	δ_C (ppm)
2dβGlc	1	4.754	102.74
	2a	2.305	40.82
	2b	1.540	
	3	3.701	72.88
	4	3.280	73.22
	5	3.389	78.77
	6a	3.895	63.34
	6b	3.724	
βGlc	1	4.370	105.69
	2	3.273	75.47
	3	3.579	76.88
	4	3.630	81.19
	6a	3.876	62.74
	6b	3.710	
	OMe	3.547	59.80

[a]In ppm, ±0.001 for ^{1}H, ±0.01 for ^{13}C, in ^2H_2O at ~25 ^{o}C.

Table S2. ${}^{1}H$-${}^{1}H$ Spin-Coupling Constants[a] in Disaccharide **7**.

<table>
<tr><th rowspan="2">coupled hydrogens</th><th colspan="2">residue</th></tr>
<tr><th>2dβGlc</th><th>βGlc</th></tr>
<tr><td>H1–H2a</td><td>1.9</td><td rowspan="2">8.0</td></tr>
<tr><td>H1–H2b</td><td>9.8</td></tr>
<tr><td>H2a–H3</td><td>5.1</td><td rowspan="2">9.2</td></tr>
<tr><td>H2b–H3</td><td>nm</td></tr>
<tr><td>H3–H4</td><td>nm</td><td>9.3</td></tr>
<tr><td>H4–H5</td><td>8.4</td><td>9.8</td></tr>
<tr><td>H5–H6a</td><td>–2.4</td><td>–2.2</td></tr>
<tr><td>H5–H6b</td><td>–6.0</td><td>–5.1</td></tr>
<tr><td>H2a–H2b</td><td>–12.5</td><td>–</td></tr>
<tr><td>H6a–H6b</td><td>–12.4</td><td>–12.4</td></tr>
</table>

[a]In Hz, in ${}^{2}H_2O$ at ~25 °C. H2b is more shielded than H2a; H6b is more shielded than H6a (see Table S1). Geminal couplings were assumed to have negative signs; *nm* denotes values that could not be measured.

Table S3. ^{13}C-^{13}C Spin-Coupling Constants[a] in Disaccharide **7**$^{1',4}$.

coupled carbons	residue	
	2dβGlc	βGlc
intra-residue ^{13}C-^{13}C couplings		
C1–C2	41.6	
C3–C4		39.1
C4–C5		41.7
C1–C3	±2.2[b]	
C1–C5	~0	
C2–C4		+2.5
C4–C6		~0
C1–C4	~0	~0
C1–C6	4.1	
inter-residue ^{13}C-^{13}C couplings[c]		
C1'–C4	–1.8	
C2'–C4	3.0	
C1'–C5	2.2	
C1'–C3	~0	

[a]In Hz, in $^{2}H_2O$ at ~25 °C. [b]Coupling sign unknown. [c]For inter-residue J_{CC} values, the primed atoms reside in the 2dβGlc residue.

Representative Cartesian Coordinates for DFT Structures 4^{c}–7^{c}

Structure 4^{c}

Torsion angle definitions: ϕ = C2'–C1'–O1'–C4; ψ = C1'–O1'–C4–C3

ϕ = 150°; ψ = 90°

C	2.904	0.311	3.217
C	2.196	0.055	1.878
C	1.174	1.181	1.621
C	2.311	2.742	3.015
C	3.443	1.742	3.314
H	2.927	0.075	1.065
H	2.174	0.186	4.035
H	1.514	2.634	3.767
H	4.241	1.875	2.569
H	0.371	1.134	2.381
O	1.793	2.442	1.714
O	0.620	0.974	0.344
C	-0.714	1.347	-0.066
C	-0.644	2.780	-0.627
C	-1.569	0.414	-0.954
H	-1.239	1.413	0.892
C	-2.026	3.334	-1.002
H	-0.019	2.759	-1.537
H	-1.136	0.272	-1.949
C	-2.791	2.281	-1.820
H	-2.602	3.517	-0.092
H	-2.290	2.079	-2.787
O	-2.840	1.074	-1.068
O	3.960	1.848	4.639
H	4.321	2.738	4.771
O	3.955	-0.633	3.353
H	4.376	-0.441	4.207
C	2.770	4.191	2.975
H	3.214	4.453	3.947
H	3.545	4.300	2.202
O	1.643	5.011	2.693
H	1.949	5.930	2.670
O	-0.071	3.653	0.334
H	0.798	3.277	0.550
C	-1.840	-0.935	-0.310
H	-0.886	-1.426	-0.091
H	-2.373	-0.770	0.638
O	-2.631	-1.707	-1.210

H	-2.795	-2.563	-0.786
O	-4.087	2.736	-2.024
C	-4.905	1.840	-2.778
H	-5.061	0.897	-2.245
H	-5.863	2.345	-2.922
H	-4.455	1.628	-3.759
C	-2.490	5.756	-1.223
O	-3.130	5.811	-0.171
C	-2.272	6.987	-2.086
H	-3.246	7.405	-2.360
H	-1.745	7.744	-1.496
H	-1.702	6.790	-2.998
C	1.909	-2.214	0.934
O	2.771	-2.049	0.070
C	1.145	-3.520	1.058
H	0.653	-3.734	0.104
H	1.857	-4.330	1.249
H	0.393	-3.517	1.852
H	0.862	-1.461	2.528
N	1.575	-1.252	1.842
H	-1.420	4.638	-2.565
N	-1.925	4.607	-1.689

Structure 5[c]
Torsion angle definitions: ϕ = C2'–C1'–O1'–C4; ψ = C1'–O1'–C4–C3
$\phi = 150^{o}$; $\psi = 150^{o}$

C	-0.230	1.174	0.610
C	-0.903	1.322	1.970
C	-0.860	2.805	2.333
C	0.577	3.285	2.327
C	1.208	2.995	0.973
C	2.696	3.309	0.943
C	-1.106	-2.424	-2.992
C	0.376	-2.329	-2.738
C	0.687	-1.747	-1.373
C	-0.144	-0.497	-1.086
C	-1.610	-0.730	-1.471
C	-2.436	0.544	-1.345
O	-0.110	-0.194	0.287
H	-0.805	1.712	-0.176
H	-0.326	0.760	2.742
H	-1.469	3.399	1.612
H	1.153	2.794	3.144
H	0.697	3.577	0.169

H	2.891	4.404	0.974
H	3.235	2.811	1.779
H	-1.583	-3.201	-2.355
H	0.855	-3.329	-2.864
H	0.507	-2.516	-0.587
H	0.244	0.347	-1.700
H	-2.056	-1.506	-0.803
H	-2.054	1.347	-2.014
H	-2.459	0.931	-0.303
H	-2.940	1.323	1.295
H	-4.553	-1.367	3.075
H	-4.440	-0.713	1.399
H	-4.836	0.389	2.782
N	-2.298	0.863	1.938
O	1.096	1.634	0.676
O	0.577	4.666	2.591
H	3.081	1.842	-0.271
O	2.065	-1.444	-1.385
O	-1.706	-1.167	-2.798
H	-1.371	3.959	3.798
C	-2.753	-0.204	2.662
C	-2.646	-2.836	-4.730
H	1.473	5.007	2.595
O	0.944	-1.481	-3.709
O	-1.416	3.018	3.606
O	-1.289	-2.720	-4.361
H	0.532	-1.657	-4.558
O	3.230	2.794	-0.261
H	2.284	-1.071	-0.523
O	-3.778	0.284	-1.709
C	-4.230	-0.490	2.469
O	-2.052	-0.866	3.387
H	-2.685	-3.072	-5.817
H	-3.185	-1.879	-4.556
H	-3.129	-3.667	-4.169
H	-4.295	1.089	-1.628

Structure 6[c]

Torsion angle definitions: ϕ = C2'–C1'–O1'–C2; ψ = C1'–O1'–C2–C1

$\phi = 165^o$; $\psi = 240^o$

C	1.492	-0.357	-0.503
C	2.372	0.787	-0.013
C	3.751	0.207	0.295
C	3.584	-0.898	1.317

C	2.630	-1.954	0.773
C	2.408	-3.113	1.735
C	-1.183	0.065	-2.605
C	-0.678	-0.707	-1.398
C	-1.844	-1.103	-0.510
C	-2.666	0.121	-0.171
C	-3.115	0.819	-1.446
O	0.175	0.119	-0.651
H	1.863	-0.768	-1.469
H	1.956	1.197	0.938
H	4.235	-0.180	-0.631
H	3.209	-0.471	2.275
H	3.039	-2.384	-0.173
H	1.702	-3.864	1.316
H	3.364	-3.625	1.985
H	-0.304	0.473	-3.159
H	-0.146	-1.624	-1.742
H	-2.478	-1.869	-1.015
H	-2.085	0.823	0.469
H	-3.791	0.146	-2.025
H	-4.801	1.902	-0.553
H	-3.245	2.826	-0.576
H	5.395	0.773	1.142
H	1.817	5.071	-1.770
H	1.692	3.661	-2.888
H	3.302	4.146	-2.209
O	1.860	-2.639	2.948
O	-1.367	-1.675	0.685
O	-1.904	-0.788	-3.468
H	1.436	-3.356	3.423
O	-2.005	1.152	-2.236
O	-3.773	-0.303	0.586
O	1.394	-1.364	0.481
C	1.998	3.119	-0.812
H	-2.118	-1.920	1.234
O	4.844	-1.457	1.590
N	2.476	1.853	-1.017
O	4.598	1.200	0.815
H	4.810	-1.919	2.431
H	-3.443	-0.862	1.296
O	-4.278	2.724	-2.341
C	-3.875	2.101	-1.137
H	2.852	1.617	-1.934
O	1.451	3.467	0.204
C	2.215	4.054	-1.986
C	-2.406	-0.139	-4.616

H	-2.932	-0.901	-5.234
H	-1.573	0.288	-5.216
H	-3.137	0.649	-4.336
H	-4.755	3.530	-2.129

Structure 7[c]

Torsion angle definitions: ϕ = C2'–C1'–O1'–C4; ψ = C1'–O1'–C4–C3

$\phi = 300^o$; $\psi = 300^o$

C	2.063	-0.822	-1.110
C	3.096	0.286	-1.207
C	4.479	-0.342	-1.067
C	4.531	-1.158	0.211
C	3.415	-2.196	0.227
C	3.393	-2.979	1.534
C	-0.077	2.525	1.527
C	-0.672	1.173	1.835
C	0.065	0.091	1.071
C	0.094	0.463	-0.405
C	0.539	1.912	-0.642
C	0.376	2.337	-2.097
O	2.176	-1.552	0.089
O	0.705	-0.461	-1.271
O	-0.222	2.789	0.149
O	5.473	0.651	-1.005
H	4.711	-0.983	-1.949
H	3.220	-2.310	2.407
H	1.605	2.062	-0.364
H	3.553	-2.914	-0.616
H	-1.760	1.166	1.587
H	0.989	2.586	1.838
H	2.329	-4.422	2.328
O	-0.842	3.504	2.197
O	-0.565	0.917	3.213
H	-1.033	1.606	3.692
C	-0.374	4.817	1.979
H	0.661	4.928	2.373
H	-0.412	5.074	0.898
H	-1.041	5.514	2.534
H	4.336	-3.545	1.696
H	1.003	1.727	-2.783
H	-0.684	2.277	-2.427
O	0.800	3.677	-2.255
H	0.695	3.937	-3.173
H	6.322	0.207	-0.917

H	-0.981	0.454	-0.729
H	2.984	1.026	-0.385
H	2.250	-1.538	-1.949
O	2.345	-3.928	1.505
O	-0.631	-1.113	1.290
H	-0.172	-1.808	0.809
H	1.082	-0.018	1.503
O	5.796	-1.771	0.271
H	5.875	-2.300	1.068
H	4.449	-0.493	1.102
H	3.016	0.816	-2.185

Brief Discussion of the Functional and Basis Sets Used in DFT Calculations

All DFT calculations for geometry optimization were conducted with the B3LYP functional and 6-31G* basis set. All DFT calculations of *J*-couplings in geometrically-optimized structures were conducted with the B3LYP functional and [5s2p1dl3s1p] basis set. The latter combination is fixed in that the basis set was developed for the B3LPY functional and no other functional can be substituted. The B3LYP functional and [5s2p1dl3s1p] basis set have been shown in previous studies by our laboratory to give accurate calculated ^{2}J and ^{3}J values involving carbon and hydrogen (errors of < 0.5 Hz) when the structures used in the calculations are optimized at the B3LYP/6-31G* level of theory (for example, see: W. Zhang, T. Turney, R. Meredith, Q. Pan, L. Sernau, X. Wang, X. Hu, R. J. Woods, I. Carmichael and A. S. Serianni, Conformational Populations of β-(1→4) *O*-Glycosidic Linkages Using Redundant NMR *J*-Couplings and Circular Statistics, *J. Phys. Chem. B* 2017, **121**, 3042–3058).

More specifically, we have examined several combinations of functionals and basis sets for geometry optimization, including B3LYP/6-31G*, B3LYP/6-311+G(d,p), ωB97XD/6-31G*, and ωB97XD/6-311+G(d,p) (see: T. Tetrault, R. J. Meredith, W. Zhang, I. Carmichael and A. S. Serianni, One-Bond ^{13}C-^{1}H and ^{13}C-^{13}C Spin-Coupling Constants as Constraints in *MA'AT* Analysis of Saccharide Conformation, *J. Phys. Chem. B* 2022, **126**, 9506–9515; T. Tetrault, R.J. Meredith, M.-K. Yoon, Canizares, C.; Oliver, A.G.; Carmichael, I. and A.S. Serianni, One-Bond ^{13}C-^{13}C Spin-Coupling Constants in Saccharides: A Comparison of Experimental and Calculated Values by Density Functional Theory Using Solid-State ^{13}C NMR and X-ray Crystallography, *Phys. Chem. Chem. Phys.* **2023**, *25*, 16048–16059), to determine their effects on calculated *J*-couplings. With the exception of ^{1}J values, the level of theory applied during geometry optimization exerts only small effects on calculated ^{2}J and ^{3}J values involving carbon and hydrogen. When $^{1}J_{CH}$ and $^{1}J_{CC}$ values are calculated, however, the level of theory used for geometry optimization does affect the calculated values significantly because C–H and C–C bond lengths are important determinants of these couplings.

Complete References 19 and 34

Reference 19

M. J. Frisch, G. W. Trucks, H. B. Schlegel, G. E. Scuseria, M. A. Robb, J. R. Cheeseman, G. Scalmani, V. Barone, G. A. Petersson, H. Nakatsuji, X. Li, M. Caricato, A. V. Marenich, J. Bloino, B. G. Janesko, R. Gomperts, B. Mennucci, H. P. Hratchian, J. V. Ortiz, A. F. Izmaylov, J. L. Sonnenberg, D. Williams-Young, F. Ding, F. Lipparini, F. Egidi, J. Goings, B. Peng, A. Petrone, T. Henderson, D. Ranasinghe, V. G. Zakrzewski, J. Gao, N. Rega, G. Zheng, W. Liang, M. Hada, M. Ehara, K. Toyota, R. Fukuda, J. Hasegawa, M. Ishida, T. Nakajima, Y. Honda, O. Kitao, H. Nakai, T. Vreven, K. Throssell, J. A. Montgomery Jr., J. E. Peralta, F. Ogliaro, M. J. Bearpark, J. J. Heyd, E. N. Brothers, K. N. Kudin, V. N. Staroverov, T. A. Keith, R. Kobayashi, J. Normand, K. Raghavachari, A. P. Rendell, J. C. Burant, S. S. Iyengar, J. Tomasi, M. Cossi, J. M. Millam, M. Klene, C. Adamo, R. Cammi, J. W. Ochterski, R. L. Martin, K. Morokuma, O. Farkas, J. B. Foresman and D. J. Fox, *Gaussian* 16, Revision B.01, Gaussian, Inc., Wallingford, CT, 2016.

Reference 34

D. A. Case, V. Babin, J. T. Berryman, R. M. Betz, Q. Cai, D. S. Cerutti, T. E. I. Cheatham, T. A. Darden, R. E. Duke, H. Gohlke, A. W. Goetz, S. Gusarov, N. Homeyer, P. Janowski, J. Kaus, I. Kolossváry, A. Kovalenko, T. S. Lee, S. LeGrand, T. Luchko, R. Luo, B. Madej, K. M. Merz, F. Paesani, D. R. Roe, A. Roitberg, C. Sagui, R. Salomon-Ferrer, G. Seabra, C. L. Simmerling, W. Smith, J. Swails, R. C. Walker, J. Wang, R. M. Wolf, X. Wu, P. A. Kollman, 2014, AMBER 14, University of California, San Francisco.
